# Study on knowledge about associated factors of Tuberculosis (TB) and TB/HIV co-infection among young adults in two districts of South Africa

**DOI:** 10.1371/journal.pone.0217836

**Published:** 2019-06-06

**Authors:** Simukai Shamu, Locadiah Kuwanda, Thato Farirai, Geoffrey Guloba, Jean Slabbert, Nkhensani Nkhwashu

**Affiliations:** 1 Foundation for Professional Development, Health Systems Strengthening Division, Pretoria, South Africa; 2 University of the Witwatersrand, School of Public Health, Johannesburg, South Africa; Sefako Makgatho Health Sciences University, SOUTH AFRICA

## Abstract

South Africa ranks third among 22 high burden countries in the world. TB which remains a leading cause of death causes one in five adult deaths in South Africa. An in-depth understanding of knowledge, attitudes and practices of young people towards TB is required to implement meaningful interventions. We analysed young men and women (18–24 years)’s TB knowledge including TB/HIV coinfections, testing rates and factors associated with them. A cross sectional cluster-based household survey was conducted in two provinces. Participants completed computer-assisted self-interviews on TB knowledge, testing history and TB/HIV coinfections. A participant was regarded as knowledgeable of TB if s/he correctly answered the WHO-adopted TB knowledge questions. We built three multivariate regression models in Stata 13.0 to assess factors associated with knowing TB alone, testing alone and both knowing and testing for TB. 1955 participants were interviewed (89.9% response rate). Their median age was 20 years (IQR19-22). Sixteen percent (16.2%) of the participants were social grant recipients, 55% were enrolled in a school/college and 5% lived in substandard houses. A total of 72% had knowledge of TB, 21% underwent screening tests for TB and 14.7% knew and tested for TB. Factors associated with TB knowledge were being female, younger, a student, social grant recipient, not transacting sex and having positive attitudes towards people living with HIV (PLWH). Factors associated with TB testing were being a student, receiving a social grant, living in OR Tambo district, HIV knowledge and having a family member with TB history. Factors associated with both TB knowledge and testing were being female, a student, using the print media, living in OR Tambo district and having a family member with a TB history. The study demonstrates the importance of demographic factors (gender, economic status, family TB history, and location) and HIV factors in explaining TB knowledge and testing. We recommend extending community TB testing services to increase testing.

## Introduction

About 10 million individuals developed *Mycobacterium tuberculosis bacillus* (TB) disease globally and 1.6 million died in 2017[1]. Eighty percent of the world’s burden of tuberculosis (TB) is carried by twenty-two countries defined as high burden countries by the World Health Organization (WHO) and South Africa is one of them[2]. In South Africa 692 individuals per 100 000 people are diagnosed with TB[3].

In Sub-Saharan Africa TB is the most common cause of morbidity and death among people living with HIV[4]. Almost 20% of the world’s reported HIV-associated TB cases are found in South Africa[2] where up to 73% of all TB cases are co-infected with HIV. HIV-associated TB deaths in South Africa are as high as 121.7 per 1000 among co-infected antenatal patients compared to only 38.5 deaths per 1000 population in those not co-infected[5]. The mortality rates are even higher in patients with drug resistant strains. In a recent study in South Africa almost all extensively drug resistant TB patients died within an average of 16 days from diagnosis time[6]. A lower cure rate of about 67%[3] which is far below the 85% recommended by the WHO not only signifies a sustaining burden in the country[7] but also accounts for high death rates.

Integrating TB and HIV services to ensure 90% of TB patients are tested for HIV and HIV patients are screened for TB[8] can reduce at least 10 000 deaths each year.[9] Priority steps to control TB have been outlined and include increasing cure rates to 85%, improving case detection rates to 70%, integrating TB with HIV, and identifying and treating drug resistant TB[9]. However, there is need to ensure that knowledge and attitudes at the population level are understood and if low, then improved to optimal levels for sustainable TB control. In line with the National Strategic Plan for HIV, STI and TB, understanding the population’s TB knowledge and practices is the first step towards preventing and managing the disease. There is need to assess if the population understands TB symptoms, transmission and HIV/TB co-infections at a population level.

A systematic review of qualitative studies on patient adherence to TB treatment outlined eight major barriers to successful TB management globally and these include TB knowledge, attitudes and practices[10]. Knowledge and attitudes towards the disease are important determinant factors at every stage of the TB cascade including diagnosis, accepting test results, social support, and adherence[11,12]. For example, knowledge of TB should result in identifying symptoms and getting a TB test based on identified symptoms and then acting on the results of the TB test [12]. It is therefore important to establish TB knowledge levels of the people in the community so that relevant interventions can be made to control TB. Health behavior theories explain that for one to be tested and take care of themselves against a disease, they must first of all know and perceive that the disease is dangerous, and that they are at risk[13]. Although health providers’ knowledge of TB symptoms, transmission and diagnosis is higher in South Africa than in most other high burden countries[14,15] TB knowledge in the general population is however not adequately understood. There is need to assess the general public’s awareness and knowledge of TB.

Young people aged 10 to 24 years constitute 27% of the global population. The size of this population, which constitutes the largest cohort, requires huge health investment through research and programme implementation[16]. In addition, it is understood that important health problems and risk factors for diseases including TB and HIV emerge at this age while these adolescents are generally thought to be healthy[16]. Mortality from HIV and TB which constitutes 11% cause of death among young people aged 10–24 years[17] is a cause for concern which warrants continued research. A systematic review of the cause of disability-adjusted life-years (DALY) among 20–24 year olds globally shows that TB is number seven cause of DALYs[16] making TB in young people a key target for research and implementation. Understanding young people’s knowledge of TB is therefore an important aspect towards reducing morbidity and mortality including in later life. While TB affects all people, 90% of the cases in 2017 both young people and adults aged ≥15 years and 9% were people living with HIV of which more than 70% were from Africa[16]. Research and interventions should therefore include the youth as well. With young people being more susceptible to HIV whose co-infection with TB causes many deaths and high morbidity, there is need to assess their knowledge of TB and TB/HIV coinfections. The aim of this paper is therefore to assess rates of TB knowledge including TB/HIV coinfections and factors associated with TB knowledge and testing among young people in South Africa.

## Methods

### Design and setting

This was a cross sectional study of young men and women aged 18 to 24 years in two districts—Nkangala in Mpumalanga Province and OR Tambo in the Eastern Cape Province between October 2017 and January 2018. In South Africa the Eastern Cape Province had the highest incident cases of 692 per 100 000 people in the country with a treatment success rate of 76.2% while Mpumalanga has a case rate of 402 per 100000 people and 84% treatment success rate in 2015 (https://www.tbfacts.org/tb-statistics-south-africa/. Accessed 20 October 2018). The study was conducted in low income urban, peri-urban and rural communities predominantly occupied by black South Africans. The study used a multi-stage cluster sampling methodology. Five districts with a high HIV burden in the two provinces were selected and the study was conducted in two of these districts (Nkangala district in Mpumalanga and OR Tambo district in the Eastern Cape Province). The sample size was calculated as per sample size for prevalence survey with finite population correction. For a precision of 5%, power of 80% and design effect of 1.5 the minimum sample size calculated was 1826 participants. Simple random selection of health facilities in the districts was done. The selected facilities were used as the first stage clusters for the catchment areas in which the study was conducted while the second stage clusters were the catchment areas served by the selected seven health facilities in Nkangala district and 15 facilities in OR Tambo district. We randomly selected one out of at least four areas (wards) served by each facility. From each of the seven areas (wards) we consecutively recruited at least 130 young men and women (ratio 1:1) in OR Tambo district and at least 62 young men and women (ratio 1:1) in the 15 areas in Nkangala district to reach a minimum sample size of 1826 men and women. Using a town or rural planning map with numbered residential stands, eligible participants were consecutively recruited from the first numbered residential stand until the sample size was reached for each ward.

### Questionnaire development

We designed a questionnaire with a number of questions and scales for data collection. TB knowledge questions were adapted from the WHO TB knowledge items[18]. Eight questions were presented covering TB transmission, treatment and con-infection with HIV. A typical question read: “Can a person get TB from the air when a person with TB coughs or sneezes?” Responses were Yes or No. Answering at least 6 questions correctly was regarded as knowledgeable of TB. TB testing was assessed by asking participants if ever they tested for TB. A Yes response indicated that they ever tested for TB while a No indicated never tested for TB. We assessed participants’ knowledge of HIV by asking five questions following the conceptualization of the UNAIDS[19] that was used repeatedly in South Africa[20]. A typical HIV prevention question read: “To prevent HIV infection, a condom must be used for every round of sex’. Only participants who answered all questions correctly were regarded as knowledgeable of HIV. Attitudes towards people living with HIV was measured using four stigma questions. Participants indicated their level of agreement with each statement using a 4-point Likert scale (from 1 = "strongly disagree", 2 = “disagree”, 3 = “agree” to 4 = "strongly agree"). A typical question asked was “I would stay friends with someone even if I found out that he/she has HIV”. Participants’ individual scores were added and scoring at least 7 was regarded as positive or accepting attitudes towards people living with HIV (PLWH). HIV testing attitudes were assessed by asking participants their views about their community perceptions on HIV testing[21]. A typical community HIV testing attitude statement was “People in my community do not test for HIV because they are scared that they are already HIV positive”. Participants indicated their level of agreement with each statement using a 4-point Likert scale (from 1 = "strongly disagree" to 4 = "strongly agree")[22]. We combined the individual scores for all the questions for each participant and at least 12 scores were regarded as having positive or accepting attitudes. We also asked if participants ever had transactional sex, tested for HIV, their test results as well as their HIV risk perception. The questionnaire also contained a range of demographic questions including ever having a member of their household with TB, gender, age, occupation, education, socio-economic characteristics (possession of household goods, income source) as well as their use or non-use of the media for health purposes.

### Data collection

The questionnaire was translated to isiNdebele and isiXhosa and back translated for clarity and correctness by an independent expert. The questionnaire was administered in either English, isiNdebele or isiXhosa depending on a participant’s preferred language. We mapped the study communities to enable our research assistants to move door to door using a community residential map to avoid missing any household until the sample size was reached in each cluster. The questionnaire was set into a RedCap system for data collection[23]. RedCap is an electronic platform that makes self-administering of the questionnaire easy, enables sensitive questions to be asked privately and facilitates saving and backing up data correctly while minimizing the challenges[23,24]. Male and female local fieldworkers in their twenties were recruited and trained before deployment. The data collection procedures and instruments were pretested in the communities resulting in only minor language corrections done. Pilot data were therefore included in the analysis. Daily tallying of participants enrolled was done to ensure a balance of participants enrolled in terms of gender and cluster as outlined in our minimum required sample size.

### Data analysis

Data were exported to Stata 13.0[25] for analysis. We conducted regression analyses to assess factors associated with TB variables in three models. The first model assessed factors associated with TB knowledge, the second model assessed factors associated with TB testing. In the third model, we constructed a variable called TB knowledge and testing in which participants were categorized as either having TB knowledge and having tested for TB before or not. The selection of variables for each model was based on our knowledge of the literature on possible associations and our bivariate analyses. Following Hosmer and Lemeshow’s advice on variable selection for logistic regression[26], variables with a p value of < .250 in bivariate associations with TB knowledge or testing were then entered into each model. We also controlled for possible confounders including age and socioeconomic status in each model. The models were built using a stepwise regression approach and the outputs were presented as adjusted Odd Ratios (aORs).

### Ethics

The study received Ethics Approval from the Foundation for Professional Development Research Ethics Committee. All participants interviewed provided written informed consent. Participants did not receive an incentive for participation.

## Results

A total of 1955 participants were recruited and interviewed (response rate of 89.9%) in Mpumalanga (n = 973) and Eastern Cape (n = 982) provinces. Overall 50.2% of the sample were female. Median age of the participants was 20 years (IQR19-22). A total of 16.2% participants were social grant recipients. Fifty-five percent of the participants were enrolled in school. Of the 45% out of school 82.3% were unemployed. Thirteen percent were either married or partnered. One in 20 participants (5%) lived in substandard housing including Wendy house or shack. The radio (56.4%) was the most preferred media channel for TB information dissemination followed by the television (52.1%). Overall 72.1% were knowledgeable of TB. Slightly over 1 in five (22.1%) participants reported ever testing for TB. Fifteen percent (14.7%) had correct knowledge of TB and had ever tested for TB. Table 1 shows levels of TBknowledge per question asked and as a combined variable. Participants answered most of the symptoms and transmission questions correctly compared to the questions on co-infections.

**Table 1:** Levels of TB knowledge (N = 1955).

	Correct answer	n	%
Anybody can get TB	Yes	1577/1909	82.7
People living with HIV are more likely to get TB	Yes	1235/1900	65.0
People that are HIV negative can get TB	Yes	1437/1892	76.0
Can a person get TB through the air when a person with TB coughs or sneezes	Yes	1658/1927	86.0
Are people with TB always HIV positive?	No	1275/1907	66.9
What is the treatment for TB?	Daily TB drugs for six mo	1658/1877	88.3
Is it possible to cure TB in people with HIV?	Yes	1183/1900	62.3
**Overall TB treatment knowledge score (6–8 questions):**		1278/1772	72.1

Table 2 shows demographic characteristics by TB knowledge, TB testing and both TB knowledge and testing.

**Table 2:** Characteristics of participants by TB knowledge, testing and both knowledge and testing.

	TB knowledge (72.1%)			TB Testing (22.1%)			TB Knowledge & testing (14.7%)		
	n/N	%	p value	n/N	%	p value	n/N	%	p-value
Age (median, (IQR))		21 (19–22)			21 (19–22)			21 (19–22)	
Married/partnered/lives with partner (vs single)	177/235	75.3	0.257	66/235	28.1	0.019	43/250	17.2	0.241
Occupation: student (vs out of school)	548/783	70.0	0.074	205/761	26.9	<0.0001	143/863	16.6	0.039
Member of a social club (vs not a member)	535/733	73.0	0.558	186/747	24.9	0.027	378/787	48.0	0.126
Receiving a social grant (vs no grant)	186/292	63.7	<0.0001	86/288	29.9	0.001	55/308	17.9	0.109
Education: Completed Matriculation (vs no Matric)	751/930	58.8	<0.0001	201/947	50.9	0.302	161/1003	56.9	0.084
Income source:									
Employer	108/144	8.6		37/141	9.6		31/157	11.2	
Family/partner	1011/1360	80.4		274/1391	71.0		207/1459	74.5	
Social Grant (vs no social grant)	139/239	78.0	<0.0001	75/226	19.4	<0.0001	40/280	14.4	0.171
Lives in a sub-standard house (vs standard)	58/93	62.4	0.030	22/91	24.2	0.611	13/99	13.1	0.660
Possesses 5+ basic commodities (vs <5)	887/1116	69.4	<0.0001	212/1138	53.7	<0.0001	177/1181	62.5	0.638
Gender: Female (vs male)	645/878	73.5	0.235	224/894	57.1	0.002	165/962	17.2	0.002
Living in OR Tambo (vs Nkangala district)	627/865	72.5	0.739	234/852	27.5	<0.0001	167/959	17.4	0.001
Using print media for health messages (ref: Non-use)	960/1266	75.8	0.037	312/386	80.8	0.007	229/277	82.6	<0.0001
HIV prevention knowledge score (high)	663/1232	53.8	<0.0001	150/385	39.0	<0.0001	131/275	47.6	0.290
Knowledge of pre-exposure prophylaxis (ref: No)	219/1228	17.8	0.379	87/368	23.6	<0.0001	64/266	24.06	0.001
Transactional sex (ref: No)	152/1154	13.2	<0.0001	68/367	18.5	0.083	46/261	17.6	0.402
Positive attitudes towards PLWH (ref: Neg attitudes)	1219/1260	96.8	<0.0001	365/385	94.8	0.958	267/278	96.04	0.037
Positive attitudes towards HIV testing (ref: Neg attitudes)	904/1197	75.5	0.061	261/359	72.7	0.119	194/266	72.9	0.218

### TB knowledge

Participants who received a social grant were more likely to be knowledgeable of TB than those who did not receive a social grant (p<0.0001). Participants who completed matriculation were more likely to be knowledgeable of TB than participants who did not complete matriculation (p<0.0001). Regarding accommodation type participants living in substandard housing were more likely to report higher knowledge of TB than those living in standard housing. In terms of basic household possessions, participants who possessed at least five basic commodities were more likely to be knowledgeable of TB than those who had less than five commodities (p<0.0001). Participants whose income came from family/partners and social grants were more likely to be knowledgeable of TB compared to those who were employed (p<0.0001). Participants using the print media, with higher levels of HIV knowledge, engaging in transactional sex and those who had positive attitudes towards PLWH were more likely to be knowledgeable of TB than those not using the print media, with less HIV knowledge, not transacting in sex or with negative attitudes towards PLWH.

### TB testing

We assessed the relationship between TB testing and a number of socio-demographic variables. Participants who reported being married, partnered or living with partners were more likely to report having tested for TB than those who were single (p = 0.019).

Participants who were students were more likely to have tested for TB than those participants who were out of school (p<0.0001). Being a member of any social club compared to non-membership was associated with ever testing for TB (p<0.027). Participants who were social grant recipients were more likely to have tested for TB than those who survived on paid work or family income (p<0.0001). In addition, more participants in possession of at least five basic household goods ever tested for TB compared to those with less than five possessions (p<0.0001). Participants who were female (p = 0.002) and living in OR Tambo (p<0.0001) were more likely to report testing for TB than those who were male or living in Nkangala respectively. Participants using the print media, those with higher levels of HIV knowledge and those with knowledge of pre-exposure prophylaxis (PrEP) were more likely to have tested for TB. Of those who had an HIV test, 8.1% tested HIV positive. HIV positive people were less likely to test for TB than HIV negative people (45.6% vs 54.4%; p = 0.034). Also 21.6% of the participants perceived themselves to be at high risk of HIV infection. Fewer participants who perceived themselves to be at high risk of HIV infection tested for TB than those who perceived to be at low risk (20.39% vs 26.96%; p = 0.010) (data not shown on table).

### Knowledge and testing for TB

We constructed a variable for participants who both knew TB and ever tested for TB and conducted a test of differences. Participants who reported being students were more likely to report knowing and testing for TB (p = 0.039) than those who were out of school. Female participants were more likely to report knowledge of and testing for TB than male participants (p = 0.002) while participants who lived in OR Tambo district were more likely to report knowing and testing for TB than those who lived in Nkangala district (p = 0.001). Participants using the print media were more likely to know and test for TB (p<0.0001) than those who did not. Participants with knowledge of PrEP were more likely to know and test for TB (p<0.001) than those who did not know PrEP.

### Factors associated with TB knowledge

We conducted logistic regression analysis to assess factors associated with having correct TB knowledge. Table 3 shows factors associated with TB knowledge. Being under 21 years (aOR1.44, 95% CI: 1.06–1.95), female (aOR 1.47, 95%CI: 1.11–1.95), a student (aOR 0.69 95% CI: 0.51–0.94), social grant recipient (aOR 0.58 95%CI: 0.41–0.83), not transacting sex (aOR 0.51, 95%CI: 0.36–0.73) and having positive attitudes towards PLWH (aOR 3.72, 95%CI: 2.11–6.57), high HIV prevention knowledge score (aOR 2.76 95%CI: 2.08–3.66) were associated with having high TB knowledge.

**Table 3:** Multivariate analysis showing factors associated with TB knowledge, TB testing and both TB knowledge and testing in Nkangala and OR Tambo districts.

	TB Knowledge	TB Testing	TB Knowledge and testing
Factors	aOR (95% CI)	aOR (95% CI)	aOR (95% CI)
Age: 18–20 years (ref = 21-24years)	1.44 (1.06–1.95)	NS	NS
Gender: Female (vs male)	1.47 (1.11–1.95)	NS	1.42 (1.03–1.96)
Occupation: Student (vs out of school)	0.69 (0.51–0.94)	1.71 (1.28–2.30)	1.44 (1.05–1.97)
Living in OR Tambo (vs Nkangala district)	NS	1.83 (1.35–2.47)	1.50 (1.08–2.09)
Receiving a social grant (vs no grant)	0.58 (0.41–0.83)	1.61 (1.13–2.31)	NS
No household member ever had TB (ref: Yes)	NS	0.21 (0.16–0.28)	0.19 (0.14–0.27)
Using print media for health messages (ref: Non-use)	NS	NS	1.63 (1.07–2.47)
HIV prevention knowledge score (high)	2.76 (2.08–3.66)	0.73 (0.55–0.97)	NS
Knowledge of pre-exposure prophylaxis (ref: No)	NS	0.67 (0.47–0.97)	NS
Transactional sex (ref: No)	0.51 (0.36–0.73)	NS	NS
Positive attitudes towards PLWH (ref: Neg attitudes)	3.72 (2.11–6.57)	NS	NS
Positive attitudes towards HIV testing (ref: Neg attitudes)	NS	0.66 (0.48–0.91)	NS

### Factors associated with TB testing

In the second model we assessed factors associated with ever testing for TB. Table 3 shows the results of this analysis. We found that being a student (aOR 1.71 95% CI: 1.28–2.30), receiving a social grant (aOR1.61 95%CI: 1.13–2.31), living in OR Tambo district (aOR 1.83 95%CI: 1.35–2.47), having HIV knowledge (aOR 0.73: 0.55–0.97), having a family member with a TB history (aOR 0.21, 95%CI: 0.16–0.28) and having positive attitudes towards HIV testing (aOR 0.66, 95%CI: 0.48–0.91) were associated with ever testing for TB.

### Factors associated with TB knowledge and testing

In the third model we combined TB knowledge and TB testing and assessed factors associated with both TB knowledge and testing. We found that being female (aOR 1.42 95%CI: 1.03–1.96), a student (aOR 1.44, 95% CI: 1.05–1.97), using the print media (aOR 1.63, 95%CI: 1.07–2.47), living in OR Tambo district (aOR 1.50, 95%CI: 1.08–2.09) and having a family member with a TB history (aOR 0.19, 95% CI: 0.14–0.27) were associated with both knowing and testing for TB.

## Discussion

In this paper we sought to identify rates of and associated factors for TB knowledge and testing among young people in two districts of South Africa. We found almost three quarters of the participants knowledgeable of TB (72%) and one in five having ever tested for TB. Only 14.7% knew and tested for TB. Demographic factors were associated with both TB knowledge and testing while HIV-related factors were associated with TB knowledge alone or TB testing alone. Demographic factors related to TB knowledge and testing were being female, a student, living in OR Tambo district, having a household member who had TB before and using the print media for health messages. HIV-related factors such as high HIV knowledge, PrEP knowledge, transactional sex, having positive attitudes towards PLWH and towards HIV testing were associated with either TB knowledge alone or TB testing alone in different ways as shall be discussed. This study contributes to our knowledge of the importance of demographic factors and TB/HIV connections as communicable diseases in the prevention of TB as a leading killer disease.

We found good knowledge levels of TB transmission, cure and coexistence with HIV in the study. Elsewhere in a South African national community-based study[27], just as we found, almost three quarters were knowledgeable of TB. However, it is concerning that questions related to TB/HIV co-infections were the least correctly answered showing lower levels of HIV/TB integration awareness. Having low knowledge of these co-infections when co-infected individuals have the highest mortality requires more attention including information dissemination and education at a population level. In South Africa, the knowledge of HIV/TB coinfections among health professionals increased only after 2005[28] and the National Strategic plan for HIV, STIs and TB laments the low levels of awareness of TB/HIV coinfections among the general public in South Africa[29]. Our finding confirms these low knowledge levels of the coinfections among the general public. Also that fewer PLWH or fewer participants who perceived themselves to be at increased HIV risk screened and tested for TB is concerning. In high burden districts as in the Eastern Cape, screening and testing of TB symptomatic individuals needs to increase to ensure that the WHO’s End TB Strategy[30] targets are met. A number of interventions such as marketing of the testing programmes, developing positive attitudes towards screening for TB in TB prevalent communities and availing testing opportunities are needed to increase demand for TB knowledge, screening and testing of symptomatic individuals.

The study highlights the importance of socio-demographic factors in determining the knowledge of screening and testing for TB. A national study in South Africa also found a similar relationship between demographic factors and TB knowledge[27]. Naidoo et al found that being men, educated, having a TB history in the family, and having access to the media were TB knowledge determinants[27]. However, we found women being more knowledgeable than men, in contrast to findings from previous studies conducted in South Africa[27] and other countries[31,32]. This could be due to women’s high health seeking behavior including opportunities in antenatal care visits where they get information on HIV/TB coinfections[33–35]. With the national burden of TB skewed against men, urgent interventions to target men with information dissemination are required.

We found that a history of TB in the family increases one’s chances of knowing TB. This could be because treatment efforts to people with TB include intensifying case finding and reaching out to family members of the index patient with testing, knowledge dissemination [36] and for care and support. These factors may have led to many participants with a TB patient in the family to know TB than those who did not. Our finding that poverty is associated with TB knowledge and testing confirms a known relationship[37] but in a different way. We found that receiving a social grant was protective of knowing TB. Receiving a social grant is a proxy for being in a low income group which the government intervenes by providing grants to the vulnerable people. Poverty may prevent one from acquiring important knowledge due to restricted access to the media sources (TV, radio, internet) hindering screening for symptoms. However, social grant recipients had higher odds of testing for TB than non-grant recipients which could show that they may have been targeted by interventions leading to screening and testing for TB. Secondly, those receiving social grants may have visited public clinics for other health conditions[37] where they had an opportunity to screen and test for TB.

Demographic factors that are associated with TB knowledge and testing help us to understand factors to target for TB control. For example knowing that people living in OR Tambo district had higher odds of TB testing than those in Nkangala district does not surprise us because OR Tambo is in the Eastern Cape Province which has many districts described as high burden TB districts in South Africa. About 1 in 3 people report living in a household where one has had TB before in the Eastern Cape Province[38] whose many areas are characterized by poverty and unemployment. The province also has high rates of multi-drug resistant TB (MDR-TB) and extensively drug-resistant TB[39], has treatment success rates (41%) that are far below the national rates[40]. Due to this burden, increased resources have been targeted to these districts including government and donor funding and research institutions have actively intervened to control the disease.

Although being a student presented less odds of knowing TB compared to being out of school–possibly because of increased exposure to TB amongst contacts since TB incidence increases with age as most of the out of school youth had already completed school—being a student increased the odds of testing, and both having knowledge of and testing for TB. It is possible that the basic lessons on TB prevention messages and related testing interventions in schools contributed towards the higher rates of testing for TB among students[41] as found in our study.

Intervening at the level of socio-structural factors associated with TB in general has been reported as much more difficult[27] than intervening at the level of behavioural factors. Nevertheless, since the study has highlighted the preferred media channels and the channels associated with knowledge of and testing for TB, the health media houses can plan relevant messages and accelerate its dissemination to contribute towards efforts to educate people to prevent TB. Using the print media was associated with TB knowledge and testing. As reported elsewhere[27,32] the print media was significantly related to TB knowledge and testing although the radio and the television were the preferred media channels for TB and HIV messages. Use of the print media could also be a proxy for access to educational material in schools as most of the areas surveyed did not use electronic media in schools and were low income communities whose access to these gadgets was minimal.

The National Strategic Plan for HIV, STI and TB calls for the integration of HIV, STI and TB services[42]. This is being implemented by a number of community-based organizations and NGOs assisting the department of health in strengthening South African health systems. We assessed if HIV factors were associated with TB issues. Indeed, we found associations between HIV factors and TB knowledge or TB testing. High level of HIV knowledge and positive attitudes towards PLWH were associated with TB knowledge. However the direction of the association on the HIV factors and TB testing alone is worrisome–a high HIV knowledge score, knowledge of PrEP and having positive attitudes towards HIV testing were negatively associated with TB testing implying that those who knew HIV did not screen and test for TB, possibly due to fear to know their results. Further, we reported that a significant proportion of HIV positive people and people reporting high HIV risk perception did not test for TB. It is therefore concerning that those who know, perceive that they are at high HIV risk or actually tested positive to HIV do not screen or test for TB when the two infections make a deadly combination in patients[43,44]. It calls for increased awareness of TB/HIV coinfection and integration in management.

The study has its own limitations. Firstly, the research design was cross sectional which limits any causal inferences because the relationships are temporal. Cross sectional studies assess exposures and outcomes simultaneously making it difficult to determine causality. Secondly, the study did not verify participants’ claims that they tested for TB or for HIV. We only relied on their reported data. Given that participants may choose to disclose or not the information about having tested or not, our results must therefore be interpreted with caution. In addition, we did not ask if participants screened for TB in this study. This information could have helped us to know if there were participants who did not test but had TB symptoms. One of the strengths of the study was a large sample size overall, coupled with high response rate for adequate power in our analyses. Collecting data in clusters covering urban, semi urban and rural settings enabled us to recruit participants with characteristics representative of communities and districts where the study was conducted thereby making possible comparisons with data from other household surveys conducted in South Africa.

## Conclusions

The study found high levels of TB knowledge amongst youth overall but low levels of knowledge of TB/HIV coinfections. Demographic factors (age, gender, place of residence, occupation) and socio-economic status were associated with TB knowledge or testing while HIV-related factors (accepting attitudes towards HIV testing or PLWH, knowledge of PrEP and high HIV prevention knowledge score) were associated with TB knowledge alone or TB testing alone. Interventions on TB should consider strengthening the education of the people about TB/HIV coinfections in the communities towards a comprehensive programme of TB prevention including testing. Holistic interventions that target altering socio-economic characteristics of the communities remain key in ensuring that high levels of TB knowledge and testing are achieved. In a nutshell, this paper contributes to our knowledge of the importance of demographic factors and TB/HIV integration when planning TB control programmes.

## Supporting information

S1 File English questionnaire. (DOCX)

S2 File Translated questionnaire. (DOCX)

S3 File Data dictionary. (PDF)

S4 File Data set. (DTA)

S5 File Survey data. (DTA)
