# Supplementary material for: Study on knowledge about associated factors of Tuberculosis (TB) and TB/HIV co-infection among young adults in two districts of South Africa
Source: PLoS One. 2019 Jun 6;14(6):e0217836. doi: 10.1371/journal.pone.0217836 (PMC6553726; doi:10.1371/journal.pone.0217836)
Supplement: S1 File — (DOCX) [file pone.0217836.s001.docx]

**Knowledge, attitudes and practices of young people towards HIV/TB prevention, risk tolerance, testing and treatment in Nkangala (Mpumalanga Province) and OR Tambo (Eastern Cape Province) districts**

**KAP Survey Questionnaire**

| **SECTION A : INTERVIEW SETUP AND IDENTIFICATION**  **This section is completed by the interviewer** | | | | | | | | | | |
| --- | --- | --- | --- | --- | --- | --- | --- | --- | --- | --- |
| **A1 : GEOGRAPHIC** | | | | | | | | | | |
|  | Province | | | | | Mpumalanga  Eastern Cape | | | | 1  2 |
|  | District | | | | | Nkangala  OR Tambo | | | | 1  2 |
|  | Household questionnaire number | | | | |  | | | | |
|  | Person number of respondent | | | | |  | | | | |
|  |  | | | | | | | | | |
| **A2 : INTERVIEW PARTICULARS (Section A is completed by the interviewer)** | | | | | | | | | | |
|  |  | Day | Month | Year | Time code | | | Response code | | |
|  | First visit |  |  |  | __ : __ | | |  | | |
|  | Second visit |  |  |  | __ : __ | | |  | | |
|  | Third visit |  |  |  | __ : __ | | |  | | |
|  | Final response code | | | | | | | |  | |
|  |  | | | | | | | | | |
| **Response code** 1 = Interview completed  2 = Appointment made for interview  3 = Selected respondent not at home 4 = Refusal by head of household  5 = Refusal by respondent 6 = Other | | | | | | | | | | |
|  |  |  |  |  |  | | |  | | |
| Visit Notes (Comments) | | | | | | | | | | |
|  | | | | | | | | | | |
|  | INTERVIEW STARTING TIME: | | | | | | __ : __ | | | |
|  | ITERVIEW END TIME : | | | | | | __ : __ | | | |
|  | INTERVIEWER NAME: | | | | | |  | | | |

| **REFUSAL PARTICULARS (IF APPLICABLE) (Section B is completed by the interviewer)** | | | | | | |  |  |  |  |  |  |  |  |
| --- | --- | --- | --- | --- | --- | --- | --- | --- | --- | --- | --- | --- | --- | --- |
|  |  | | | | | | |  |  |  |  |  |  |  |
| **Q#** | **Question** | **responses** | **code** | | **skip** | |  |  |  |  |  |  |  |  |
| A3 | At what point did the respondent refuse? | | | | | |  |  |  |  |  |  |  |  |
|  | At the gate or door After explanation of the survey and the process  After the first respondent has been identified (before interview) During the individual interview After the individual interview when requested to do the test  Other_____________________________________________________ | | | 1 2 3 4 5 6 | |  |  |  |  |  |  |  |  |  |
| A4 | Why don't you want to take part in the study? [You don't have to tell me]. | | | | | |  |  |  |  |  |  |  |  |
|  | **Upfront refusals** | | | 1 2 3 4 5 6 7 8 9 10 | |  |  |  |  |  |  |  |  |  |
|  | Too busy to grant interview Not available now Too late in the evening Not willing to participate in any survey/interview Objected to the topic of the survey (HIV) Objected to being interviewed by the specific interviewer Afraid Fear a breach of confidentiality Government is not doing enough for him/her Other________________________________________________________________ | | |  |  |  |  |  |  |  |  |  |  |  |
|  | **Refusals during individual interview** | | | 11 12 13 14 15 16 17 18 | |  |  |  |  |  |  |  |  |  |
|  | Objected to providing any/some information on the topic Objected to providing personal/confidential information Unable to provide requested information Refused to continue because he/she got irritated/bored Refused to continue because he/she got angry Refused to continue because he/she lost interest or got tired Refused to continue because he/she was in a hurry Other_________________________________________________________________ | | |  |  |  |  |  |  |  |  |  |  |  |
| **Refusal Notes** | | | | | | |  |  |  |  |  |  |  |  |
|  | | | | | | |  |  |  |  |  |  |  |  |

| **Q#** | | **Description** | | **Response** | **Code** |
| --- | --- | --- | --- | --- | --- |
| A5 | | The respondent is in the room with no others present except for the interviewer. | | Yes  No | 1  2 |
| A6 | | I have read the individual information sheet, statement of confidentiality and informed consent form | | Yes  No | 1  2 |
| A7 | | If the participant agreed to participate, did he/she sign the consent form? | | Yes  No | 1  2 |
| A8 | | Has the participant retained a copy of the information sheet? | | Yes  No | 1  2 |
| **SECTION 1: RESPONDENT’S DEMOGRAPHIC DETAILS.** (From this part onwards, the interviewer hands the tablet to the participant to answer the questions on his/her own) | | | | | |
|  | | | | | |
| **Q#** | **Question** | | **response** | | |
| 1.1 | How old were you on your last birthday? (*Age of the respondent*) | | _______ yrs. | | |
| 1.2 | What is your date of birth? (*DD*)*/MMM YYYY* | | ____ /____ /___ | | |
| **N.B- IF THE RESPONDENT IS YOUNGER THAN 18 YEARS OR OLDER THAN 24 YEARS ON THE DATE OF THE INTERVIEW, IMMEDIATELY STOP THE INTERVIEW AND THANK THE PERSON.** | | | | | |

| **Q#** | **Question** | **responses** | | | **code** | **Skip** |
| --- | --- | --- | --- | --- | --- | --- |
| 1.3 | How long have you lived here? | More than 6 months but less than a year. More than 1 year, but less than 5 years. More than 5 years. All my life. | | | 1 2 3 4 |  |
| 1.4 | What is your gender? | Female Male | | | 1 2 |  |
| 1.5 | What is your current marital status? (Marital status referring to legal, traditional or common-law) | | | | | |
|  | Single Not married or living together, but in a steady sexual relationship lasting more than 3 months Not married, but living with a partner/boyfriend/girlfriend  Married, living with spouse Married, but NOT living with spouse Divorced/Widowed Other (specify)________________________________________________ | | | | 1 2 3 4 5 6 7 |  |
|  | | | | | | |
| 1.6 | How would you describe your present employment situation? | | | | | |
|  | Housewife, homemaker, not looking for work Housewife, homemaker, looking for work  Unemployed, looking for work Unemployed, not looking for work Work in informal sector, looking for permanent work Work in informal sector, not looking for permanent work Sick/disabled and unable to work Student/pupil/learner Self-employed - full time (40 hours or more per week) Self-employed - part time (less than 40 hours per week) Employed part time (if none of the above) (less than 40 hours per week) Employed full time (40 hours or more per week)  Other________________________________________________ | | | | 1 2 3 4 5 6 7 8 9 10 11 12  13 |  |
| 1.7 | What is your highest level of education that you completed? | | | |  |  |
|  | No schooling Grade 1/Sub a/Class 1 Grade 2/Sub b/Class 2 Grade 3/Standard 1 Grade 4 /Standard 2 Grade 5 /Standard 3 Grade 6 /Standard 4 Grade 7/Standard 5 Grade 8 /Standard 6 Grade 9 /Standard 7 Grade 10/Standard 8 Grade 11/Standard 9 Grade 12/Standard 10/ Matric Further studies incomplete Certificate course completed after school (Grade 12/Matric) Diploma/Degree post school completed Further degree completed | | | | 1  2  3  4  5  6  7  8  9  10  11  12  13  14  15  16  17 |  |
| 1.8 | In the last 12 months, did you or other adults in your household have to reduce the amount of food? | | | Yes  No | 1  2 | If No go to question1.10 |
| 1.9 | In the last 12 months, were you ever hungry but did not eat food? | | | Yes  No | 1  2 | If No go to question1.10 |
|  | If any of your responses in 1.8 and 1.9 is Yes answer question 1.9a | | |  |  |  |
| 1.9a | What was the reason for reducing the amount of food or being hungry but did not eat? | | | No food  No money to buy food  Dieting  Other reason | 1  2  3  4 |  |
| 1.10 | Which of the following best describes your house? | | Brick house  Flat  Wendy house or back yard  (zozo)/Bungalow Shack (mkhukhu) | | 1  2  3  4 |  |
| 1.11 | Which of these do you have in your house?  ***You can choose more than one.*** | | Electricity  Radio  TV  Cellphone  Refrigerator  *Don’t remember*  *Refused / No response*  Bicycle  Motorcycle  Car / truck / van  Food Garden  Livestock (like chickens)  *Don’t remember*  *Refused / No response* | | 1  2  3  4  5  6  7  8  9  10  11  12  13  14 |  |
| 1.12 | Are you a member of any clubs or groups or societies (e.g. soccer club, church, youth club, savings club)?  (Tick only one) | | Yes, a member  Yes, an active member  No | | 1  2  3 |  |
| 1.13 | Do you receive a social grant (e.g. child support grant, disability grant, etc.) | | Yes  No | | 1  2 |  |
| 1.14 | What is your race? | | White  Black African  Indian/Asian  Coloured  Other, Specify __________ | | 1  2  3  4  5 |  |
| 1.15 | Where do you get most of your money to meet your basic monthly needs?  [Choose one] | | Social grant  Salary/Wages from employer  Business profit  Partner/spouse  Family  “Blesser”/ “Sugar Daddy”  Other, specify……………..……….. | | 1  2  3  4  5  6  7 |  |

| **SECTION 2 : KNOWLEDGE AND PERCEPTIONS OF HIV** | | | | | | | | |
| --- | --- | --- | --- | --- | --- | --- | --- | --- |
|  |  |  |  |  |  |  |  |  |

| **Q#** | **Question** | **responses** | | | **code** | | **Skip** |
| --- | --- | --- | --- | --- | --- | --- | --- |
| 2.1a | **Answer all the questions:** | | | | | | |
|  |  | | | Yes | | No |  |
|  | 1. To prevent HIV infection, a condom must be used for every round of sex | | | 1 | | 2 |  |
|  | 1. One can reduce the risk of HIV by having fewer sexual partners | | | 1 | | 2 |  |
|  | 1. Can a healthy-looking person have HIV? | | | 1 | | 2 |  |
|  | 1. Can AIDS be cured? | | | 1 | | 2 |  |
|  | 1. Can a person get HIV by sharing food with someone who is infected? | | | 1 | | 2 |  |
|  | 1. Can HIV be treated? | | | 1 | | 2 |  |
| 2.1b | Do you know how HIV can be prevented?  [**INSTRUCTION: MULTIPLE RESPONSES POSSIBLE]** | | | | | | |
|  | It can’t be prevented Using condoms Sticking to one sex partner Being faithful to one sex partner who is also faithful to you Reducing number of sex partners Abstaining from sex Avoiding contact with blood Using drugs to prevent the mother giving the child HIV Male circumcision  Microbicides (gel/ring inserted into the vagina to prevent HIV infection) Pre-exposure prophylaxis (PrEP) Post-exposure prophylaxis (PEP) Other (specify)_____________________________________ I don’t know | | | | | 1 2 3 4 5 6 7 8 9 10 11 12 13 14 |  |
| 2.2 | Is there a treatment for HIV? **[NOTE: Treatment being something to keep people healthy for a long time & not a cure for the disease]** | | Yes No Don't know | | | 1 2 3 | ***Go to 2.5 if you answered no or don’t know*** |
| 2.3 | What is that treatment? **[INSTRUCTION: MULTIPLE RESPONSES POSSIBLE]** | | | | | | |
|  | Antiretroviral drugs/treatment (ARVs/ART)  Other drugs, medicine, pills Traditional medicine Homeopathic treatment  Immune boosters Prayers Other (specify)__________________________ Don’t Know | | | | | 1 2 3 4 5 6 7 8 |  |
| 2.4 | How soon after testing HIV positive, can a person start treatment for HIV? **[INSTRUCTION: MULTIPLE RESPONSES POSSIBLE]** | | | | | | |
|  | Immediately When the CD4 count is low (below 500) Immediately if the person is a pregnant woman Immediately if the person has TB Whenever the person is ready to start treatment Other (specify)___________________________ Don’t Know | | | | | 1 2 3 4 5 6 7 |  |
| 2.6 | How long do people have to stay on that treatment for HIV? | | | | | | |
|  | For the rest of their lives. As long as they want. Until they feel better. Until they are cured,  Don’t know. Other (specify)____________________________________ | | | | | 1 2 3 4 5 6 |  |

| **Instruction:** the following questions are about people living with HIV  **INSTRUCTION : [** 1 = Strongly disagree  2 = Disagree  3 = Agree  4 = Strongly agree | | | | | |
| --- | --- | --- | --- | --- | --- |
|  | | **SD** | **D** | **A** | **SA** |
| 2.7.1 | I would stay friends with someone even if I found out he/she has HIV. | 1 | 2 | 3 | 4 |
| 2.7.2 | When you learn that you have HIV, your life is over. | 1 | 2 | 3 | 4 |
| 2.7.3 | I would be embarrassed to be seen with someone who everyone knows has HIV | 1 | 2 | 3 | 4 |
| 2.7.4 | Everyone who starts a new sexual relationship should get an HIV test before having sex. | 1 | 2 | 3 | 4 |
| 2.7.5 | To prevent getting HIV you have to use condoms every time you have sex with someone. | 1 | 2 | 3 | 4 |
| 2.7.6 | HIV is passed on most easily during the first 6 weeks after a person is infected. | 1 | 2 | 3 | 4 |
| 2.7.7 | A man who is circumcised does not need to use condoms to prevent getting HIV | 1 | 2 | 3 | 4 |

| **SECTION 3 : KNOWLEDGE, ATTITUDES, AND PERCEPTIONS OF TUBERCULOSIS (TB): This section is about TB** | | | | | | | | | | | | | | | | | | | | | | | | | | | | | | | | | | | |  |
| --- | --- | --- | --- | --- | --- | --- | --- | --- | --- | --- | --- | --- | --- | --- | --- | --- | --- | --- | --- | --- | --- | --- | --- | --- | --- | --- | --- | --- | --- | --- | --- | --- | --- | --- | --- | --- |
|  | |  | | |  | | |  |  | | | |  | | | | |  | | | | | | | | |  | | | | |  | | | |  |
| **Q#** | | **Statement** | | | | | | | | | | | | | | | | | | | | | | | | |  | | | | **Skip** | | | | |  |
| 3.1 | | Do you know what Tuberculosis (TB) is? | | | | | | | | | | | | | | | | | | | | | | Yes  No | | | 1  2 | | | |  | | | | |  |
| **Instruction:** This section will ask about tuberculosis (TB). Remember that there are no right or wrong answers  1 = Agree (A)  2 = Disagree (D)  3 = Don't know (DK) | | | | | | | | | | | | | | | | | | | | | | | | | | | | | | | | | | | |  |
| **Q#** | | **Statement** | | | | | | | | | | | | | | | | | | | | | | | | | **A** | | **D** | | | | | **DK** | |  |
| 3.1.1 | | Anybody can get TB | | | | | | | | | | | | | | | | | | | | | | | | | 1 | | 2 | | | | | 3 | |  |
| 3.1.2 | | People living with HIV are more likely to get TB | | | | | | | | | | | | | | | | | | | | | | | | | 1 | | 2 | | | | | 3 | |  |
| 3.1.3 | | People that are HIV negative can get TB | | | | | | | | | | | | | | | | | | | | | | | | | 1 | | 2 | | | | | 3 | |  |
|  | |  | | | | | | | | | | | | | | | |  | | | | | | | | |  | | | | |  | | | |  |
| **Q#** | | **Question** | | | | | | | | | | | **Responses** | | | | | | | | | | | | | | **code** | | | | | **skip** | | | |  |
| 3.2 | | How can a person get TB?  [**INSTRUCTION: MULTIPLE RESPONSES POSSIBLE** | | | | | | | | | | | | | | | | | | | | | | | | | | | | | | | | | |  |
|  |  | Through handshakes Through the air when a person with TB coughs or sneezes Through sharing dishes Through eating from the same plate Through touching items in public places (doorknobs, handles in transportation etc.) Smoking Other (specify)______________________________ Don’t Know | | | | | | | | | | | | | | | | | | | | | | | | | 1 2 3 4 5 6 55 96 | | | | |  | | | |  |
| 3.3 | | What are the signs and symptoms of TB? | | | | | | | | | | | | | | | | | | | | | | | | | | | | | | | | | |  |
|  |  | [MULTIPLE RESPONSES ALLOWED] | | | | | | | | | | | Cough that lasts longer than 3 weeks Coughing up blood Loss of appetite Fever Night sweats Unintentional weight loss Other (specify)_______________________ Don’t Know | | | | | | | | | | | | | | 1 2 3 4 5 6 55 96 | | | | |  | | | |  |
| 3.4 | | What is the treatment for TB? | | | | | | | | | | Drugs, medicine, pills There is no treatment Traditional medicine Don’t know | | | | | | | | | | | | | | | 1 2 3 96 | | | | | **if you answered no treatment or drugs/medicine/pills or don’t know Go to Q3.6** | | |  |  |
| 3.5 | | How long does someone have to take the drugs to cure TB? | | | | | | | | | | | | | | | | | | | | | | | | | | | | | | | | | |  |
|  |  | **[ONE RESPONSE ONLY]** | | | | | | | | | | | One month or less Two to five months Six months or longer Other (specify)______________________ Don’t Know | | | | | | | | | | | | | | 1 2 3 55 96 | | | | |  | | | |  |
| 3.6 | | Are people with TB always HIV positive? | | | | | | | | | | | | | | | | | | | | | | | | | | | | | | | | | |  |
|  |  |  | | | | | | | | | | | | | | | | | | | Yes No Don't know | | | | | | 1 2 96 | | | | |  | | | |  |
| 3.7 | | Is it possible to cure TB in people with HIV? | | | | | | | | | | | | | | | | | | | | | | | | | | | | | | | | | |  |
|  |  |  | | | | | | | | | | | Yes No Don't know | | | | | | | | | | | | | | 1 2 96 | | | | |  | | | |  |
| **Instruction**: The following question is about how a person who has TB is usually regarded/treated in your community?  **INSTRUCTION : CHECK ONE ANSWER** 1 = Strongly disagree (SD)  2 = disagree (D)  3 = Agree (A)  4 = Strongly Agree (SA) | | | | | | | | | | | | | | | | | | | | | | | | | | | | | | | | | | | |  |
|  | | | | | | | | | | | | | | | | | | | | | | | **SD** | | | | **D** | | | | **A** | **SA** | | | |  |
| 3.8 | | | Most people reject him or her | | | | | | | | | | | | | | | | | | | | 1 | | | | 2 | | | | 3 | 4 | | | |  |
| 3.9 | | | Most people are friendly but they generally try to avoid him or her | | | | | | | | | | | | | | | | | | | | 1 | | | | 2 | | | | 3 | 4 | | | |  |
| 3.10 | | | The community mostly supports him or her | | | | | | | | | | | | | | | | | | | | 1 | | | | 2 | | | | 3 | 4 | | | |  |
|  | | |  | | |  | | | | |  |  | | | | |  | | | | | | | | | |  | | | | | |  | |  | |
| **Q#** | | | **Question** | | | | | | | | | | | | | **responses** | | | | | | | **code** | | | | | **skip** | | | | | | | |  |
| 3.11 | | | Has anyone in your household ever had TB? | | | | | | | | | | | | | | | | | | | | | | | | | | | | | | | | |  |
|  | | |  | | | | | | | | | | | | | Yes No No response | | | | | | | | | | 1 2 3 | |  | | | | | | | |  |
| 3.11a | | | Have you ever had a TB test? | | | | | | | | | | | | | | | | | | | | | | | | | | | | | | | | |  |
|  |  |  |  | | | | | | | | | | | | | Yes No No response | | | | | | | | | | 1 2 3 | | **If your answer is no, or no response please go to 4.1** | | | | | | | |  |
| 3.12 | | | How long ago did you have a TB Test | | | | | | | | | | | | | | | | | | | | | | | | | | | | | | | | |  |
|  |  |  |  | | | | | | | | | | | | | 0 to 3 Months  4 to 6 Months  7 to 11 Months  Less than a year ago  Between 1-2 years ago  Between 2-3 years ago  Three or more years ago | | | | | | | | | | 1 2 3  4  5  6  7 | |  | | | | | | | |  |
| 3.13 | | | Where did you get your TB test done? | | | | | | | | | | | | | At a clinic/hospital  A community worker  A traditional healer  Pharmacy  Other (specify)___________ | | | | | | | | | | 1  2  3  4  55 | |  | | | | | | | |  |
| 3.14 | | | What specimen did they collect to test for TB? | | | | | | | | | | | | | Blood  Sputum  Other (specify)___________ | | | | | | | | | | 1  2  55 | |  | | | | | | | |  |
| **SECTION 4 : ALCOHOL USE** | | | | | | | | | | | | | | | | | | | | | | | | | | | | | | | | | | |  |  |
|  | | | |  | | |  | | |  | | | |  |  | | | |  |  | |  | | | | | | | | | | | | |  |  |
| **Instruction : The following questions are about alcohol use** | | | | | | | | | | | | | | | | | | | | | | | | | | | | | | | | | | |  |  |
| **Q#** | **Question** | | | | | | | | | | | **responses** | | | | | | | | | | | | | **code** | | | | | **skip** | | | | |  |  |
| 4.1 | How often do you use drugs (dagga, benzene, tik, cocaine, nyaope, wunga, mandrax, and heroin) or something else to make you high? | | | | | | | | | | | Never  Almost every time  Often but not every time  It has happened every week | | | | | | | | | | | | | 0  1  2  3 | | | | |  | | | | |  |  |
| 4.2 | How often do you usually drink alcohol? | | | | | | | | | | | I have never drank alcohol every week  3 times a month less than once a month less than once in 6 months | | | | | | | | | | | | | 0 1 2 3 4 | | | | | If your answer is never drank alcohol go to section 5 | | | | |  |  |
| 4.3 | How many alcoholic drinks do you have daily? | | | | | | | | | | | 1 or 2  3 or 4  5 or 6  7, 8, or 9  10 or more | | | | | | | | | | | | | 0 1 2 3 4 | | | | |  | | | | |  |  |
| 4.4 | How often do you have six or more drinks on one occasion? | | | | | | | | | | | Never  Less than monthly  Monthly  Weekly  Daily or almost daily | | | | | | | | | | | | | 0 1 2 3 4 | | | | |  | | | | |  |  |
| 4.5 | How often during the last year have you failed to do what was normally expected from you because of drinking? | | | | | | | | | | | Never  Less than monthly  Monthly  Weekly  Daily or almost daily | | | | | | | | | | | | | 0 1 2 3 4 | | | | |  | | | | |  |  |
| 4.6 | How often during the last year have you needed a first drink in the morning to get yourself going after a heavy drinking session? | | | | | | | | | | | Never  Less than monthly  Monthly  Weekly  Daily or almost daily | | | | | | | | | | | | | 0 1 2 3 4 | | | | |  | | | | |  |  |
| 4.7 | How often during the last year have you had a feeling of guilt or remorse after drinking? | | | | | | | | | | | Never  Less than monthly  Monthly  Weekly  Daily or almost daily | | | | | | | | | | | | | 0 1 2 3 4 | | | | |  | | | | |  |  |
| 4.8 | How often during the last year have you been unable to remember what happened the night before because you had been drinking? | | | | | | | | | | | Never  Less than monthly  Monthly  Weekly  Daily or almost daily | | | | | | | | | | | | | 0 1 2 3 4 | | | | |  | | | | |  |  |
| 4.9 | Have you or someone else been injured as a result of your drinking? | | | | | | | | | | | No  Yes, but not in the last year  Yes, during the last year | | | | | | | | | | | | | 0 1 2 | | | | |  | | | | |  |  |
| 4.10 | Has a relative or friend or a doctor or another health worker been concerned about your drinking or suggested you cut down? | | | | | | | | | | | No  Yes, but not in the last year  Yes, during the last year | | | | | | | | | | | | | 0 1  2 | | | | |  | | | | |  |  |

| **SECTION 5 : SEXUAL HISTORY** | | | | | | | | |
| --- | --- | --- | --- | --- | --- | --- | --- | --- |
|  |  |  |  |  |  |  |  |  |
| **INSTRUCTION:** This section asks you sensitive questions on sex and other sex-related matters. Please remember that your name will not be recorded anywhere in this questionnaire and the information you give will be kept confidential. | | | | | | | | |

| **Q#** | **Question** | | **Responses** | | **code** | | | **skip** |
| --- | --- | --- | --- | --- | --- | --- | --- | --- |
| 5.1 | Have you ever had sexual intercourse?  [*For the purposes of this survey, “sexual intercourse” is defined as penetrative vaginal/anal sex*.] | | | Yes No | | 1 2 | | **If your answer is no, go to question 5.8** |
| 5.2 | Why have you not had sex yet? | | | | | | | |
|  |  | Not ready I am too young Not interested Avoiding pregnancy Avoiding STIs, including HIV Religious grounds Cultural grounds Don't have a partner I do not have access to protection (such as condoms)  No response  Other (specify)_________________________ | | | | 1 2 3 4 5 6 7 8 9  10 11 | |  |
| 5.3 | How old were you when you had sex for the first time? [**record age in years**] | _________ yrs. Don't Remember Age | | | | 1 | |  |
| 5.4 | Who did you have sex with the first time you had it? | | | | | | | |
|  |  | A boyfriend or girlfriend A friend Husband/wife Someone I knew for a day or less Family member/relative Someone I knew but he was not a friend Someone I didn't know | | | | | 1 2 3 4 5 6 7 |  |
| 5.5 | How would you describe your first sexual experience? | | | | | | | |
|  |  | I was willing I was persuaded I was tricked I was forced I was raped | | | | | 1 2 3 4 5 |  |
| 5.6 | Did you use a condom the first time you had sex? | Yes No Cannot remember | | | | | 1 2 3 |  |

|  | |  | |  |  | | |  | | |  | |  | |  | | |  |  |
| --- | --- | --- | --- | --- | --- | --- | --- | --- | --- | --- | --- | --- | --- | --- | --- | --- | --- | --- | --- |
| **Q#** | **Question** | | | | | | | | | | | **responses** | | | | **code** | **skip** | | |
| 5.7 | In the last 12 months have you had sex? | | | | | | | | | | | Yes No No response | | | | 1 2 3 | **If your answer is No or no response go to 5.4** | | |
| 5.8 | How many sexual partners did you have in your life? | | | | | | | | | | | | | | | _____ |  | | |
| 5.9 | In the last 12 months how many sexual partners did you have? | | | | | | | | | | | | | | | _____ |  | | |
| 5.10 | **For women**: Have you ever had sex with anybody because you expected or hoped they would give you money or something else?  **For men**: Have you ever had sex with anybody because the person expected or hoped you would give her or you promised her money or something else? | | | | | | | | I got money  I got gifts  I got transport  I got food or drink  I had a had a good time No | | | | | | | 1 2 3  4  5  6 |  | | |
|  |  | |  |  | | | | |  | | |  | |  | |  |  | | |
| **REDCAP INSTRUCTION** : Ask these questions If more than one partner has been recorded above **Instruction** : The following questions about your sexual activity in general | | | | | | | | | | | | | | | | | | | |
| 5.11 | Did any of these relationships mentioned above overlap with each other (occur at the same time)? | | | | | | | | | | | Yes No No response | | | | 1 2 3 |  | | |
| 5.12 | Do you have two or more sexual partners at the moment? | | | | | | | | | | | Yes No No response | | | | 1 2 3 |  | | |
| 5.13 | In the last 3 months how many different sexual partners did you have ? | | | | | | | | | | | | | | | _____ |  | | |
| 5.14 | In the last 3 months have you used a condom with any of your partners? | | | | | | | | | | | Yes No | | | | 1 2 |  | | |
| 5.15 | What is your relationship with your most recent sexual partner? | | | | | | Husband / Wife Live-in partner Girlfriend / Boyfriend Not living with me Casual partner Someone whom I paid/received payment from for sex Other(specify) _________________________ | | | | | | | | | 1 2 3 4 5  6 |  | | |
| 5.16 | Is this most recent sexual partner a male or a female? | | | | | | | | | Male Female | | | | | | 1 2 |  | | |
| 5.17 | Where does this partner live? | | | | | | | | | In same area In another area In the same household | | | | | | 1 2 3 |  | | |
| 5.18 | What is the age of this person?  [**record age in years**] | | | | | | | | | _________ yrs. Don’t know | | | | | | 88 |  | | |
| 5.19 | Did you use a condom during your last sexual intercourse? | | | | | | | | | Yes No | | | | | | 1 2 | **if your answer is No go to 5.21** | | |
| 5.20 | If you used a condom, what were your reasons for doing so? | | | | | | | | | | | | | | | | | | |
|  | Concern about HIV infection People are urged to use condoms Want to prevent STIs  Want to prevent pregnancy I am or my partner is on ARVs Partner insisted on condom use Other: please specify___________________________________________________ | | | | | | | | | | | | | | | 1 2 3 4 5 6 7 | **Continue to Q5.22** | | |
| 5.21 | If you DID NOT use a condom, what were your reasons for doing so? | | | | | | | | | | | | | | | | | | |
|  | Did not have a condom Partner objected Used other contraceptive Don’t like them Didn’t think it was necessary I am married I am faithful/trust them I was drunk/high  I did not know how to use the condom  I want a baby Other(specify)________________________________________________ | | | | | | | | | | | | | | | 1 2 3 4 5 6 7 8  9 10  11 |  | | |
| 5.22 | Is it easy to get a **male** condom if you need one? | | | | | | | | | | | Yes No No response | | | | 1 2 3 |  | | |
| 5.23 | Is it easy to get a **female** condom if you need one? | | | | | Yes No No response | | | | | | | | | | 1 2 3 |  | | |
| **INSTRUCTION for tablet programming: The next question (Q5.19) is for sexually active respondents who never used a condom** | | | | | | | | | | | | | | | | | | | |
| 5.24 | Where do you normally obtain your condoms? | | | | | | | | | | | | | | | | | | |
|  | [DO NOT READ OUT OPTIONS. MULTIPLE RESPONSES POSSIBLE] | | | | | clinic or hospital Pharmacy/chemist Shop/supermarket/café/spaza shop garage/ petrol station shebeen/tavern/pub from my friends From My partner  From my parent/s  Not Applicable  Community health/HIV worker  Other(specify)____________________________ | | | | | | | | | | 1 2 3 4 5 6 7 8  9  10  11 |  | | |

| **SECTION 6 : HIV COUNSELLING AND TESTING** | | | | | | | | |
| --- | --- | --- | --- | --- | --- | --- | --- | --- |
|  |  |  |  |  |  |  |  |  |
| **Instruction:** The following questions are about testing for HIV. Please remember that your name will not be recorded anywhere in this questionnaire and the information you give will be kept confidential. | | | | | | | | |

| **Q#** | **Question** | | | | | **responses** | | **code** | | | **skip** |
| --- | --- | --- | --- | --- | --- | --- | --- | --- | --- | --- | --- |
| 6.1 | Have you ever had an HIV test? | | | | | | Yes No | | 1 2 | |  |
| 6.2 | Do you know of a place nearby where you can get an HIV test? | | | | | | Yes No | | 1 2 | | **Skip to 6.5** |
| 6.3 | How long ago did you have an HIV test/retest? | | | | | | | | | | |
|  |  | | | 0 to 3 months 4 to 6 months 7 to 11 months Less than a year ago Between 1-2 years ago Between 2-3 years ago Three or more years ago | | | | | 1 2 3 4 5 6 7 | |  |
| 6.4 | Where did you get your most recent HIV test? | | | Public hospital Private hospital Public clinic or doctor Private clinic or doctor Local NGO/ community based organization Workplace Community testing event Other______________________________ | | | | | 1 2 3 4 5 6 7 8 | |  |
| 6.5 | Where would you prefer to go for an HIV test? | | | Public hospital Private hospital Public clinic or doctor Private clinic or doctor  NGO/ community based organization Workplace Community testing event  Home Other______________________________ | | | | | 1 2 3 4 5 6 7 8  9 | | Skip to 6.18 |
| **Instruction**: Please note that you do not need to disclose your Hiv status . I am only interested in knowing whether you have been told/informed of the result of the test. | | | | | | | | | | | |
|  |  | | | | | | | | | | |
| 6.6 | During your most recent HIV test, were you given information before the HIV test? | | | | | Yes No | | | 1 2 | |  |
| 6.7 | During your most recent HIV test, were you given information individually or in a group? | | | | | individually  as a couple | | | 1 2 | |  |
| 6.8 | Have you been told/informed of the result of your most recent test? | | | | | Yes No | | | 1 2 | |  |
| 6.9 | During your most recent HIV test, did you have counselling after the HIV test? | | | | | Yes No | | | 1 2 | |  |
| 6.10 | What were your reasons for going for your last HIV test? | | | | | | | | | | |
|  | **INSTUCTION**: **MULTIPLE RESPONSES POSSIBLE** | | I wanted to know my HIV status My partner asked me to go for testing I wanted to start a new sexual relationship I wanted to get married I applied for an insurance policy I applied for a loan My employer requested it I was feeling sick I was instructed by a health worker (nurse/doctor) I was pregnant Workplace campaign Other | | | | | | 1 2 3 4 5 6 7 8 9 10 11 12 | |  |
| 6.11 | You indicated that you were previously tested for HIV. Are you willing to share the last HIV test result you received? | | | | | Yes No Never received | | | 1 2 3 | | **If your answer is No or never received go to Question 6.16** |
| 6.12a | What was the result of that HIV test? | | | | | Positive Negative Indeterminate | | | 1 2 3 | | **If your answer is Negative or indeterminate Go to Q6.15** |
| 6.12b | What was the month and year of your first HIV positive test? **IF “DON’T KNOW” MONTH, THEN RECORD ‘88’ IF “DON’T KNOW” YEAR, THEN RECORD ‘8888’** | | | | | **MONTH** | | | ___ | |  |
|  |  |  |  |  |  | **YEAR** | | | ___ | |  |
| 6.13 | Are you currently taking ARVs, that is, antiretroviral medications to treat HIV? | | | | | Yes No | | | 1 2 | | **If your answer is No Go to Q6.16** |
| 6.14 | How long have you been taking daily ARVs?  **RECORD THE ANSWER IN MONTHS IF LESS THAN ONE YEAR. RECORD '00' IF LESS THAN ONE MONTH.** | | | | | Number of months | | | ___ | |  |
|  |  |  |  |  |  | Number of years | | | ____ | |  |
| 6.15 | Did you tell your main partner about your HIV status? | | | | | Yes No Don’t know | | | 1 2 3 | |  |
| 6.16 | Have you told other sexual partners about your test result during the last 12 months? | | | | | Yes No No Partner | | | 1 2 3 | |  |
| 6.17 | Have you ever taken an HIV test with any of your sex partners where you both received the test results together? | | | | | Yes No | | | 1 2 | |  |
|  | | | | | | | | | | | |
| 6.18 | What are your reasons for not going for an HIV test? | | | | | | | | | | |
|  | **INSTUCTION**: **MULTIPLE RESPONSES POSSIBLE** | Do not know where to get tested Do not think that I have HIV Not at risk for HIV Trust partner Afraid to find out that he/she might be HIV positive Not ready to have an HIV test Concerned about confidentiality Concerned about stigma, discrimination, or rejection Concerned about losing my job Concerned about the standard of service Haven’t got around to do it  I am concerned about the costs  Other__________________________________________ | | | | | | | | 1 2 3 4 5 6 7 8  9 10 11 12  13 |  |
| 6.19 | How many of your friends do you think have been tested for HIV? | | | | | | | | | | |
|  | **INSTUCTION**: **MULTIPLE RESPONSES POSSIBLE** | | | | 1 out of 10 2 out of 10 3 out of 10 4 out of 10 5 out of 10 6 out of 10 7 out of 10 8 out of 10 9 out of 10 10 out of 10 | | | | | 1 2 3 4 5 6 7 8 9 10 |  |

| **Instruction:** How much do you agree with the following statements about HIV testing in your community? There are no right or wrong answers, only people’s opinions. We would like to know which opinions you agree or disagree with.  **INSTRUCTION** : Select one of the following responses for each question  **1 = Strongly disagree (SD)  2 = Disagree (D)   3 = Agree (A)  4 = Strongly Agree (SA)** |
| --- |

|  | | **SD** | **D** | **A** | **SA** |
| --- | --- | --- | --- | --- | --- |
| 6.20a | People in my community who test positive feel free to tell their friends and family that they have HIV | 1 | 2 | 3 | 4 |
| 6.20b | People in my community do not test for HIV because they are scared it would change their lifestyle (i.e. use condoms, reduce number of partners, stop drinking alcohol and smoking) | 1 | 2 | 3 | 4 |
| 6.20c | People in my community do not test for HIV because they are scared that they are already HIV positive | 1 | 2 | 3 | 4 |
| 6.20d | People in my community are scared to test for HIV because they think that if they test HIV positive, they can never have a relationship again | 1 | 2 | 3 | 4 |
| 6.20e | People in my community who test for HIV are regarded as strong and responsible | 1 | 2 | 3 | 4 |
| 6.20f | People in my community are scared to test for HIV because if they test positive their family and friends will reject them | 1 | 2 | 3 | 4 |

| **SECTION 7 : HIV RISK PERCEPTION, ATTITUDES AND SOCIAL NORMS FOR MULTIPLE PARTNERS AND CONDOMS** | | | | | | | | |
| --- | --- | --- | --- | --- | --- | --- | --- | --- |
|  |  |  |  |  |  |  |  |  |
| **Instruction** : This section is about your thoughts about yourself in relation to HIV infection | | | | | | | | |

| **Q#** | **Question** | | | | **responses** | | **code** | **skip** |
| --- | --- | --- | --- | --- | --- | --- | --- | --- |
| 7.1 | Choose the statement that best describes yourself in terms of HIV infection | | | | | | | |
|  |  | | | | | |  |  |
| A | I am definitely going to get infected with HIV | | | | | | 1 |  |
| B | I am probably going to get infected with HIV | | | | | | 2 |  |
| C | I probably won’t get infected with HIV | | | | | | 3 |  |
| D | I definitely will not get infected with HIV | | | | | | 4 |  |
|  |  |  |  |  |  |  |  |  |
| 7.2 | What are your reasons for believing that you probably or definitely will NOT get infected with HIV?  (**Respondents who answered 3 or 4 in Q7**.1)  **INSTUCTION**: **MULTIPLE RESPONSES POSSIBLE** | | Never had sex before Abstain/withholding from sex Faithful to partner Trust my partner Use condoms Know my HIV status Know the HIV status of my partner Do not have sex with sex workers/prostitutes Protected by ancestors Protected by God  I am not at risk of HIV Other | | | | 1 2 3 4 5 6 7 8 9 10 11 12 | **skip Q7.3 and Proceed to Q7.4** |
| 7.3 | What are your reasons for believing that you probably or definitely will get infected with HIV?  (**Respondents who answered 1 or 2 in Q7.1**) | | | | | | | |
|  | **INSTUCTION**: **MULTIPLE RESPONSES POSSIBLE** | | Sexually active Had many sexual partners Don't use condoms Don't always use condoms Don't trust his/her partner I am sick My partner is sick My partner died of AIDS Had an accident/cuts my partner is HIV positive Other(specify)______________________________ | | | | 1 2 3 4 5 6 7 8 9 10 11 |  |
| 7.4 | Would you be willing to test yourself for HIV if you were to be given an HIV self-test kit? | | | | Yes No Don’t know | | 1 2 3 |  |
|  |  |  |  |  |  |  |  |  |

| **Q#** | **Question** | | | | | | | | **responses** | | | | | | | |
| --- | --- | --- | --- | --- | --- | --- | --- | --- | --- | --- | --- | --- | --- | --- | --- | --- |
| 7.5 | **Instruction :** The following questions are about condoms and relationships 1 = Strongly disagree (SD) 2 = Disagree (D) 3 = Agree (A) 4 = Strongly agree (SA) | | | | | | | | | | | | | | | |
|  |  | | | | | | | | **SD** | | **D** | | | **A** | | **SA** |
| a | **(Men)** Men are afraid that their wife/girlfriend will turn them down if they suggest using a condom. | | | | | | | | 1 | | 2 | | | 3 | | 4 |
| b | **(Women)** Women are afraid that her husband/boyfriend will turn her down if she suggests using a condom. | | | | | | | | 1 | | 2 | | | 3 | | 4 |
| c | If someone ever has trouble putting on a condom, they will be embarrassed to try to use a condom again. | | | | | | | | 1 | | 2 | | | 3 | | 4 |
| d | Women who carry condoms are looking out for their own health. | | | | | | | | 1 | | 2 | | | 3 | | 4 |
| e | Using a condom will make your partner think you don’t trust him/her. | | | | | | | | 1 | | 2 | | | 3 | | 4 |
| f | When you use a condom, you can’t get enough pleasure. | | | | | | | | 1 | | 2 | | | 3 | | 4 |
| g | If you have good communication with your partner, you can be sexually satisfied with one person. | | | | | | | | 1 | | 2 | | | 3 | | 4 |
| h | I need someone else to fill the gap in case I ever break up with my main partner. | | | | | | | | 1 | | 2 | | | 3 | | 4 |
| I | Now and then, I go to someone else besides my main partner because the sex is so good. | | | | | | | | 1 | | 2 | | | 3 | | 4 |
| j | It's ok to have sex with others as long as your main partner does not find out. | | | | | | | | 1 | | 2 | | | 3 | | 4 |
| k | Most of my friends brag about how many people they are having sex with. | | | | | | | | 1 | | 2 | | | 3 | | 4 |
| l | Condoms are a lot of fun | | | | | | | | 1 | | 2 | | | 3 | | 4 |
| **Q#** | | **Question** | | | | **responses** | | | **code** | | | | | **skip** | | |
| 7.6 | | On a scale of 1 to 10 How many men in your community do you think have sex with more than one woman? | | | | | | | _____ | | | | |  | | |
| 7.7 | | Do you think that this practice of men having sex with more than one woman is acceptable or not? | | | | | Acceptable Not acceptable Don’t know | | 1 2 3 | | | | |  | | |
| 7.8 | | On a scale of 1 to 10 how many women in your community do you think have sex with more than one man? | | | | | | | _____ | | | | |  | | |
| 7.9 | | Personally, do you think that this practice of women having sex with more than one man is acceptable or not? | | | | | Acceptable Not acceptable Don’t know | | 1 2 3 | | | | |  | | |
|  | |  |  |  |  | |  | | | | |  |  | |  | |
| **Q#** | | **Question** | | | | | **responses** | | | | | | | | | |
| 7.10 | | **Instruction:** How much do you agree with the following statements about yourself?  1 = Strongly disagree (SD) 2 = Disagree (D) 3 = Agree (A) 4 = Strongly agree (SA) | | | | | | | | | | | | | | |
|  | |  | | | | | | **SD** | | **D** | | | **A** | | | **SA** |
| a | | I can use a condom even when I have too much to drink. | | | | | | 1 | | 2 | | | 3 | | | 4 |
| b | | I can refuse to have sex if someone I like refuses to use a condom. | | | | | | 1 | | 2 | | | 3 | | | 4 |
| c | | I can buy condoms without feeling embarrassed. | | | | | | 1 | | 2 | | | 3 | | | 4 |
| d | | **[MEN)** I am confident that I can put a condom on correctly.  **[WOMEN]** I am confident that I can correctly put a condom on a man when having sex with him | | | | | | 1 | | 2 | | | 3 | | | 4 |
| e | | I never know what to say when my partner and I need to talk about condoms or other protection | | | | | | 1 | | 2 | | | 3 | | | 4 |
| f | | I always feel really uncomfortable when I buy condoms | | | | | | 1 | | 2 | | | 3 | | | 4 |

| **SECTION 8 : MALE CIRCUMCISION** | | | | | | | | |
| --- | --- | --- | --- | --- | --- | --- | --- | --- |
|  |  |  |  |  |  |  |  |  |
| **Instruction**: The following questions are about male circumcision about yourself (to men)/ about your sexual partner (to women) | | | | | | | | |

| **Q#** | **Question** | | **Responses** | **code** | **skip** |
| --- | --- | --- | --- | --- | --- |
| 8.1 | **(Men)**: Are you circumcised? (**Women)**: Is your partner circumcised? | | Yes No No response | 1 2 3 | **If your answer is Yes skip 8.3 If your answer is No go to 8.3. If female go to section 9** |
| 8.2 | Why were you circumcised? | | | | |
|  | **INSTUCTION**: **MULTIPLE RESPONSES POSSIBLE** | Heard in the media  My parents decided Cultural tradition/religion To prevent HIV  To prevent STIs Other (specify)______________________  Refused to answer | | 1 2 3 4 5 6 7 | Skip to 9.1 |
| 8.3 | Why have you not been circumcised? | | | | |
|  | **INSTUCTION**: **MULTIPLE RESPONSES POSSIBLE** | One can become infertile Reduces sexual pleasure Unsure about safety of procedure None of my friends are circumcised My friends will make fun of me My partner opposes Not part of my culture/tradition I am afraid of the pain I do not want to abstain from sex for long (six weeks) Other (Specify)__________________________________ Refused to answer | | 1 2 3 4 5 6 7 8 9 10 11 |  |
| 8.4 | In the next 12 months do you plan to get circumcised? | | Definitely will  Probably will  Probably will not  Definitely will not Unsure | 1 2 3 4 5 |  |

| **SECTION 9 : PRE-EXPOSURE PROPHYLAXIS** | | | | | | | | |
| --- | --- | --- | --- | --- | --- | --- | --- | --- |
|  |  |  |  |  |  |  |  |  |
| **Instruction:** The following questions are about the use of medication to help prevent HIV infections. Remember there are no right or wrong answers and anything you say will be kept confidential. If you do not want to answer a question, you may skip to the next question. | | | | | | | | |

| **Q#** | **Question** | | **Responses** | **code** | **skip** |
| --- | --- | --- | --- | --- | --- |
| 9.1 | Have you ever heard about Pre-exposure prophylaxis, which is also called PrEP? | | Yes No | 1 2 |  |
| 9.2 | Have you ever heard about HIV medication that is taken to help prevent becoming infected with HIV? | | Yes No | 1 2 |  |
| **INFO:** PrEP stands for Pre-Exposure Prophylaxis. It is a pill that is taken every day by people who are HIV negative to help prevent them from becoming HIV positive. | | | | | |
| 9.3 | Would you be interested in using PrEP? | | Yes No Not sure | 1 2 3 | **If your answer is No go to Q9.5 If your answer is Not sure go to Q9.6** |
| 9.4 | How would you prefer to get access to PrEP? [**Choose the two most important**] | | Private doctor Hospital Clinic  Pharmacy/Chemist School Community health workers Non-governmental organization (NGO) Traditional healer Mobile clinic Family member Other(specify)___________________ | 1 2 3 4 5 6 7 8 9 10 11 | **if any two are picked , then go to Q9.6** |
| 9.5 | I would not use PrEP because: [**Choose the two most important**] | | | | |
|  |  | Possible side effects I don’t know enough about it I would not want my family or friends to know that I’m using it If I use it I would be more sexually active  I don’t want to drink a pill every day If I use it I will have riskier sex, like not using a condom Other(specify)_______________ | | 1 2 3 4 5 6 7 |  |
| 9.6 | What are the three most important things you would like to know about PrEP to help you decide if you would want to use it?  [**Choose the three most important**] | | | | |
|  |  | Side effects Place where I can get the pill Person who gives the medicine Duration of taking the pill How well this pill works How often I have to take it (once a day vs. before sex acts) Cost of the pill How it is taken Other (specify) _________________________________ | | 1 2 3 4 5 6 7 8 9 |  |
| **INFO:** We would like you to advise us on how best to give information about PrEP to you and your friends. | | | | | |
| 9.6 | What would be the best way of telling young people in your community about PrEP? [**Choose the three most important**] | | | | |
|  |  | Newspaper  Billboards advertisements  School visits TV advertisements  Brochures handed out at health facilities  Social media like Facebook and WhatsApp Other(specify)__________________________________ | | 1 2 3 4 5 6 7 |  |

| **SECTION 10 : MEDIA, COMMUNICATION AND NORMS** | | | | | | | | |
| --- | --- | --- | --- | --- | --- | --- | --- | --- |
|  |  |  |  |  |  |  |  |  |
| **Instruction**: The following questions are about sources of information and what you think of them | | | | | | | | |

| **Q#** | **Question** | | | **responses** | | | | |
| --- | --- | --- | --- | --- | --- | --- | --- | --- |
| 10.1 | **:** How often do you do the following?   **1 = Never (N)  2 = Once in a while/ rarely (O)  3 = Once a week (OW)  4 = 2-6 days a week (2DW)  5 = Every day of the week (EW)** | | | | | | | |
|  |  | | | **N** | **O** | **OW** | **2DW** | **EW** |
| a | Listen to the radio | | | 1 | 2 | 3 | 4 | 5 |
| b | Watch television | | | 1 | 2 | 3 | 4 | 5 |
| c | Read a print magazine | | | 1 | 2 | 3 | 4 | 5 |
| d | Read a print newspaper | | | 1 | 2 | 3 | 4 | 5 |
| e | Use the internet to go onto news sites | | | 1 | 2 | 3 | 4 | 5 |
| f | Use cell phone or computer or Tablet to go onto Facebook | | | 1 | 2 | 3 | 4 | 5 |
| g | Use cell phone or computer or Tablet to go onto Twitter | | | 1 | 2 | 3 | 4 | 5 |
| h | Use the cell phone or computer or Tablet to go onto HIV or other health related websites | | | 1 | 2 | 3 | 4 | 5 |
| I | Use cell phone or the internet to go onto Instagram | | | 1 | 2 | 3 | 4 | 5 |
| j | Use a cellphone to go onto WhatsApp | | | 1 | 2 | 3 | 4 | 5 |
|  |  |  |  |  |  |  |  |  |
| **Q#** | **Question** | | | **responses** | | | | |
| **Instruction:** The following questions are about your preferred media channels or pages **REDCap Instruction: ONLY ASK IF ANSWER ABOVE IS 2, 3, 4 OR 5** | | | | | | | | |
| 10.2 | Which radio stations do you listen to the most?  **[Allow list up to 3 stations]** | | | 1 =________________ 2 =________________ 3 _________________ | | | | |
| 10.3 | Which TV stations do you watch the most?  **[Allow list up to 3 stations]** | | | 1 =________________ 2 =________________ 3 _________________ | | | | |
| 10.4 | Which websites do you visit most often? **[Allow list up to 3 websites]** | | | 1 =________________ 2 =________________ 3 _________________ | | | | |
| 10.5 | Which Facebook pages or groups do you follow most often? **[Allow list up to 3 pages or groups]** | | | 1 =________________ 2 =________________ 3 _________________ | | | | |
| 10.6 | How do you prefer to get messages about HIV or TB prevention, testing and treatment? Tick all that apply | | | Radio  Newspaper  Short message service (sms)  WhatsApp Social networking sites e.g. Facebook, twitter,  Television  Print magazines | | | 1  2  3  4  5  6  7 | |

You have come to the end of our questionnaire. Thank you very much for participating in this survey. We really appreciate you spending your time on this survey. FINISH

.
