# Supplementary material for: Study on knowledge about associated factors of Tuberculosis (TB) and TB/HIV co-infection among young adults in two districts of South Africa
Source: PLoS One. 2019 Jun 6;14(6):e0217836. doi: 10.1371/journal.pone.0217836 (PMC6553726; doi:10.1371/journal.pone.0217836)
Supplement: S2 File — (DOCX) [file pone.0217836.s002.docx]

| Ilwazi, imikghwa neendlela zokuziphatha kwabantu abatjha ngokukhandelwa kwe-HIV, ukubekezelela iingozi ze-HIV Kanye nokuhlolelwa i-HIV nemitjhoga eNkangala (ePhrovinsini yeMpumalanga) kanye neeyingini ze-OR Tambo (e-Eastern Cape Province) **Irhelomibuzo lephenyo le-KAP** | | | | | | | | | | |
| --- | --- | --- | --- | --- | --- | --- | --- | --- | --- | --- |
| **ISIGABA A : UKUHLELWA KOKUHLUNGWA AWAKUZIVEZA** | | | | | | | | | | |
| **A1 : INDAWO** | | | | | | | | | | |
|  | Iphrovinsi | | | | | Mpumalanga  Eastern Cape | | | | 1  2 |
|  | Isiyingi | | | | | Nkangala  OR Tambo | | | | 1  2 |
|  | IInomboro yerhelomibuzo lomuzi | | | | |  | | | | |
|  | Inomboro yomuntu ophendulako | | | | |  | | | | |
|  |  | | | | | | | | | |
| **A2 : IMINININGWANA YOHLUNGWAKO** | | | | | | | | | | |
|  |  | Ilanga | Inyanga | Umnyaka | Ikhowudu Iyeikhathi | | | Ikhowudu yependulo | | |
|  | Ivakatjho lokuthoma |  |  |  | __ : __ | | |  | | |
|  | Ivakatjho lesibili |  |  |  | __ : __ | | |  | | |
|  | Ivakatjho lesithathu |  |  |  | __ : __ | | |  | | |
|  | Ikhowudu yependulo yokugcina | | | | | | | |  | |
|  |  | | | | | | | | | |
| **Ikhowudu yependulo** 1 = Ukuhlungwa kuphelile  2 = Kwenziwe isibekisosikhathi mayelana nokuhlungwa  3 = Ukukhethwa kophendulako ongekho ekhaya 4 = Ukwala kwehloko yekhaya  5 = Ukwala kophendulako 6 = Okhunye | | | | | | | | | | |
|  |  |  |  |  |  | | |  | | |
| Amanothi wevakatjho (Imibono) | | | | | | | | | | |
|  | | | | | | | | | | |
|  | ISIKHATHI SOKUTHONYWA KOKUHLUNGWA: | | | | | | __ : __ | | | |
|  | ISIKHATHI SOKUPHELA KOKUHLUNGWA: | | | | | | __ : __ | | | |
|  |  | | | | | |  | | | |
|  | IBIZO LOMHLUNGI: | | | | | |  | | | |

| **ISIGABA B : IMINININGWANA YOWALAKO (NANGABE IYATHOLAKALA)** | | | | | | |  |  |  |  |  |  |  |  |
| --- | --- | --- | --- | --- | --- | --- | --- | --- | --- | --- | --- | --- | --- | --- |
|  |  | | | | | | |  |  |  |  |  |  |  |
| **Q#** | **Umbuzo** | **ipendulo** | **ikhowudu** | | **dlula** | |  |  |  |  |  |  |  |  |
| A3 | Wale akuphi ophendula imibuzo? | | | | | |  |  |  |  |  |  |  |  |
|  | Aseyegeni nanyana emnyango Ngemva kwehlathululo yephenyo kanye nekambiso  Ngemva kobana ipendulo yokuthoma ifunyenwe (ngaphambi kokuhlungwa) Lokha nakuhlungwa umuntu ngamunye Ngemva kokuhlungwa komuntu ngamunye nakakhonjelwa bona ahlolwe  Okhunye_____________________________________________________ | | | 1 2 3 4 5 6 | |  |  |  |  |  |  |  |  |  |
| A4 | Ufisa ukungitjela bona kubayini ungafuni ukuzibandakanya? [Akukafaneli bona ungitjele.] | | | | | |  |  |  |  |  |  |  |  |
|  | **Ukwala zisasuka phasi** | | | 1 2 3 4 5 6 7 8 9 10 | |  |  |  |  |  |  |  |  |  |
|  | Ngimatasatasa ukobana ngingahlungwa Angikho kwanjesi Kusebusuku khulu  Angifuni ukuzibandakanya kwelinye nelinye iphenyo/ukuhlungwa Angikhambisani neenhloko zephenyo ze (HIV/AIDS) Angifuni ukuhlungwa mhlungi othileko Ngiyasaba Ngesaba bona ngizakuphula umthetho wefihlo Umbuso awungenzeli litho Okhunye________________________________________________________________ | | |  |  |  |  |  |  |  |  |  |  |  |
|  | **Ukwala lokha kwenziwa ukuhlungwa komuntu ngamunye** | | | 11 12 13 14 15 16 17 18 | |  |  |  |  |  |  |  |  |  |
|  | Ukwala ukutjho nanyana yini/ elinye ilwazi ngesihloko Ukwala ukunikela imininingwanakhe/ilwazi eliyifihlo Ukubhalelwa kunikela ilwazi elifunekako Ukwala ukuragela phambili ngombana usilingekile/uzizwe adonda Ukwala ukuragela phambili ngombana ukwatile Ukwala ukuragela phambili ngombana angasenakareko nanyana uzizwe adiniwe Ukwala ukuragela phambili ngombana bekarhabile Okhunye_________________________________________________________________ | | |  |  |  |  |  |  |  |  |  |  |  |
| **Amanothi wokwala** | | | | | | |  |  |  |  |  |  |  |  |
|  | | | | | | |  |  |  |  |  |  |  |  |

| **Q#** | **Ihlathululo** | **Ipendulo** | **Ikhowudu** |
| --- | --- | --- | --- |
| A5 | Ophendulako uselawini lapha kunguye nomhlungi kwaphela. | Iye  Awa | 1  2 |
| A6 | Ngilifundile iphepha elinelwazi ngomuntu, isitatimende sobuntumba nanyana sefihlo neforomo lelwazi lemvumo | Iye  Awa | 1  2 |
| A7 | Nangabe umhlanganyeli uyavuma ukuzibandakanya, ulitlikitlile iforomo lemvumo? | Iye  Awa | 1  2 |
| A8 | Ingabe umhlanganyeli uliphethe ikhophi letjhidi lelwazi? | Iye  Awa | 1  2 |

| **ISIGABA 1 : IMINININGWANA YALAPHA OPHENDULAKO AHLALA KHONA** | | |
| --- | --- | --- |
|  | | |
| **Q#** | **Umbuzo** | **ipendulo** |
| 1.1 | Bewuneminyaka emingaki ngelanga lakho lamabeletho lokugcina? (*Iminyaka yophendulako*) | _______ iminyaka |
| 1.2 | Wabelethwa nini? (*YYYY/MMM/DD*) | ____ /____ /___ |
| **TJHEJA – NANGABE OPHENDULAKO UNGEHLA KWEMINYAKA ELI-18 UBUDALA NANYANA UNGAPHEZU KWEMINYAKA EMA-24 NGELANGA LOKUHLUNGWA, JAMISA MSINYA UKUHLUNGWA BESE UTHOKOZA UMUNTU LOYO** | | |

| **Q#** | **Question** | **responses** | **code** | **Skip** |
| --- | --- | --- | --- | --- |
| 1.3 | Sekusikhathi esingangani uhlala lapha? | Ngehla kweemveke ezi-4. Ukudlula inyanga eyodwa, kodwana ngaphasi kweenyanga ezisi-6. Ukudlula iinyanga ezisi-6 kodwana ngaphasi komnyaka owodwa. Ukudlula umnyaka owodwa (1), kodwana ngaphasi kweminyaka emi-5. Iminyaka edlula emi-5. Ubuphilo bami boke. | 1 2  3  4  5 6 |  |
| 1.4 | Ubulili bophendulako [***Ungabuzi, tlola phasi ubulili***] | Umsikazi Umduna | 1 2 |  |
| 1.5 | Buyini ubujamo bakho bomtjhado banje? (Ubujamo bomtjhado obutjho wangokomthetho, wesintu nanyana wamalobolo) | | | |
|  | angikendi Angikendi nanyana angihlalisani nomuntu, kodwana nginomuntu engihlekisana naye sekudlula iinyanga ezi-3 sihlekisana Angikendi, kodwana ngihlala nomuntu engihlekisana naye/isokana/udade  Ngitjhadile, ngihlala nomlingani Ngitjhadile, kodwana angihlali nomlingani Sihlukene/Ngimfelokazi Okhunye (itjho)________________________________________________ | | 1 2  3 4 5 6 7 |  |
| **UMLAYEZO** : *Ngizokubuza ngobujamo bakho bomsebenzi* | | | | |
| 1.6 | Ungabuhlathulula njani ubujamo bakho bomsebenzi wanje? | | | |
|  | Ngingumma otlhogomela ekhaya, ohlwengisa ekhaya, angiwufuni umsebenzi Ngingumma otlhogomela ekhaya, ohlwengisa ekhaya, ngiwufuna umsebenzi  Angisebenzi, ngifuna umsebenzi Angisebenzi, angiwufuni umsebenzi Ngisebenza endaweni engakahleleki, ngifuna umsebenzi wasafuthi Ngisebenza endaweni engakahleleki, angiwufuni umsebenzi wasafuthi Ngiyagula/ngikhubazekile begodu angikghoni ukusebenza Ngimfundi Ngiyazisebenza – woke malanga (ama-iri ama-40 nanyana adlulako ngeiveke) Ngiyazisebenza - kwamatorhwana (ama-iri angehla kwama- 40 ngeveke) Ngisebenza amatorho (nangabe akukho kokungehla) (ngehla kwama-iri ama-40 ngeveke) Ngisebenza safuthi (ama-iri ama-40 nanyana adlulako ngeveke)  Okhunye________________________________________________ | | 1 2 3 4 5 6 7 8 9 10 11 12 13 |  |

| 1.7 | Ngiliphi izinga lefundo ephezulu oliqedileko? | | |  |  |
| --- | --- | --- | --- | --- | --- |
|  | Angifundi Igreyidi 1/Isab a/Itlasi 1 Igreyidi 2/Isab b/Itlasi 2 Igreyidi 3/Ibanga 1 Igreyidi 4 /Ibanga 2 Igreyidi 5 /Ibanga 3 Igreyidi 6 /Ibanga 4 Igreyidi 7/Ibanga 5 Igreyidi 8 /Ibanga 6 Igreyidi 9 /Ibanga 7 Igreyidi 10/Ibanga 8 Igreyidi 11/Ibanga 9 Igreyidi 12/Ibanga 10/ Imethrigi Iimfundo eziragela phambili azingakapheli Isitifikeyidi sekhosi engiyiqede nangiphuma esikolwenil (Igreyidi 12/Imethrigi) IDiploma/Idigri ngemva kwemethrigi Ngiqede enye idigri | | | 1  2  3  4  5  6  7  8  9  10  11  12  13  14  15  16  17 |  |
| 1.8 | Eenyangeni ezili-12 ezidlulileko, khese kwenzeka (wena/ nanyana abanye abantu abadala ngakwenu) nakhe naphungula ukudla kwenu ngesimanga sokobana bekunganakudl okwaneleko? | | Iye  Awa | 1  2 |  |
| 1.9 | Eenyangeni ezili-12 ezidlulileko, khewalamba kodwana wabhalelwa kukudla ngesimanga sokobana bewunganamali eyaneleko yokuthenga ukudla? | | Yes  No | 1  2 |  |
|  | Nangabe uphe iye embuzweni 1.8 naku 1.9 phendula umbuzo 1.9a | |  |  |  |
| 1.9a | Ngisiphi isizathu sokobana nehlise izinga lokudla namkha nilambe kodwana ningadli? | | Akuna kudla  Akunamali  yokuthenga  ukudla  Kwehlisa umzimba  okhunye | 1  2  3  4 |  |
| 1.10 | Ngikuphi kilokhu okulandelako okuhlathulula indlwakho kuhle? | Indlu yeentina  Iflede  Indlu engemva kwendlu (izozo)  Umtlhatlhana (umkhukhu) | | 1  2  3  4 |  |
| 1.11 | Ngikuphi onakho endlinakho kilokhu okulandelako | Igezi  Umrhatjho  Umabonakude  Umaliledinini  Ifriji  Angikhumbuli  Ngala ukuphendula  Itsikiri  Isithuthuthu  Imodere  Ingadi yokudla  Ifuyo | | 1  2  3  4  5  6  7  8  9  10  11  12 |  |

| 1.12 | Ingabe ulilunga lananyana ngiyiphi itlabha nanyana isiqhema nanyana isosayidi? | Iye, ngililunga  Iye, ngililunga elimajadu  Awa | 1  2  3 |  |
| --- | --- | --- | --- | --- |
| 1.13 | Ingabe uyayithola imali yesondlo? | Iye  Awa | 1  2 |  |
| 1.14 | Uzihlathulula njani ngokobutjhaba? | Ngilikhuwa  Ngim-Afrika  NgiliNdiya  NgiliKhaladi  Okhunye, Itjho __________ | 1  2  3  4  5 |  |
| 1.15 | Uyitholaphi imali ukobana ubhadalele iindingo zaqobe nyanga?  [Khetha kunye] | Imali yesondlo  Umrholo obuya kumqatjhi  Inzuzo yebhizinisi  Engihlekisana naye/umlingani e  Abentwana/umndeni  “Ibhlesa”/ “umuntu omdala”  Okhunye, kutjho……….. | 1  2  3  4  5  6  7 |  |

| **ISIGABA 2 : ILWAZI NEMIBONO NGE-HIV** | | | | | | | | |
| --- | --- | --- | --- | --- | --- | --- | --- | --- |
|  |  |  |  |  |  |  |  |  |

| **Q#** | **Imibuzo** | **impendulo** | | **ikhowudu** | | **dlula** |
| --- | --- | --- | --- | --- | --- | --- |
| 2.1a | **Phendula yoke imibuzo** | | | | | |
|  |  | | Iye | | Awa |  |
|  | a. Ukukhandela ukugula kwe HIV ikhondomu fanele isetjenziswe mahlandla woke abantu nabasemsemeni | | 1 | | 2 |  |
|  | b. Umuntu angehlisa ubungozi bokungenywa yi HIV ngokungabi nabalingani abanengi | | 1 | | 2 |  |
|  | c. Ingabe umuntu oqaleka aphile kuhle angaba nobulwelwe be HIV? | | 1 | | 2 |  |
|  | d. Ingabe i AIDS ingalapheka na? | | 1 | | 2 |  |
|  | e. Ingabe umuntu angathola I HIV ngokudla nomuntu onayo? | | 1 | | 2 |  |
|  | 1. f. Ingabe I HIV ungayigongobaza na? | | 1 | | 2 |  |
| 2.1b | Ungangitjela zoke iindlela ozaziko ithelelwano le-HIV elingakhandeleka ngazo? [**UMLAYEZO: IIMPENDULO EZIBUYELELWEKO OKUNGABA NGIZO** | | | | | |

|  | Angeze yakhandeleka Ngokusebenzisa amakhondomu Ngokuthandana nomlingani munye Ngokuthembeka kiloyo ohlekisana naye okuthi naye athembeke kuwe Ngokwehlisa inani labantu othandana nabo Ngokulisa ungasayi emsemeni Ngokubalekela ukubamba iingazi Ngokusebenzisa iindakamizwa ukobana ukhandele ukudlula kweengazi kumma ziye emntwaneni Ukusokwa kobudoda  Imikhrobhayisayidi (ijeli/irenghi efakwa ngaphakathi kwesitho sakamma ukukhandela ithelelwano le-HIV) Nge-Pre-exposure prophylaxis (PrEP) Nge-Post-exposure prophylaxis (PEP) Okhunye (itjho)_____________________________________ Angazi | | 1 2 3 4 5 6 7 8 9 10  11 12 13 14 |  |
| --- | --- | --- | --- | --- |
| 2.2 | Ingabe ikhona imitjhoga eyelapha i-HIV ne- AIDS? **TJHEJA: FUNDELA PHEZULU IHLATHULULO [Umtjhoga kutjhiwo into ezokwenza abantu bahlale baphilile isikhathi eside & ingasi into eyelapha ubulwele]** | Iye Awa Angazi | 1 2 3 | ***Tjhinga ku 2.5 nangabe i ngu 2 nanyana 3*** |
| 2.3 | Yini imitjhoga? **[UMLAYEZO: UNGAFUNDI OKUKHETHWAKO. IIMPENDULO EZIBUYELELWEKO OKUNGABA NGIZO]** | | | |
|  | Imitjhoga eziindakamizwa yama-Anthirithrovayirali (ama-ARVs/ART)  Ezinye iindakamizwa, isihlahla, amapilisi Isihlahla sesintu Iimbiza ezenziwa ekhaya  Okwenza ungangenwa malwele Imithandazo Okhunye (itjho)__________________________ Angazi | | 1 2 3 4 5 6 7 8 |  |
| 2.4 | Umuntu angathoma msinya nini ukuthatha imitjhoga ye-HIV ne-AIDS ngemva kobana ahlolwe atholwe aphosethivu? **[UMLAYEZO: UNGAFUNDI OKUKHETHWAKO. IIMPENDULO EZIBUYELELWEKO OKUNGABA NGIZO]** | | | |
|  | khonokho Lokha imbalo ye-CD4 ingenzasana (ingaphasi kuma- 500) Msinyazana nangabe umuntu ungumma osidisi Msinyazana nangabe umuntu une-TB Nanyana kunini lokha umuntu nakalungele ukuthola imitjhoga  Okhunye (itjho)___________________________ Angazi | | 1 2 3 4 5 6 7 |  |
| 2.6 | Abantu kufanele bathathe isikhathi esingangani ukusela imitjhoga ye-HIV ne-AIDS? | | | |
|  | Ubuphilo babo boke. Ikani lokha nabafunako. Bebazizwe bangcono. Bekufika lapha baphola khona Angazi. Okhunye(itjho)____________________________________ | | 1 2 3 4 5 6 |  |

| **UMTLOLO (SCRIPT) :** Ngifuna ukukubuza imibuzo emayelana nabantu abaphila ne-HIV ne-Aids **UMLAYEZO : [FUNDELA iintatimende PHEZULU]** 1 = Angivumi nakancani  2 = Angivumi  3 = Ngiyavuma  4 = Ngivuma khulu | | | | | |
| --- | --- | --- | --- | --- | --- |
|  | | **SD** | **D** | **A** | **SA** |
| 2.7.1 | Ngizakuhlala ngimngani womuntu nalokha nangithola bona une-HIV. | 1 | 2 | 3 | 4 |
| 2.7.2 | Nawuthola bona une-HIV, ubuphilo bakho buyaphela. | 1 | 2 | 3 | 4 |
| 2.7.3 | Ngingaphathwa ziinhloni nange ngibonwa nomuntu owaziwa nguwokewoke bona une-HIV. | 1 | 2 | 3 | 4 |
| 2.7.4 | Omunye nomunye umuntu othoma ukuthandana kufanele ahlolelwe i-HIV ngaphambi kobana aye emsemenzi. | 1 | 2 | 3 | 4 |
| 2.7.5 | Ukukhandela ukungenwa yi-HIV kufanele usebenzise amakhondomu ngaso soke isikhathi nawuya emsemeni nomuntu. | 1 | 2 | 3 | 4 |
| 2.7.6 | I-HIV idlula lula ngesikhathi seemveke ezisithandathu zokuthoma ngemva kobana umuntu imngene. | 1 | 2 | 3 | 4 |
| 2.7.7 | Indoda esokiweko akutlhogeki bona isebenzise ikhondomu ukukhandela ukungenwa yi-HIV | 1 | 2 | 3 | 4 |

| **ISIGABA 3 : ILWAZI, IMIKGHWA, NEMIBONO YABANTU NGOBULWELE BESIFUBA (I-TB)** | | | | | | | | | | | | |
| --- | --- | --- | --- | --- | --- | --- | --- | --- | --- | --- | --- | --- |
|  |  |  |  |  |  |  | |  | | |  | |
| **Q#** | **isitatimende** | | | | | | |  | | **Skip** | | |
| 3.1 | Uyasazi bona siyini isifo sofuba? | | | | | | Iye  Awa | 1  2 | | **Tjhinga kumbuzo3.1.1 ipendulo yakho ngu Iye** | | |
| **UMTLOLO (SCRIPT):** Ngizokubuza imibuzo ngobulwele besifuba (i-TB). Ngibawa wazi bona akunampendulo ezilungileko nezingakalungi   1 = Ngiyavuma (A)  2 = Angivumi (D)  3 = Angazi (DK) | | | | | | | | | | | | |
| **Q#** | **Statement** | | | | | | | **A** | **D** | | | **DK** |
| 3.1.1 | Omunye nomunye umuntu angangenwa yi-TB | | | | | | | 1 | 2 | | | 3 |
| 3.1.2 | Abantu abaphila ne-HIV kungabalula bona bangangenwa yi-TB | | | | | | | 1 | 2 | | | 3 |
| 3.1.3 | Abantu abanganayo i-HIV bangangenwa yi-TB | | | | | | | 1 | 2 | | | 3 |
|  |  | | | | |  | |  | | |  | |
| **Q#** | **Umbuzo** | | | | **Iimpendulo** | | | **Ikhowudu** | | | **Dlula** | |
| 3.2 | Umuntu ingamngena njani i-TB? | | | | | | | | | | | |

|  | Ngokulotjhisana Ngommoya lokha umuntu one-TB nakakhohlelako nanyana nakathimulako Ngokwabelana izitja Ngokudla ninoke esitjeni esisodwa Ngokuthinta izinto eendaweni zomphakathi (iimbambo zeminyango, iminyango yeenthuthi, njll.) Ngokubhema Okhunye (itjho)______________________________ Angazi | | | | 1 2 3 4 5  6 55 96 |
| --- | --- | --- | --- | --- | --- |
| 3.3 | Ngiziphi iintjengisi kanye namatshwayo we-TB? | | | | |
|  | [IIMPENDULO EZINENGI]  Buza: Nani godu? | | Ukukhohlela okuthatha isikhathi esidlula eemvekeni ezi-3 Ukukhohlela iingazi Ukungakhanuki ukudla Umgomani Ukujuluka ebusuku Ukuzehlela komzimba Okhunye (itjho)_______________________ Angazi | | 1 2 3 4 5 6 55 96 |
| 3.4 | Ngimiphi imitjhoga ye-TB? | Iindakamizwa, isihlahla, amapilisi Akunamitjhoga Isihlahla sesintu Angazi | | | 1 2 3 96 |
| 3.5 | Umuntu kufanele ayithathe isikhathi esingangani imitjhoga yokwelapha i-TB? | | | | |
|  | **[IPENDULO EYODWA KWAPHELA]** | | Inyanga yinye nanyana ngehla Iinyanga ezimbili ukuya kwezihlanu Iinyanga ezisithandathu nanyana ezidlulako Okhunye (itjho)______________________ Angazi | | 1 2 3 55 96 |
| 3.6 | Ingabe abantu abane-TB baphosethivu ngaso soke isikhathi? | | | | |
|  |  | | | Iye Awa Angazi | 1 2 96 |
| 3.7 | Ingabe kuyakghoneka ukwelapha i-TB ebantwini abane-HIV? | | | | |
|  |  | | Iye Awa Angazi | | 1 2 96 |
| **UMTLOLO (SCRIPT)**: Emphakathinakho, esikhathini esinengi uphathwa njani umuntu one-TB?  **UMLAYEZO : FUNDA ISITATIMENDE NGASINYE BESE UHLOLA IPENDULO EYODWA** 1 = Angivumi nakancani (SD)  2 = Angivumi (D)  3 = Ngiyavuma (A)  4 = Ngivuma khulu (SA) | | | | | |

|  | | | | | | | | | | | | | | | | **SD** | | | **D** | | | **A** | **SA** | | |  |
| --- | --- | --- | --- | --- | --- | --- | --- | --- | --- | --- | --- | --- | --- | --- | --- | --- | --- | --- | --- | --- | --- | --- | --- | --- | --- | --- |
| 3.8 | | Inengi labantu abamfuni | | | | | | | | | | | | | | 1 | | | 2 | | | 3 | 4 | | |  |
| 3.9 | | Inengi labantu liyamthanda kodwana inengi lizama ukumbalekela | | | | | | | | | | | | | | 1 | | | 2 | | | 3 | 4 | | |  |
| 3.10 | | Umphakathi esikhathini esinengi uyamsekela | | | | | | | | | | | | | | 1 | | | 2 | | | 3 | 4 | | |  |
|  | |  | |  | | |  |  | | | |  | | | | | | |  | | | | |  |  | |
| **Q#** | | **Umbuzo** | | | | | | | | | **Iimpendulo** | | | | | **Ikhowudu** | | | | **Dlula** | | | | | |  |
| 3.11 | | **Ingabe ukhona umuntu okhe waba ne TB endlinakho?** | | | | | | | | | | | | | | | | | | | | | | | |  |
|  | |  | | | | | | | | | **Iye**  **Awa**  **Akunampendulo** | | | | | | | **1**  **2**  **3** | |  | | | | | |  |
| 3.11a | | Wakhe wazihlolisela i-TB? | | | | | | | | | | | | | | | | | | | | | | | |  |
|  |  |  | | | | | | | | | Iye Awa Akumpendulo | | | | | | | 1 2 3 | | **If your answer is no, or no response please go to 4.1** | | | | | |  |
| 3.12 | | Sekukade kangangani wazihlolisela i-TB muva nje | | | | | | | | | | | | | | | | | | | | | | | |  |
|  |  |  | | | | | | | | | 0 ukuya eenyangeni ezi-3  4 ukuya eenyangeni ezisi- 6  7 ukuya eenyangeni ezili-11  Ngehla komnyaka owodwa odlulileko  Hlangana nomnyaka 1ukuya kwemi-2 edlulileko  Hlangana neminyaka emi-2 ukuya3 | | | | | | | 1 2 3  4  5  6  7 | |  | | | | | |  |
| 3.13 | | Wazihlolisela kuphi I TB? | | | | | | | | | Emtholampilo/esibhedlela  Kusiberegi somphakathi  Kumlaphi wendabuko  Ekhemisi  Okhunye  (hlathulula)________________ | | | | | | | 1  2  3  4  5  6 | |  | | | | | |  |
| 3.14 | | Ngikuphi abakuthetheko ukuhlolela I TB? | | | | | | | | | igazi  isikhohlela  okhunye  (hlathulula)_________________ | | | | | | | 1 2 3  4 | |  | | | | | |  |
| **ISIGABA5: UKUTJENZISWA KOTJWALA** | | | | | | | | | | | | | | | | | | | | | | | | |  |  |
|  | | |  | |  |  | | |  |  | | |  |  |  | | | | | | | | | |  |  |
| **UMTLOLO (SCRIPT) : Njenganje ngizokubuza imibuzo ngokusela utjwala** | | | | | | | | | | | | | | | | | | | | | | | | |  |  |
| **Q#** | **Umbuzo** | | | | | | | **Iimpendulo** | | | | | | | | | **Ikhowudu** | | | | **Dlula** | | | |  |  |
| 4.1 | Kukangaki lapha wakhe waberegisa khona isangu, ibhenzina, itiki, ikhokheyini, inyawupe, iwunga, amamandreksi, ihiroyini nanyana enye into ekwenza uzizwe uphezulu | | | | | | | Akhange  Pheze ngaso soke isikhathi  Kanengi kodwana ingasi ngasosoke iskhathi  Kwenzeke qobe yiveke | | | | | | | | | 0  1  2  3 | | | |  | | | |  |  |
| 4.2 | Esikhathini esinengi ubusela kangaki utjwala? | | | | | | | Akhange khengibubeke emlonyeni qobe yiveke  Ka-3 ngenyanga ngehla kwenyanga eyodwa ngehla kweenyanga ezisi-6 | | | | | | | | | 0  1  2  3 | | | |  | | | |  |  |

| 4.3 | Bungaki utjwala obusela qobemalanga | 1 or 2  3 or 4  5 or 6  7, 8 or 9  10 or more | 0  1  2  3  4 |  |
| --- | --- | --- | --- | --- |
| 4.4 | Kukangaki lapho osela khona amabhodlelo asithandathu (6) namkha ukuya phezulu ngesikhathi sinye? | Akhange  Ngaphasi kwenyanga yinye  Qobe nyanga  Qobe veke  Malanga woke | 0  1  2  3  4 |  |
| 4.5 | Kukangakhi enyakeni odlulileko lapho obhalelwe kwenza izinto okufanele uzenze ngesibanga sokusela utjwala | Akhange  Ngaphasi kwenyanga yinye  Qobe nyanga  Qobe veke  Malanga woke | 0  1  2  3  4 |  |
| 4.6 | Kukangakhi enyakeni odlulileko lapho utlhoga khona utjwana ekseni kuze ukhone ukuphaphama ngesibanga sokuthi usele khulu utjwala | Akhange  Ngaphasi kwenyanga yinye  Qobe nyanga  Qobe veke  Malanga woke | 0  1  2  3  4 |  |
| 4.7 | Kukangakhi enyakeni odlulileko lapho wazithola uzizwa mlandu namkha uzisola ngemva kobana usele utjwala? | Akhange  Ngaphasi kwenyanga yinye  Qobe nyanga  Qobe veke  Malanga woke | 0  1  2  3  4 |  |
| 4.8 | Kukangakhi enyakeni odlulileko wazithola ubhalelwa kukhumbula okwenzeke ebsukwini bangayizolo ngesimanga sokuthi bewusela utjwala? | Akhange  Ngaphasi kwenyanga yinye  Qobe nyanga  Qobe veke  Malanga woke | 0  1  2  3  4 |  |
| 4.9 | Ukhe namkha omunye umuntu walimala ngesimanga sokusela kwakho? | Awa  Iye, kodwana ingasi enyakeni odlule  Iye, enyakeni odlule | 0 1 2 |  |
| 4.10 | Ingabe umngani namkha isihlobo namkha isiberegi sezamaphilo bakhe batshwenyeka ngokusela kwakho namkha bakukhombela bona wehlise izinga lokusela kwakho? | Awa  Iye, kodwana ingasi enyakeni odlule  Iye, enyakeni odlule | 0 1 2 |  |

| **ISIGABA 5 :UMLANDO NGEZOMSEME** | | | | | | | | |
| --- | --- | --- | --- | --- | --- | --- | --- | --- |
|  |  |  |  |  |  |  |  |  |
| **UMLAYEZO:** Akukafaneli ngikubuze imibuzo elumelako ngeendaba eziphathelne nezomseme nokhunye okuphathelene nezomseme. Ngibawa ukhumbule bona ibizo lakho angeze latlolwa ndawo kileliphephamibuzo begodu ilwazi olivezako lizakubekwa endaweni efihlekileko. | | | | | | | | |

| **Q#** | **Umbuzo** | | **Iimpendulo** | | **Ikhowudu** | | | **Dlula** |
| --- | --- | --- | --- | --- | --- | --- | --- | --- |
| 5.1 | Wakhe waya emsemeni?  [Ngokomnqopho wephenyweli*, “ukuya emsemeni” ikuhlathululwa njengokulalana ngaphambili/ngemva emahlalweni*.] | | | Iye Awa | | 1 2 | | **Tjhinga kumbuzo 5.5** |
| 5.2 | Ngibawa ungitjele bona kubayini ungakathomi ukuya emsemeni? | | | | | | | |
|  |  | Angikakulungeli Ngisesemncani Anginakareko Ngibalekela ukuba sidisi Ngibalekela ama- STIs, kufakwe hlangana i-HIV Ngokweenzathu zekolo Ngokweenzathu zamasiko Anginaye umuntu engithandana naye  Ayikho indlela engingathola ngayo amakhondomu Anginayo ipendulo Okhunye (itjho)_________________________ | | | | 1 2 3 4 5  6 7 8 9  10  11 | |  |
| 5.3 | Wawuneminyaka emingaki lokha nawuya emsemeni kokuthoma? **[tlola ubudala ngeminyaka]** | _________ iminyaka Angiyikhumbuli iminyaka | | | | 1 | |  |
| 5.4 | Walalana nobani lokha nawuya emsemeni kokuthoma? | | | | | | | |
|  |  | Isokanalakho/intombakho Umngani Umyenami/umfazami Umuntu ebenginelanga linye nanyana isikhatjhana ngimazi Ilunga lomndeni/isihlobo Umuntu engimaziko kodwana bekangasuye umngani Mumuntu engingamaziko | | | | | 1 2 3 4  5 6  7 |  |
| 5.5 | Ungalihlathulula njani ilemuko lakho lokuthoma lokuya emsemeni? | | | | | | | |
|  |  | Ngangifuna Ngayengwa Ngakatelelwa Ngakatwa | | | | | 1 2 3 4 |  |

| 5.6 | Wayiseberegisa ikhondomu kokuthoma nawuya emsemeni? | Iye Awa Angikhumbuli | 1 2 3 |  |
| --- | --- | --- | --- | --- |

|  | |  | |  |  | |  | | |  | |  | |  | | |  |  |
| --- | --- | --- | --- | --- | --- | --- | --- | --- | --- | --- | --- | --- | --- | --- | --- | --- | --- | --- |
| **Q#** | **Umbuzo** | | | | | | | | | | **Iimpendulo** | | | | **Ikhowudu** | **Dlula** | | |
| 5.7 | Ukhe waya emsemeni eenyangeni ezili-12 ezidlulileko? | | | | | | | | | | Iye Awa Akumpendulo | | | | 1 2 3 | **Nangabe ipendulo ngu Awa namkha akunapendulo tjhinga ku 5.4** | | |
| 5.8 | Sebaboke, bangaki abantu okhe waya emsemeni nabo ubuphilo bakho boke? **BUZA** : NANGABE ‘00’, HLATHULULA KUHLE IPENDULO KUMBUZO 5.1 | | | | | | | | | | | | | | _____ |  | | |
| 5.9 | Waba nabantu abangaki owaya nabo emsemeni hlangana neenyanga ezili-12 ezidlulileko? | | | | | | | | | | | | | | _____ |  | | |
| 5.10 | **Umuntu wengubo:** Wakhe waya emsemeni nomuntu ngombana bewuthemba nanyana bewulindele bona uzakupha imali nanyana uzakupha okuthileko?  **Umuntu omduna:** wakhe waya emsemeni nomuntu gombana bewumthembise ukumupha imali nanyana okuthileko? | | | | | | | Ngathola imali nanyana izipho nanyana isithuthi  Ngathola ukudla nanyana isiselo nanyana ngaba nesikhathi esimnandi Awa | | | | | | | 1  2  3 |  | | |
|  |  | |  |  | | | |  | | |  | |  | |  |  | | |
| **UMLAYEZO** : Buza imibuzo naso nangabe utlole abalingani abambalwa ngehla **UMTLOLO (SCRIPT)** :Njenganje ngibawa ukukhuluma nawe ngemisebenzi yomseme ngokuvamileko | | | | | | | | | | | | | | | | | | |
| 5.11 | Ingabe amanye wamatjhebiswano avezwe ngehla bekathomana? | | | | | | | | | | Iye Awa Anginapendulo | | | | 1 2 3 |  | | |
| 5.12 | Njenganje unabalingani ababili nanyana abambalwa oya nabo emsemeni? | | | | | | | | | | Iye Awa Anginapendulo | | | | 1 2 3 |  | | |
| 5.13 | Sebaboke, bangaki abalingani abahlukahlukeneko oya nabo emsemeni okhe waba nabo eenyangen ezi-3 ezidlulileko? | | | | | | | | | | | | | | _____ |  | | |
| 5.14 | Bewuwasebenzisa amakhondomu nomlingani ngamunye eenyangeni ezi-3 ezidlulileko? | | | | | | | | | | Iye Awa | | | | 1 2 |  | | |
| 5.15 | Liyini itjhebiswano lakho nomlinganakho oya naye emsemeni wanje? | | | | | Myenami / Mkami Mlingani engihlalisana naye Yintombi / Lisokana elingahlali nami Mlingani esinande sibonana naye Mumuntu engimbhadalako ukobana siye emsemeni  Okhunye(itjho) _________________________ | | | | | | | | | 1 2 3 4 5  6 |  | | |
| 5.16 | Ingabe umlingani wanje lo oya naye emsemeni umumuntu osikazi nanyana omduna? | | | | | | | | Omduna Omsikazi | | | | | | 1 2 |  | | |

| 5.17 | Umlinganakho lo uhlala kuphi? | | Endaweni engihlala kiyo Kwenye indawo Sihlala endlini eyodwa | | 1 2 3 |  |
| --- | --- | --- | --- | --- | --- | --- |
| 5.18 | Uneminyaka pheze emingaki umuntu lo? [**tlola ubudala ngeminyaka**] | | _________ iminyaka Angazi | | 88 |  |
| 5.19 | Wayisebenzisa ikhondomu ngesikhathi uya emsemeni kwamaswaphela? | | Iye Awa | | 1 2 |  |
| 5.20 | Nangabe wayisebenzisa ikhondoomu, bekuyini iinzathu zakho zokwenza lokho? | | | | | |
|  | Ngiyelela ukuba nethelelwano le-HIV Abantu kuthiwa basebenzise amakhondomu Ngifuna ukukhandela ama- STI’s  Ngifuna ukukhandela ukuba sidisi Mina nanyana umlingani sikuma- ARV Umlingani ukatelele bona sisebenzise ikhondomu Okhunye: sibawa utjho___________________________________________________ | | | | 1 2 3 4 5 6 7 | **Nangabe ipendulo yakho ngu Awa tjhinga kumbuzo 5.21** |
| 5.21 | Nangabe AKHANGE usebenzise ikhondomu, bekungiziphi iinzathu zakho zokwenza lokho? | | | | | |
|  | Benginganayo ikhondomu Umlingani walile Ngisebenzise ezinye iinkhandelambeleko Angiwathandi Akhange ngicabange bona kuyatlhogeka  Ngitjhadile Ngiyathembeka/ngiyabethemba  Ngangidakiwe/nganginesiyeziyezi okhunye(itjho)_______________________________________________ | | | | 1 2 3 4 5 6 7 8  9 10 | Qubekela kumbuzo 5.22 |
| 5.22 | Ingabe **kulula** ukuthola ikhondomu yabaduna nangabe uyayifuna? | | | Iye Awa Anginapendulo | 1 2 3 |  |
| 5.23 | Ingabe **kulula** ukuthola ikhondomu yabasikazi nangabe uyayifuna? | | | Iye Awa Anginapendulo | 1 2 3 |  |
| **UMALYEZO : Buza imibuzo elandelako (Q5.19) kilabo abamajadu abangazange basebenzise ikhondomu** | | | | | | |
| 5.24 | Esikhathini esinengi vane uwathole kuphi amakhondomu wakho? | | | | | |
|  | [UNGAFUNDELI AMAKHETHO LA PHEZULU. KUNGABA KHONA IIMPENDULO EZINENGI ] | Etlinigi/esibhedlela ekhemisi esitolo/ekhefi/esiphaza egaratjhi/ esitetjhini seembaseli esipotini/ethaveni/ebhareni kubangani kumlinganami  kubabelethi  Akukho  Kusiberegi sezamaphilo emphakathini Okhunye (itjho)____________________________ | | | 1 2 3 4 5 6 7 8  9  10 |  |

| **ISIGABA 6 : UKUKHANSELELWA I-HIV NOKUHLOLWA** | | | | | | | | |
| --- | --- | --- | --- | --- | --- | --- | --- | --- |
|  |  |  |  |  |  |  |  |  |
| **UMTLOLO (SCRIPT):** Ngithanda ukukubuza imibuzo mayelana nokuhlolelwa i-HIV. Ngibawa ukhumbule bona ibizo lakho angeze litlolwe ndawo kileliphephamibuzo begodu ilwazi olivezako lizakuba yifihlo. | | | | | | | | |

| **Q#** | **Umbuzo** | | **Iimpendulo** | | **Ikhowudu** | | **Dlula** |
| --- | --- | --- | --- | --- | --- | --- | --- |
| 6.1 | Wakhe wahlolelwa i-HIV? | | | Iye Awa | | 1 2 |  |
| 6.2 | Ingabe kunendawo eseduze oyaziko lapha ungahlolelwa khona i-HIV? | | | Iye Awa | | 1 2 |  |
| 6.3 | Sekukade kangangani lapha ukhese wahlolelwa khona i-HIV msinyazana nje? | | | | | | |
|  |  | 0 ukuya eenyangeni ezi-3  4 ukuya eenyangeni ezisi-6  7 ukuya eenyangeni ezili-11  Ngehla komnyaka owodwa odlulileko Hlangana nomnyaka mu-1ukuya kwemi-2 edlulileko Hlangana neminyaka emi-2 ukuya kwemi-3 edlulileko Iminyaka emithathu nanyana eyeqako edlulileko | | | | 1 2 3 4 5  6  7 |  |
| 6.4 | Wazihlolisela kuphi I HIV mva nje? | Esibhedlela somphakathi Esibhedlela sangasese Etlinigi yomphakathi nanyana kudorhodera Etlinigi yangasese nanyana kudorhodera [Ibizo le-NGO kileyondawo/ihlangano enzinze emphakathini] Emberegweni Kusehlakalo sokuhlola umphkathi Kwenye____________________________________________ | | | | 1 2 3 4 5 6 7 8 |  |
| 6.5 | Ungakhetha ukuyozihlolisela kuphi I HIV? | Esibhedlela somphakathi Esibhedlela sangasese Etlinigi yomphakathi nanyana kudorhodera Etlinigi yangasese nanyana kudorhodera [Ibizo le-NGO kileyondawo/ihlangano enzinze emphakathini] Emberegweni Kusehlakalo sokuhlola umphakathini  Ekhaya Kwenye____________________________________________ | | | | 1 2 3 4 5 6 7 8 |  |

| **Umtlolo:** tjheja bona akutlhogeki bona uveze ubujamo bakho be-HIV, nginekareko yokwazi bona wazisiwe namkha utjeliwe ngemiphumela |
| --- |

| 6.6 | Nawuhlolelwa i-HIV kokugcina, wanikelwa ilwazi ngaphambi kobana uhlolelwe i-HIV? | | Iye Awa | 1 2 |  |
| --- | --- | --- | --- | --- | --- |
| 6.7 | Nawuhlolelwa i-HIV kokugcina, wanikelwa ilwazi kungaba nisiqhema nanyana uwedwa ngaphambi kobana uhlolelwe i-HIV? | | Ngingedwa Ngesiqhema | 1 2 |  |
| 6.8 | Watjelwa/waziswa ngemiphumela yokuhlolwa kwakho okwenze muva nje? | | Iye Awa | 1 2 |  |
| 6.9 | Wakhanselwa ngemva kokuhlolelwa i-HIV nawuhlolelwa i-HIV muva nje? | | Iye Awa | 1 2 |  |
| 6.10 | Kwakungiziphi iinzathu zakho zokobana uye kokugcina nje uyozihlolisela i-HIV? | | | | |
|  | **UMLAYEZO**: **UNGAFUNDELI AMAKHETHO LA PHEZULU. KUNGABA KHONA IIMPENDULO EZINENGI** | Bengifuna ukwazi ubujamo bami be-HIV Umlinganami ungibawe bona ngiyozihlolisa Bengifuna ukuthoma ithando elitjha lokuya emsemeni Bengifuna ukutjhada Ngifake isibawo setjhorensi Ngifake isibawo sokuboleka imali Umqatjhami wakubawa Bengizizwa ngigula Ngatjelwa sisebenzi sezamaphilo (linese/ngudorhodera) Ngangisidisi Lijima lemsebenzini Okhunye | | 1 2 3  4 5 6 7 8 9  10 11 12 |  |
| 6.11 | Uvezile bona esikhathini esidlulileko wakhe wahlolelwa i-HIV. Ingabe uyafuna ukungitjela ngemiphumela yokuhlolwa kwakho kwe-HIV owayitholako? | | Iye Awa Akhange ngiyithole | 1 2 3 | **Nangabe ipendulo ngu Awa tjhinga kumbuzo 6.16** |
| 6.12a | Kwaba yini imiphumela yokuhlolelwa i-HIV leyo? | | Phosethivu Negethivu Ayaziwa | 1 2 3 | **Nangabe ipendulo**  **ithi-negethivu namkha ayaziwa tjhinga kumbuzo 6.15** |

| 6.12b | Ngiyiphi inyanga nomnyaka wokuhlolwa kwakho okwaba phosethivu kokuthoma? **NANGABE ‘AWUYAZI’ INYANGA, YEKE TLOLA ‘88’ NANGABE ‘AWUWAZI’ UMNYAKA, YEKE TLOLA ‘8888’** | | | **INYANGA** | ___ | |  |
| --- | --- | --- | --- | --- | --- | --- | --- |
|  |  |  |  | **UMNYAKA** | ___ | |  |
| 6.13 | Ingabe njenganje uthatha ama-ARV, okutjho bona usela iinhlahla ze-HIV ama-antirithrovayirali? | | | Iye Awa | 1 2 | | **Nangabe ipendulo ngu Awa tjhinga kumbuzo 6.16** |
| 6.14 | Sekusikhathi esingangani usela ama-ARV qobe lilanda?  **TLOLA IPENDULO NGEENYANGA NANGABE KUNGAPHASI KOMNYAKA OWODWA. TLOLA '00' NANGABE KUNGEHLA KWENYANGA EYODWA.** | | | Inani leenyanga | ___ | |  |
|  |  |  |  | Inani leminyaka | ____ | |  |
| 6.15 | Umazisile umlingani wakho okunguyena ngemiphumela yakho ye HIV? | | | Iye Awa | 1 2 3 | |  |
| 6.16 | Ingabe ubatjelile abanye abalingani bakho ohlekisana nabo njenganje ngemiphumela yokuhlolwa le eenyangeni ezili-12 ezidlulileko? | | | Iye Awa Anginamlingani | 1 2 3 | |  |
| 6.17 | Wakhe wayozihlolisela i-HIV nabanye babalingani bakho oya nabo emsemeni lapha nathola imiphumela ninobabili? | | | Iye Awa | 1 2 | |  |
|  | | | | | | | |
| 6.18 | Ngiziphi iinzathu ezenza bona ungayi ukuyokuhlolelwa i-HIV? | | | | | | |
|  | **UMLAYEZO**: **UNGAFUNDELI AMAKHETHO LA PHEZULU. KUNGABA KHONA IIMPENDULO EZINENGI** | Angazi bona ngizihlolise kuphi Angicabangi bona ngine-HIV Angisisengozini yokuba ne-HIV Ngethemba umlinganami Ngiyesaba ukuzwa bona angaba-HIV phosethivu Angikabi ukulungela ukuhlolelwa i-HIV  Ngikhathelela UKUBA NEFIHLO Ngikhathalela UKUBANDLULULWA, nanyana UKUBEKELWA NGEQADI Ngikhathalela UKULOBA UMSEBENZAMI Ngikhathalela IZINGA LOMSEBENZI Akhange khengibekhona ukobana ngikwenze  Okhunye_______________________________________ | | | | 1 2 3 4 5 6 7 8  9 10 11  12 |  |
| 6.19 | Pheze bangaki abangani bakho ocabanga bona bahlolelwe i-HIV? | | | | | | |
|  | **UMLAYEZO**: **UNGAFUNDELI AMAKHETHO LA PHEZULU. KUNGABA KHONA IIMPENDULO EZINENGI  [TJENGISA IZANDLA ZOMBILI UKUJAMELA ABANTU ABALI-10.]** | | 1 kwabali- 10 2 kwabali- 10 3 kwabali 10 4 kwabali 10 5 kwabali 10 6 kwabali 10 7 kwabali 10 8 kwabali 10 9 kwabali 10 10 kwabali 10 | | | 1 2 3 4 5 6 7 8 9 10 |  |

| **UMTLOLO:** Uvumelana kangangani neentatimende ezilandelako mayelana nokuhlolelwa i-HIV emphakathinakho? Akunapendulo elungileko nengakalungi, mibono yabantu kwaphela. Sizakuthanda ukwazi bona ngimiphi imibono ovumelana nayo nanyana ongavumelaniko nayo.  **UMLAYEZO** : Mhlungi kufundele phezulu  **1 = Angivumelani nakancani (SD)  2 = Angivumelani (D)   3 = Ngiyavumelana (A)  4 = Ngivumelana khulu (SA)** |
| --- |

|  | | **SD** | **D** | **A** | **SA** |
| --- | --- | --- | --- | --- | --- |
| 6.20a | Abantu emphakathinami abahlolwe bazithola baphosethivu bazizwa bakhululekile ukutjela abangani nemindenabo bona bane-HIV. | 1 | 2 | 3 | 4 |
| 6.20b | Abantu emphakathinami abazihloliseli i-HIV ngombana basaba bona izakutjhugulula indlelabo yokuphila (okutjho bona ukusebenzisa amakhondomu, ukwehlisa inani labalingani, ukulisa ukusela utjwala nokubhema) | 1 | 2 | 3 | 4 |
| 6.20c | Abantu emphakathinami abayi ukuyozihlolisela i-HIV ngombana basaba bona vele seba-HIV phosethivu | 1 | 2 | 3 | 4 |
| 6.20d | Abantu emphakathinami bayasaba ukuyozihlolisela i-HIV ngombana bacabanga bona nange bazithola ba-HIV phosethivu angeze basaba semathandweni bathandane godu | 1 | 2 | 3 | 4 |
| 6.20e | Abantu emphakathinami abazihlolisela i-HIV bathathwa njengabantu abanesibindi nabazibopheleleko | 1 | 2 | 3 | 4 |
| 6.20f | Abantu emphakathinami bayasaba ukuyozihlolisela i-HIV ngombana bacabanga bona nange bazithola ba-HIV phosethivu imindenabo nabangani babo angeze isabamukela | 1 | 2 | 3 | 4 |

| **ISIGABA 7 :IMIBONO NGEENGOZI ZE-HIV , IMIKGHWA NEENDLELA ZOKUZIPHATHA EMPHAKATHINI NGOKUBA NABALINGANI ABAMBALWA KANYE NAMAKHONDOMU** | | | | | | | | |
| --- | --- | --- | --- | --- | --- | --- | --- | --- |
|  | ] |  |  |  |  |  |  |  |
| **UMTLOLO**:Angeze ngakubuza eminye imibuzo yokobana uzibona njani iingozi zokuba nethelelwano le-HIV | | | | | | | | |

]

| **Q#** | **Umbuzo** | **Iimpendulo** | **Ikhowudu** | **Dlula** |
| --- | --- | --- | --- | --- |
| 7.1 | Khetha isitatimende esikuhlathulula kuhle nasiqala iingozi ze-HIV | | | |
|  |  | |  |  |
| A | Nakanjani ngizokungenwa yi-HIV | | 1 |  |
| B | Mhlamunye kungenzeka ngingenwe yi- HIV | | 2 |  |
| C | Kungenzeka ngingangenwa yi-HIV | | 3 |  |
| D | Nakanjani angeze ngangenwa yi-HIV | | 4 |  |

| 7.2 | Ngiziphi iinzathu zakho ezenza bona ukholwe bona ungangenwa nanyana ANGEZE wangenwa yi-HIV?  (**Kilabo abaphendule u- 3 nanyana u- 4 embuzweni 7.1**) | | Akhange khengiye emsebeni ngaphambilini Ngilisile ukuya emsemeni Ngithembekile kumlinganami Ngethemba umlinganami Ngisebenzisa amakhondomu Ngazi ubujamo bakhe Ngazi ubujamo bomlinganami Angiyi emsemeni nabathengisi ngedini/abomarhotjha Ngivikelwe bezimu Ngivikelwe nguZimu  Angikho engozini ye-HIV Okhunye | | | | 1 2 3 4 5 6 7 8  9 10 11 12 | **Dlula kumbuzo7.3 bese uragela phambili ku 7.4** |
| --- | --- | --- | --- | --- | --- | --- | --- | --- |
| 7.3 | Ngiziphi iinzathu zakho ezenza bona ukholwe bona kungenzeka ungenwe nanyana nakanjani uzokungenwa yi-HIV?  (**Kilabo abaphendule ngo- 1 nanyana ngo 2 embuzweni 7.1**) | | | | | | | |
|  | **UMLAYEZO**: **UNGAFUNDELI AMAKHETHO LA PHEZULU. KUNGABA KHONA IIMPENDULO EZINENGI** | | Ngimajadu kwezomseme Benginabalingani abanengi Angiwasebenzisi amakhondomu Angiwasebenzisi amakhondomu kwesinye isikhathi Angimethembi umlinganami Ngiyagula Umlinganami uyagula Umlinganami wabulawa yi-AIDS Ngathola ingozi/Imisikeko Umlinganami u-HIV phosethivu Okhunye(itjho)______________________________ | | | | 1 2 3 4 5 6 7 8 9 10 11 |  |
| 7.4 | Ungakhona ukuzihlola wena ngokwakho nawunganikelwa isisetjenziswa sokuzihlola? | | | | Iye Awa Angazi | | 1 2 3 |  |
|  |  |  |  |  |  |  |  |  |

| **Q#** | **Umbuzo** | **Iimpendulo** | | | |
| --- | --- | --- | --- | --- | --- |
| 7.5 | **UMTLOLO:** Njenganje ngifuna ukukubuza imibuzo ngamakhondomu nangezokuthandana  1 = Angivumelani nakancani (SD) 2 = Angivumelani (D)  3 = Ngiyavumelana (A) 4 = Ngivumelana khulu (SA)) **UMLAYEZO : FUNDELA PHEZULU ZOKE IINTATIMENDE** | | | | |
|  |  | **SD** | **D** | **A** | **SA** |
| a | **(Amadoda)** Amadoda ayasaba bona abafazibabo/iintombizabo zizabalahla nange baphakamisa bona kusetjenziswe ikhondomu. | 1 | 2 | 3 | 4 |

| b | **(Abafazi)** Abafazi bayasaba bona amadodabo/amasokanabo azabalahla nange baphakamisa bona kusetjenziswe ikhondomu. | | | | | | | | 1 | | 2 | | | 3 | | 4 |
| --- | --- | --- | --- | --- | --- | --- | --- | --- | --- | --- | --- | --- | --- | --- | --- | --- |
| c | Nangabe umuntu ubhalelwa kufaka ikhondomu, uzakuba neenhloni ukobana ayisebenzise godu. | | | | | | | | 1 | | 2 | | | 3 | | 4 |
| d | Abomma abaziphathela amakhondomu batjheja amaphilwabo. | | | | | | | | 1 | | 2 | | | 3 | | 4 |
| e | Ukusebenzisa ikhondomu kuzakwenza umlinganakho acabange bona awumethembi. | | | | | | | | 1 | | 2 | | | 3 | | 4 |
| f | Lokha nawusebenzisa ikhondomu angeze waneliseka ngokuzwa ubumnandi ngokwaneleko. | | | | | | | | 1 | | 2 | | | 3 | | 4 |
| g | Nawukhulumisana kuhle nomlinganakho, unganeliseka kwezomseme ngomuntu oyedwa. | | | | | | | | 1 | | 2 | | | 3 | | 4 |
| h | Ngifuna omunye umuntu ukobana ngivale isikhundla nakungenzeka ngihlukane nomlinganami okunguye nguye. | | | | | | | | 1 | | 2 | | | 3 | | 4 |
| I | Vane nginande ngiya komunye umuntu ngaphandle komlinganami ngombana uyanganelisa kwezomseme. | | | | | | | | 1 | | 2 | | | 3 | | 4 |
| j | Ingabe kulungile ukuya emsemeni nabanye ikani lokha umlinganakho angeze akuthola lokho. | | | | | | | | 1 | | 2 | | | 3 | | 4 |
| k | Inengi labangani bami bazikhakhazisa ngokuthi bangaki abantu abaya nabo emsemeni. | | | | | | | | 1 | | 2 | | | 3 | | 4 |
| l | Amakhondomu enza kube mnandi khulu | | | | | | | | 1 | | 2 | | | 3 | | 4 |
| **Q#** | | **Umbuzo** | | | | **Iimpendulo** | | | **Ikhowudu** | | | | | **Dlula** | | |
| 7.6 | | Mangakhi amadoda emphakathinakho ocabanga bona aya emsemeni nabafazi abanengi? **[PHAKAMISA IZANDLA ZOMBILI UKUTJENGISA AMADODA ALI-10]** | | | | | | | _____ | | | | |  | | |
| 7.7 | | Wena ngokwakho, ucabanga bona isenzwesi sokobana amadoda aye emsemeni nabafazi abanengi sivumelekile nanyana asikavumeleki? | | | | | Kuvumelekile Akukavumeleki Angazi | | 1 2 3 | | | | |  | | |
| 7.8 | | bangakhi abafazi emphakathinakho ocabanga bona baya emsemeni namadoda amanengi [**PHAKAMISA IZANDLA ZOMBILI UKUTJENGISA AMADODA ALI-10]** | | | | | | | _____ | | | | |  | | |
| 7.9 | | Wena ngokwakho, ucabanga bona isenzwesi sokobana abafazi baye emsemeni namadoda amanengi sivumelekile nanyana asikavumeleki? | | | | | Kuvumelekile Akukavumeleki Angazi | | 1 2 3 | | | | |  | | |
|  | |  |  |  |  | |  | | | | |  |  | |  | |
| **Q#** | | **Umbuzo** | | | | | **Impendulo** | | | | | | | | | |
| 7.10 | | **UMTLOLO:** Uvumelana kngangani neentatimende ezilandelako ngawe?   1 = Angivumelani nakancani (SD)  2 = Angivumelani (D)   3 = Ngiyavumelana (A)  4 = Ngivumelana khulu (SA) **UMLAYEZO : FUNDELA PHEZULU ZOKE IINTATIMENDE** | | | | | | | | | | | | | | |
|  | |  | | | | | | **SD** | | **D** | | | **A** | | | **SA** |
| a | | Ngingayisebenzisa ikhondomu nalokha nangisele khulu. | | | | | | 1 | | 2 | | | 3 | | | 4 |
| b | | Ngingala ukuya emsemeni nangabe umuntu engimthandako uyabhala ukusebenzisa ikhondomu. | | | | | | 1 | | 2 | | | 3 | | | 4 |
| c | | Ngingawathenga amakhondomu ngaphandle kokuphathwa ziinhloni | | | | | | 1 | | 2 | | | 3 | | | 4 |
| d | | **[AMADODA)** Ngiyazithemba bona ngingayifaka kuhle ikhondomu. **[ABAFAZI]** Ngiyazithemba bona ngingayifaka kuhle ikhondomu endodeni nangiye nayo emsemeni | | | | | | 1 | | 2 | | | 3 | | | 4 |

| e | Vane ngingazi bona ngithini lokha sinomlinganami nasifuna ukukhuluma ngamakhondomu nanyana okhunye okuziimvikelo | 1 | 2 | 3 | 4 |
| --- | --- | --- | --- | --- | --- |
| f | Ngaso soke isikhathi vane ngizizwe ngingakakhululeki lokha nangithenga amakhondomu | 1 | 2 | 3 | 4 |

| **ISIGABA 8 : UKUSOKWA KWAMADODA** | | | | | | | | |
| --- | --- | --- | --- | --- | --- | --- | --- | --- |
|  |  |  |  |  |  |  |  |  |
| **UMTLOLO**: Njenganje ngizokubuza imibuzo embalwa mayelana nokusokwa kobudoda okungawe (emadodeni)/ ngomlinganakho (ebafazini) | | | | | | | | |

| **Q#** | **Umbuzo** | | **Impendulo** | **Ikhowudu** | **Dlula** |
| --- | --- | --- | --- | --- | --- |
| 8.1 | **(Amadoda)**: Usokiwe? (**Abafazi)**: Umlinganakho usokiwe? | | Iye Awa Anginapendulo | 1 2 3 | **Nangabe ipendulo ngu awa tjhinga kumbuzo 8.3,nangabe mumuntu wengubo tjhinga kusigaba 9** |
| 8.2 | Kubayini usokiwe? | | | | |
|  | **UMLAYEZO**: **UNGAFUNDELI AMAKHETHO LA PHEZULU. KUNGABA KHONA IIMPENDULO EZINENGI BUZA : Khuyini okhunye** | Ngezwa emrhatjhweni  Siqunto esathathwa babelethi bami Ngokwekambiso yesiko/yekolo ukukhandela i-HIV  Ukukhandela ama-STIs okhunye (itjho)______________________  Ngala ukuphendula | | 1 2 3 4 5 6 7 |  |
| 8.3 | Kubayini ungakasokwa? | | | | |
|  | **UMLAYEZO**: **UNGAFUNDELI AMAKHETHO LA PHEZULU. KUNGABA KHONA IIMPENDULO EZINENGI   BUZA : Yini okhunye** | Umuntu angazithola angasabelethi kwehlisa ubumnandi bomseme Anginasiqiniseko seenkambiso zokuphepha Abekho abangani bami abasokiweko Abangani bami bazangihleka Umlinganami akafuni Akusilo isiko lami Ngesaba ubuhlungu Angifuni ukuhlala ngingayi emsemeni isikhathi esingaba side khulu (iimveke ezisithandathu)  Okhunye (Itjho)__________________________________ Angifuni ukuphendula | | 1 2 3 4 5 6 7 8 9  10 11 |  |
| 8.4 | Ingabe utlama bona uyokusokwa eemvekeni ezilandelako ezili-12? | | Iye ngizakuya  Mhlamunye ngizakuya  Awa angeze ngiye  Iye angeze ngiye Anginasiqiniseko | 1 2 3 4 5 |  |

| **ISIGABA 9 : UKUVEZWA NGAPHAMBILI KWE-PROPHYLAXIS** | | | | | | | | |
| --- | --- | --- | --- | --- | --- | --- | --- | --- |
|  |  |  |  |  |  |  |  |  |
| **UMTLOLO:** Njenganje ngifuna ukukubuza ngokusetjenziswa kweenhlahla ukusiza ukukhandela ukungenwa yi-HIV. Khumbula ayikho ipendulo elungileko nengakalungi begodu yoke into oyitjhoko izakuba yifihlo. Nangabe awufuni ukuphendula umbuzo, ungadlulela embuzweni olandelako. | | | | | | | | |

| **Q#** | **Umbuzo** | | **Impendulo** | **Ikhowudu** | **Dlula** |
| --- | --- | --- | --- | --- | --- |
| 9.1 | Wakhe wezwa nge-Pre-exposure prophylaxis, ebizwa ngokuthiwa yi-PrEP? | | Iye Awa | 1 2 |  |
| 9.2 | Wakhe wezwa ngemitjhoga ye-HIV eselwako ukukusiza ukukhandela ukungenwa yi-HIV? | | Iye Awa | 1 2 |  |
| **ILWAZI:** I-PrEP ijamele i-Pre-Exposure Prophylaxis. Lipilisi eliselwa babantu aba-HIV negethivu woke malanga ukusiza ukukhandela bona bangabi HIV phosethivu. | | | | | |
| 9.3 | Ungaba nekareko lokusebenzisa i-PrEP? | | Iye Awa Anginasiqiniseko | 1 2 3 | **Tjhinga ku mbuzo 9.5 Tjhinga kumbuzo 9.6** |
| 9.4 | Unganyula ukufikelela njani ku-PrEP? [**Khetha kubili okuqakatheke khulu**] | | Udorhodera wangasese Isibhedlela Itlinigi  Ikhemisi Isikolo Iinsebenzi zezamaphilo Iinhlangano ezingasizo zombuso (ama-NGO) Inyanga yesintu Itlinigi kamakhambangendlwana Ilunga lomndeni Okhunye(itjho)___________________ | 1 2 3 4 5 6 7  8 9 10 11 | **nangabe kukhethwe kubili yeke, dlulela kumbuzo 9.6** |
| 9.5 | Angeze ngayisebenzisa i-PrEP ngombana: [**Khetha kubili okuqakatheke khulu**] | | | | |
|  |  | Amathelelwano wangeqadi angaba khona Angazi ngokwaneleko ngayo Angeze ngathanda bona abangani bami nomndenami bazi bona ngiyayisebenzisa Nangabe ngiyayisebenzisa ngizakuba majadu khulu kwezomseme  Angifuni ukusela ipilisi woke malanga Nangabe ngiyayisebenzisa ngizakuba nokuya emsemeni okunobungozi, njengokungasebenzisi ikhondomu Okhunye(itjho)_______________ | | 1 2 3  4  5 6  7 |  |
| 9.6 | Ngiziphi izinto ezintathu eziqakathekileko ongathanda ukuzazi nge-PrEP ukukusiza bona uthathe isiqunto nange ufuna ukuyisebenzisa? [**Khetha kuthathu okuqakatheke khulu**] | | | | |

|  |  | Amathelelwano wangeqadi Indawo engingathola kiyo ipilisi Umuntu onikela imitjhoga Isikhathi sokuthatha ipilisi Ipiliseli lisebenza kuhle kangangani Kufanele ngilithathe kangaki (kanye ngelanga ngaphambi kokuya emsemeni) Iindleko zepilisi Liselwa njani Okhunye (itjho) _________________________________ | 1 2 3 4 5 6  7 8 9 |  |
| --- | --- | --- | --- | --- |
| **ILWAZI:** Sizakuthanda bona wena usiyelelise ngokuthi ngiziphi iindlela ezihle ekufanele sikunikele ngazo ilwazi nge-PrEP wena nabangani bakho. | | | | |
| 9.6 | Kuzakuba ngiziphi iindlela ezihle zokutjela abantu abatjha emphakathinakho nge-PrEP? [**Khetha kuthathu okuqakatheke khulu**] | | | |
|  |  | Iphephandaba  Imikhangiso kumabhilibhodi  Amavakatjho wesikolo Imikhangiso ye-TV  Amabhrotjha anikelwa kunkghonakalisi zezamaphilo  Amahlelo wokuthintana afana neFacebook kanye ne-WhatsApp Okhunye(itjho)__________________________________ | 1 2 3 4 5 6 7 |  |

| **ISIGABA 10 : IIMBIKINDABA, UKUTHINTANA KANYE NEMIKGHWA** | | | | | | | | |
| --- | --- | --- | --- | --- | --- | --- | --- | --- |
|  |  |  |  |  |  |  |  |  |
| **UMTLOLO:**Njenganje ngizokubuza imibuzo eminengana ngemiithombo yelwazi ehlukeneko nokobana ucabanga ini ngayo | | | | | | | | |

| **Q#** | **Umbuzo** | **Iimpendulo** | | | | |
| --- | --- | --- | --- | --- | --- | --- |
| 10.1 | **UMTLOLO (SCRIPT):** Ukwenza kangaki lokhu okulandelako?   **1 = Angeze (N)  2 = Ngakanye/ ngaleso sikhathi (O)  3 = Kanye ngeveke (OW)  4 = Amalanga ama-2 ukuya kwasi-6 ngeveke (2DW)  5 = Qobe lilanga ngeveke (EW)** **UMLAYEZO : FUNDELA PHEZULU ZOKE IINTATIMENDE** | | | | | |
|  |  | **N** | **O** | **OW** | **2DW** | **EW** |
| a | Ukulalela umrhatjho | 1 | 2 | 3 | 4 | 5 |
| b | Ukubukela umabonakude | 1 | 2 | 3 | 4 | 5 |
| c | Ukufunda umagazini | 1 | 2 | 3 | 4 | 5 |
| d | Ukufunda iphephandaba | 1 | 2 | 3 | 4 | 5 |
| e | Ukusebenzisa i-inthanede ukobana uye emikhakheni yeendaba | 1 | 2 | 3 | 4 | 5 |
| f | Ukusebenzisa umaliledinini nanyana ikhomphyutha nanyana iTablet ukuya ku-Facebook | 1 | 2 | 3 | 4 | 5 |

| g | Ukusebenzisa umaliledinini nanyana ikhomphyutha nanyana iTablet ukuya kuThwitha | | | 1 | 2 | 3 | 4 | 5 |
| --- | --- | --- | --- | --- | --- | --- | --- | --- |
| h | Ukusebenzisa umaliledinini nanyana ikhomphyutha nanyana iTablet ukuya kumikhakha ye-HIV nanyana kweminye imikhakha yezamaphilo ekhambisana nazo | | | 1 | 2 | 3 | 4 | 5 |
| I | Ukusebenzisa umaliledinini nanyana i-inthanede ukuya ku-Instagremu | | | 1 | 2 | 3 | 4 | 5 |
| j | Ukusebenzisa umaliledinini ukuya ku- WhatsApp | | | 1 | 2 | 3 | 4 | 5 |
|  |  |  |  |  |  |  |  |  |
| **Q#** | **Umbuzo** | | | **Impendulo** | | | | |
| **Umtlolo:** Njenganje ngifuna ukukubuza imibuzo ngamatjhaneli owenyulako wemrhatjhi nanyana amakhasi **REDcap UMLAYEZO:MHLUNGI- BUZA KWAPHELA NANGABE IPENDULO NGEHLA NGU- 2, 3, 4 NANYANA 5** | | | | | | | | |
| 10.2 | Ngiziphi iintethji somrhatjho osilalela khulu?  **[Mvumele arhemise iintetjhi bezibe zi-3]** | | | 1 =________________ 2 =________________ 3 _________________ | | | | |
| 10.3 | Ngiziphi iintetjhi zakamabonakude ozibukela khulu?  **[Mvumele arhemise iintetjhi bezibe zi-3]** | | | 1 =________________ 2 =________________ 3 =_________________ | | | | |
| 10.4 | Ngiliphi iziko lokuthintana laku-inthanede olivakatjhela khulu? **[Allow list up to 3 websites]** | | | 1 =________________ 2 =________________ 3 =_________________ | | | | |
| 10.5 | Ngiliphi ikhasi lefacebook ongena kiwo kanengi? **[Mvumele arhemise amawebhusayisi aze abe ma-3]** | | | 1 =________________ 2 =________________ 3 =_________________ | | | | |
| 10.6 | Unyula ukukereya imilayeza malungana nokuvikelwa, ukuhlolwa kanye nokulatjhwa kwe HIV namkha i TB? Khetha…. | | | Umrhathjo  Iphepha ndaba  I-sms  i-WhatsApp amahlelo wezokuthintana njenge- Facebook, twitter  umabonakude  imagazini | | | 1  2  3  4  5  6  7 | |

Ufike emaphethelweni werherho lemibuzo yethu.sithokoze khulu bona uzibandakanye kule survey. Sithokoza bona uthathe isikhathi sakho.
