# Supplementary material for: Study on knowledge about associated factors of Tuberculosis (TB) and TB/HIV co-infection among young adults in two districts of South Africa
Source: PLoS One. 2019 Jun 6;14(6):e0217836. doi: 10.1371/journal.pone.0217836 (PMC6553726; doi:10.1371/journal.pone.0217836)
Supplement: S3 File — (PDF) [file pone.0217836.s003.pdf]

Foundation for Professional Development
Foundation for Professional Development

KAP Study

Codebook ▾

Data Dictionary Codebook

02-01-2018 12:57

#	Variable / Field Name	Field Label<br>*Field Note*	Field Attributes (Field Type, Validation, Choices, Calculations, etc.)
Instrument: **House Visits and Screening** (house_visits_and_screening)			
1	record_id	Record ID	text
2	screening_started_time	Section Header: *Section A: Staff Member Details*<br>Date and Time Started	text (datetime_dmy)<br>Custom alignment: LV<br>Field Annotation: @HIDEBUTTON @READONLY @NOW
3	research_assistant	Name of the Research Assistant	text<br>Field Annotation: @READONLY @USERNAME
4	province	Section Header: *Section B: Household Identification*<br>Province	radio, Required<br>1 Mpumalanga<br>2 Eastern Cape<br>Custom alignment: LV
5	district_nkangala_confirm<br>Show the field ONLY if:<br>[province] = '1'	Confirm the district:<br>Nkangala	radio, Required<br>1 Yes<br>Custom alignment: LV
6	district_or_tambo_confirm<br>Show the field ONLY if:<br>[province] = '2'	Confirm the district:<br>OR Tambo	radio, Required<br>1 Yes<br>Custom alignment: LV
7	area_eastern_cape<br>Show the field ONLY if:<br>[district_or_tambo_confirm] = '1'	Area	radio, Required<br>1 New Payne<br>2 Hillcrest<br>3 Jojweni<br>4 Blackway<br>5 Waterfall<br>6 Makhenkensi<br>7 Viedgesville

8	area_mpumalanga<br><br>Show the field ONLY if:<br>[district_nkangala_confirm] = '1'	Area	radio, Required<br>1 Matshipe<br>2 Kameelpoortnek B<br>3 Mathyzensloop<br>4 KwaMhlanga B<br>5 Phola Park<br>6 Moloto North<br>7 Thembalethu<br>8 Vezubuhle<br>9 Tweefontein A<br>10 Tweefontein K<br>11 Chris Hani<br>12 Tweefontein N<br>13 Langkloof<br>14 Kwaggafontein C<br>15 Gemsbokspruit<br><br>Custom alignment: LV
9	household_number<br><br>Show the field ONLY if:<br>[province] = '1' or [province] = '2'	Household Number/ Stand Number on the Map	text, Required<br>Custom alignment: LV
10	hh_visits<br><br>Show the field ONLY if:<br>[province] = '1' or [province] = '2'	Household Visits	checkbox, Required<br>1 hh_visits___1 First Household Visit<br>2 hh_visits___2 Second Household Visit<br>3 hh_visits___3 Third Household Visit
11	date_1st_visit<br><br>Show the field ONLY if:<br>[hh_visits(1)] = '1'	Section Header: *Details of the 1st household visit*<br>Date of the 1st household visit	text (date_dmy, Min: 2017-09-01, Max: 2018-02-01)
12	outcome_1st_hh_visit<br><br>Show the field ONLY if:<br>[hh_visits(1)] = '1'	Outcome of the 1st household visit	radio, Required<br>1 Someone present at home<br>2 No one present at home
13	person_present<br><br>Show the field ONLY if:<br>[outcome_1st_hh_visit] = '1'	Who is present at home?	radio, Required<br>1 Head of Household/Most Senior Member<br>2 Household Member (Not a Minor)<br>3 Household Member (Minor)

14	visit1_intro_script<br><br>Show the field ONLY if:<br>[person_present] = '1' or [person_present] = '2'	STUDY INTRODUCTION SCRIPT (ENGLISH)<br>Good day. My name is [research_assistant] and I am a Research Assistant from FPD. The organisation works with the DoH on various projects (TB/HIV/ANC) aimed at improving service provision in health facilities. We are conducting a study among youths between the ages 18-24 years; to assess the beliefs, attitudes and practices that different people in this community generally have about TB and HIV diseases. We would like to ask them to complete questions on the tablet about their knowledge, attitudes and practices about TB and HIV. Before we proceed, is there anyone between the ages 18-24 years that we can interview for the study?<br><br>STUDY INTRODUCTION SCRIPT (IsiXhosa)<br>Ndiyabulisa. Igama lam ndingu......................... ndingumncedisi kuphando lwakwa FPD. Eli gqiza lakwa FPD lisebenzisana ngamandla neSebe lezeMpilo kwinkonzo ezohlukeneyo ezinjenge sifo sephepha , intsholongwane kagawulayo kunye naba khulelweyo. Injongo kukuphuhlisa intsebenziswano kumasebe ezempilo. Siqulunqa esi sifundo kulutsha oluphakathi kweminyaka 18-24 yeminyaka ukuvavanya iinkolo, izimvo nengcingane kubantu abohlukeneyo ekuhlaleni jikelele malunga nesifo sephepha nentsholongwane ka gawulayo. Sinomnqweno wokucela ukuba baphendule le mibuzo ebuziweyo malunga ngolwazi lwabo nangokuqonda ngentsholongwane ka gawulayo nesifo sephepha. Phambi kokuba siqhubeke ingaba bakhona abantu abaphakathi kweminyaka eyi 18-24 esingabavavanyela esi sifundo?<br><br>STUDY INTRODUCTION SCRIPT (IsiNdebele)<br>Lothja ibizo lami ngingu______________ongumrhelebhi womrhubhululi e FPD. Okuyihlangano eberegisana nomyango wezamaphilo (DoH) kumaphrojethi ahlukahlukeneko (TB/HIV/ANC) anqophe ukuthuthukisa ukunikelwa kwensetjenziswa emazikweni wezamaphilo. Senza isifundwesi ebantwini abatjha abaphakathi kweminyaka kweminyaka engu 18 ukuya ku 24; ukuhlola iinkolelo, ukuziphatha kanye nendlela abantu abahlukahlukeneko emphakathini abaziphatha ngayo malungana namagulo afana ne sifo sofuba(TB) ne ntumbantonga(HIV). Singathanda ukubabawa bona baphendule imibuzo esiyiphethekho ku-tablet ngelwazi, ukuziphatha kanye nezenzo malungana ne TB ne HIV. Ngaphambi bona siragele phambili ingabe ukhona umuntu ophakathi kweminyaka engu 18 ukuya ku 24 esingamhlunga ngesifundwesi?	radio<br>1 | Read the study intro to the person<br><br>Custom alignment: LV

15	verbal_consent<br><br>Show the field ONLY if:<br>[person_present] = '1' or [person_present] = '2'	Can i get your permission to continue informing you more about the details of the study?	radio, Required<br>1 Yes<br>0 No
16	reason_not_interested<br><br>Show the field ONLY if:<br>[verbal_consent] = '0'	If no, can you please give a reason as to why you are not interested?	text, Required
17	end_1<br><br>Show the field ONLY if:<br>[verbal_consent] = '0'	THANK YOU VERY MUCH FOR YOUR TIME!	radio, Required<br>1 END<br><br>Custom alignment: RH
18	date_2nd_hh_visit<br><br>Show the field ONLY if:<br>[hh_visits(2)] = '1'	Section Header: *Details of the 2nd household visit*<br><br>Date of the second household visit	text (date_dmy, Min: 2017-09-01, Max: 2018-01-31), Required
19	outcome_2nd_hh_visit<br><br>Show the field ONLY if:<br>[hh_visits(2)] = '1'	Outcome of the 2nd household visit	radio, Required<br>1 Someone present at home<br>2 No one present at home
20	person_present_2<br><br>Show the field ONLY if:<br>[outcome_2nd_hh_visit] = '1'	Who is present at home?	radio, Required<br>1 Head of Household/Most Senior Member<br>2 Household Member (Not a Minor)<br>3 Household Member (Minor)

21	visit2_intro_script<br><br>Show the field ONLY if:<br>[person_present_2] = '1' or [person_present_2] = '2'	STUDY INTRODUCTION SCRIPT (ENGLISH)<br>Good day. My name is [research_assistant] and I am a Research Assistant from FPD. The organisation works with the DoH on various projects (TB/HIV/ANC) aimed at improving service provision in health facilities. We are conducting a study among youths between the ages 18-24 years; to assess the beliefs, attitudes and practices that different people in this community generally have about TB and HIV diseases. We would like to ask them to complete questions on the tablet about their knowledge, attitudes and practices about TB and HIV. Before we proceed, is there anyone between the ages 18-24 years that we can interview for the study?<br><br>STUDY INTRODUCTION SCRIPT (IsiXhosa)<br>Ndiyabulisa. Igama lam ndingu......................... ndingumncedisi kuphando lwakwa FPD. Eli gqiza lakwa FPD lisebenzisana ngamandla neSebe lezeMpilo kwinkonzo ezohlukeneyo ezinjenge sifo sephepha , intsholongwane kagawulayo kunye naba khulelweyo. Injongo kukuphuhlisa intsebenziswano kumasebe ezempilo. Siqulunqa esi sifundo kulutsha oluphakathi kweminyaka 18-24 yeminyaka ukuvavanya iinkolo, izimvo nengcingane kubantu abohlukeneyo ekuhlaleni jikelele malunga nesifo sephepha nentsholongwane ka gawulayo. Sinomnqweno wokucela ukuba baphendule le mibuzo ebuziweyo malunga ngolwazi lwabo nangokuqonda ngentsholongwane ka gawulayo nesifo sephepha. Phambi kokuba siqhubeke ingaba bakhona abantu abaphakathi kweminyaka eyi 18-24 esingabavavanyela esi sifundo?<br><br>STUDY INTRODUCTION SCRIPT (IsiNdebele)<br>Lothja ibizo lami ngingu_____________ongumrhelebhi womrhubhululi e FPD. Okuyihlangano eberegisana nomyango wezamaphilo (DoH) kumaphrojethi ahlukahlukeneko (TB/HIV/ANC) anqophe ukuthuthukisa ukunikelwa kwensetjenziswa emazikweni wezamaphilo. Senza isifundwesi ebantwini abatjha abaphakathi kweminyaka kweminyaka engu 18 ukuya ku 24; ukuhlola iinkolelo, ukuziphatha kanye nendlela abantu abahlukahlukeneko emphakathini abaziphatha ngayo malungana namagulo afana ne sifo sofuba(TB) ne ntumbantonga(HIV). Singathanda ukubabawa bona baphendule imibuzo esiyiphethekho ku-tablet ngelwazi, ukuziphatha kanye nezenzo malungana ne TB ne HIV. Ngaphambi bona siragele phambili ingabe ukhona umuntu ophakathi kweminyaka engu 18 ukuya ku 24 esingamhlunga ngesifundwesi?	radio<br>1 | Read the study intro to the person<br><br>Custom alignment: LV

22	verbal_consent_2<br><br>Show the field ONLY if:<br>[visit2_intro_script] = '1'	Can i get your permission to continue informing you more about the details of the study?	radio, Required<br>1 Yes<br>0 No
23	reason_not_interested_2<br><br>Show the field ONLY if:<br>[verbal_consent_2] = '0'	If no, can you please give a reason as to why you are not interested?	text, Required
24	end_2<br><br>Show the field ONLY if:<br>[verbal_consent_2] = '0'	THANK YOU VERY MUCH FOR YOUR TIME!	radio, Required<br>1 END<br><br>Custom alignment: RH
25	date_3rd_hh_visit<br><br>Show the field ONLY if:<br>[hh_visits(3)] = '1'	Section Header: *Details of the 3rd household visit*<br>Date of the third household visit	text (date_dmy, Min: 2017-09-01, Max: 2018-01-31), Required
26	outcome_3rd_hh_visit<br><br>Show the field ONLY if:<br>[hh_visits(3)] = '1'	Outcome of the 3rd household visit	radio, Required<br>1 Someone present at home<br>2 No one present at home
27	person_present_3<br><br>Show the field ONLY if:<br>[outcome_3rd_hh_visit] = '1'	Who is present at home?	radio, Required<br>1 Head of Household/Most Senior Member<br>2 Household Member (Not a Minor)<br>3 Household Member (Minor)

28	visit3_intro_script<br><br>Show the field ONLY if:<br>[person_present_3] = '1' or [person_present _3] = '2'	STUDY INTRODUCTION SCRIPT (ENGLISH)<br>Good day. My name is [research_assistant] and I am a Research Assistant from FPD. The organisation works with the DoH on various projects (TB/HIV/ANC) aimed at improving service provision in health facilities. We are conducting a study among youths between the ages 18-24 years; to assess the beliefs, attitudes and practices that different people in this community generally have about TB and HIV diseases. We would like to ask them to complete questions on the tablet about their knowledge, attitudes and practices about TB and HIV. Before we proceed, is there anyone between the ages 18-24 years that we can interview for the study?<br><br>STUDY INTRODUCTION SCRIPT (IsiXhosa)<br>Ndiyabulisa. Igama lam ndingu......................... ndingumncedisi kuphando lwakwa FPD. Eli gqiza lakwa FPD lisebenzisana ngamandla neSebe lezeMpilo kwinkonzo ezohlukeneyo ezinjenge sifo sephepha , intsholongwane kagawulayo kunye naba khulelweyo. Injongo kukuphuhlisa intsebenziswano kumasebe ezempilo. Siqulunqa esi sifundo kulutsha oluphakathi kweminyaka 18-24 yeminyaka ukuvavanya iinkolo, izimvo nengcingane kubantu abohlukeneyo ekuhlaleni jikelele malunga nesifo sephepha nentsholongwane ka gawulayo. Sinomnqweno wokucela ukuba baphendule le mibuzo ebuziweyo malunga ngolwazi lwabo nangokuqonda ngentsholongwane ka gawulayo nesifo sephepha. Phambi kokuba siqhubeke ingaba bakhona abantu abaphakathi kweminyaka eyi 18-24 esingabavavanyela esi sifundo?<br><br>STUDY INTRODUCTION SCRIPT (IsiNdebele)<br>Lothja ibizo lami ngingu______________ongumrhelebhi womrhubhululi e FPD. Okuyihlangano eberegisana nomyango wezamaphilo (DoH) kumaphrojethi ahlukahlukeneko (TB/HIV/ANC) anqophe ukuthuthukisa ukunikelwa kwensetjenziswa emazikweni wezamaphilo. Senza isifundwesi ebantwini abatjha abaphakathi kweminyaka kweminyaka engu 18 ukuya ku 24; ukuhlola iinkolelo, ukuziphatha kanye nendlela abantu abahlukahlukeneko emphakathini abaziphatha ngayo malungana namagulo afana ne sifo sofuba(TB) ne ntumbantonga(HIV). Singathanda ukubabawa bona baphendule imibuzo esiyiphethekho ku-tablet ngelwazi, ukuziphatha kanye nezenzo malungana ne TB ne HIV. Ngaphambi bona siragele phambili ingabe ukhona umuntu ophakathi kweminyaka engu 18 ukuya ku 24 esingamhlunga ngesifundwesi?	radio<br><br>1 Read the study intro to the person<br><br>Custom alignment: LV

29	verbal_consent_3<br><br>Show the field ONLY if:<br>[visit3_intro_script] = '1'	Can i get your permission to continue informing you more about the details of the study?	radio, Required<br>1 Yes<br>0 No
30	pot_study_participant<br><br>Show the field ONLY if:<br>[verbal_consent] = '1' or [verbal_consent_2] = '1' or [verbal_consent_3] = '1'	Section Header: *Section C: Study Participant Identification*<br><br>In this household, do you have any individual who might be a potential study participant.<br><br>Potential study participant should be:<br>18 to 24 years of age	radio, Required<br>1 Yes<br>0 No
31	pot_participant_eligible<br><br>Show the field ONLY if:<br>[pot_study_participant] = '1'	How many people in this household are eligible to partake in the study?<br>*Put the 'NUMBER" of participants in this specific household with an age range of 18 - 24 years.*	text (number), Required
32	pot_participant_home<br><br>Show the field ONLY if:<br>[pot_study_participant] = '1'	Is this potential study participant present at home at the moment?	radio, Required<br>1 Yes<br>0 No
33	head_of_hh_participant<br><br>Show the field ONLY if:<br>[pot_participant_home] = '1'	Thank you so much for your time. Can i request to speak to the potential participant about the study and check whether they are eligible to participate in the study?	radio, Required<br>1 Yes<br>0 No
34	head_hh_part_no<br><br>Show the field ONLY if:<br>[head_of_hh_participant] = '0'	Is there any specific reason why you are not comfortable with us speaking to the potential participant?	notes, Required
35	schedule_day<br><br>Show the field ONLY if:<br>[pot_participant_home] = '0'	Can i schedule a date which is convenient for me to come and find the potential study participant home?	radio, Required<br>1 Yes<br>0 No
36	name_surname<br><br>Show the field ONLY if:<br>[schedule_day] = '1' or [head_of_hh_participant] = '1'	Section Header: *Section D: Participant Details (Screening/Scheduling)*<br><br>Name and Surname of the Candidate<br>*Please put the name first, followed by the surname*	text, Required, Identifier
37	contact_1<br><br>Show the field ONLY if:<br>[schedule_day] = '1'	Section Header: *Scheduling*<br><br>Preferred Contact Number	text (number, Min: 0111111111, Max: 0999999999), Required

38	contact_2<br><br>Show the field ONLY if:<br>[schedule_day] = '1'	Additional Contact Number<br>*leave blank if there are no additional contacts*	text (number, Min: 0111111111, Max: 0999999999)
39	schedule_date_return<br><br>Show the field ONLY if:<br>[schedule_day] = '1'	Which date can i come back to the house?	text (date_dmy, Min: 2017-09-01, Max: 2018-01-31), Required
40	schedule_time_return<br><br>Show the field ONLY if:<br>[schedule_day] = '1'	Please specify the appropriate time	radio, Required<br>1 | Morning (7am - 11h59)<br>2 | Early Afternoon (12h00 - 13h59)<br>3 | Late Afternoon (14h00 - 16h30)
41	participant_intro_script<br><br>Show the field ONLY if:<br>[head_of_hh_participant] = '1'	Section Header: *Screening*<br>Potential Participant Script:<br><br>Good day. My name is [research_assistant] and I am a Research Assistant from FPD. The organisation works with the DoH on various projects (TB/HIV/ANC) aimed at improving service provision in health facilities. We are conducting a study among youths between the ages 18-24 years; to assess the beliefs, attitudes and practices that different people in this community generally have about TB and HIV diseases. We would like to ask you to complete questions on the tablet about your knowledge, attitudes and practices about TB and HIV. Will it be okay? Before we proceed, may I please ask you a few questions to check if you qualify to participate in the study?<br><br>Potential Participant Script (IsiXhosa)<br><br>Ndiyabulisa. Igama lam ndingu......................... ndingumncedisi kuphando lwakwa FPD. Eli gqiza lakwa FPD lisebenzisana ngamandla neSebe lezeMpilo kwinkonzo ezohlukeneyo ezinjenge sifo sephepha , intsholongwane kagawulayo kunye naba khulelweyo. Injongo kukuphuhlisa intsebenziswano kumasebe ezempilo. Siqulunqa esi sifundo kulutsha oluphakathi kweminyaka 18-24 yeminyaka ukuvavanya iinkolo, izimvo nengcingane kubantu abohlukeneyo ekuhlaleni jikelele malunga nesifo sephepha nentsholongwane ka gawulayo. Sinomnqweno wokubacela ukuba baphendule le mibuzo ebuziweyo malunga ngolwazi lwabo nangokuqonda ngentsholongwane ka gawulayo nesifo sephepha. Ingaba kulungile? Phambi kokuba siqhubekeke ndicela ukukubuza imibuzo embalwa ukuze sijonge ukuba ukulungele na ukuphononongwa nokuthatha inxaxheba kwesisifundo<br><br>Potential Participant Script (IsiNdebele)	radio, Required<br>1 | Read the introduction script to the potential participant<br><br>Custom alignment: LV

		Lothja ibizo lami ngingu_____________ongumrhelebhi womrhubhululi e FPD. Okuyihlangano eberegisana nomyango wezamaphilo (DoH) kumaphrojethi ahlukahlukeneko (TB/HIV/ANC) anqophe ukuthuthukisa ukunikelwa kwensetjenziswa emazikweni wezamaphilo. Senza isifundwesi ebantwini abatjha abaphakathi kweminyaka kweminyaka engu 18 ukuya ku 24; ukuhlola iinkolelo, ukuziphatha kanye nendlela abantu abahlukahlukeneko emphakathini abaziphatha ngayo malungana namagulo afana ne sifo sofuba(TB) ne ntumbantonga(HIV). Singathanda bona uphendule imibuzo esiyiphetheko ku-tablet ngelwazi, ukuziphatha kanye nezenzo malungana ne TB ne HIV. Ingabe lokho kulungile? Ngaphambi kobana sithome ngibawa ukukubuza imibuzo embalwa ukuhlola bona ukulungele ukuzibandakanya esifundweni lesi?	
42	screening<br><br>Show the field ONLY if:<br>[head_of_hh_participant] = '1'	I will now ask you a couple of questions, the purpose of these questions is to see if you are eligible to participate in the study.<br><br>Do you give me the permission to continue?	radio, Required<br>1 Yes<br>0 No
43	dob<br><br>Show the field ONLY if:<br>[screening] = '1'	What is your date of birth?	text (date_dmy), Required<br>Field Annotation: @HIDEBUTTON
44	auto_age<br><br>Show the field ONLY if:<br>[screening] = '1'	RA: Auto-calculated Age<br>*Compare the auto-calculated date with the age you obtained from the participant*	calc<br>Calculation:<br>round(datediff([dob],'today','y','dmy'),0)
45	age<br><br>Show the field ONLY if:<br>[screening] = '1'	How old are you?<br>*YEARS*	text (number, Min: 6, Max: 100), Required
46	screening_refuse<br><br>Show the field ONLY if:<br>[screening] = '0'	Why are you refusing to partake in an eligibility screening process?	text, Required
47	gender<br><br>Show the field ONLY if:<br>[screening] = '1'	RA: Select the Gender	radio, Required<br>1 Male<br>2 Female

48	language<br><br>Show the field ONLY if:<br>[screening] = '1'	Which language would you prefer use for this study?<br><br>The participant should be able to write and read the language!	radio, Required<br>1 English<br>2 Xhosa<br>3 Ndebele<br>4 None
49	duration_stay<br><br>Show the field ONLY if:<br>[screening] = '1'	How long have you been staying in this area?	radio, Required<br>1 Greater than 6 months<br>0 Less than 6 months
50	eligible_yes<br><br>Show the field ONLY if:<br>[duration_stay] = '1' and ([language] = '1' or [language] = '2' or [language] = '3') and [age] >= 18 and [age] <= 24	The participant is "ELIGIBLE" to partake in the study.<br><br>Please continue with the literacy assessment.	radio, Required<br>1 Yes
51	eligible_no<br><br>Show the field ONLY if:<br>[duration_stay] = '0' or [language] = '4' or ([age]<=17 and [age]>=25)	The participant is "NOT ELIGIBLE" to partake in the study.<br><br>Please thank them for their time and continue to the next household.	radio, Required<br>1 Yes
52	literacy_english<br><br>Show the field ONLY if:<br>[language] = '1' and [eligible_yes] = '1'	Literacy Assessment: English<br><br>Please read aloud the text in BLUE color below:	checkbox<br>1 literacy_english___1 Many young people are not aware of their HIV status even if they are at increased HIV risk in South Africa .<br>2 literacy_english___2 The research aims to investigate young people's knowledge, attitudes, beliefs and practices<br>3 literacy_english___3 This project will take place over a period of six months<br><br>Custom alignment: LV

53	literacy_isixhosa<br><br>Show the field ONLY if:<br>[eligible_yes] = '1' and [language] = '2'	Literacy Assessment: IsiXhosa<br><br>Please read aloud the text in BLUE color below:	checkbox<br>1 literacy_isixhosa___1 Abantu abaninz abatsha abaqor ngesimo sabo sentsholongwar kagawulayo nangona besemngciphek wokonyuka kwezinga lwale ntsholongwane kagawulayo kwi lizwekazi uMzar Afrika<br>2 literacy_isixhosa___2 Injongo yoluphononong kukuphanda malunga nolwa kubantu abatsh iinkolo, izimvo nendlela abenza ngayo izinto<br>3 literacy_isixhosa___3 Olu fundo luyakuthatha ix elingaphezulu kweenyanga ezintandathu<br><br>Custom alignment: LV

54	literacy_isindebele<br><br>Show the field ONLY if:<br>[eligible_yes] = '1' and [language] = '3'	Literacy Assessment: IsiNdebele<br><br>Please read aloud the text in BLUE color below:	checkbox<br>1 | literacy_isindebele___1 | Inengi labant abatjha alibaz ubujamo bab HIV nalokha basengozini kangaka yokungezeka kwabantu ab HIV eSewula Afrika<br>2 | literacy_isindebele___2 | Iphrojekthi le, yeke, ifuna ukuthoma irhubhululo u sizwisise ngconywana indlela abant bengubo nabembaji abasesebatjh abanelwazi ng abacabanga nabaziphatha ngayo ngeen ze-HIV ne-AID<br>3 | literacy_isindebele___3 | Iphrojekthi le izakuthatha isikhathi esingangeeny ezisithandath<br><br>Custom alignment: LV
55	literacy_outcome<br><br>Show the field ONLY if:<br>[eligible_yes] = '1'	Literacy Outcome	radio, Required<br>1 | Successful<br>0 | Unsuccessful
56	literacy_fail<br><br>Show the field ONLY if:<br>[literacy_outcome] = '0'	You are confirming that the participant failed the literacy assessment.<br><br>Please thank them for their time and move to the next household.	radio, Required<br>1 | END
57	continue_consent<br><br>Show the field ONLY if:<br>[literacy_outcome] = '1'	The Participant is Eligible and Passed the Literacy Assessment.<br><br>Please Initiate The Consenting Process	radio, Required<br>1 | Continue
58	consent<br><br>Show the field ONLY if:<br>[continue_consent] = '1'	Section Header: *Section E: Consenting*<br>Informed consent	radio, Required<br>1 | Yes - Obtained<br>0 | No - Willing to do a post-refusal questionnaire<br>2 | No - Not willing to do the post-refusal questionnaire

59	consent_copy_given<br><br>Show the field ONLY if:<br>[consent] = '1'	Did the participant get a COPY of an informed consent document?	radio, Required<br>1 Yes<br>0 No
60	end3<br><br>Show the field ONLY if:<br>[consent] = '2'	THANK YOU SO MUCH FOR YOUR TIME!	radio, Required<br>1 END
61	consent_refusal<br><br>Show the field ONLY if:<br>[consent] = '0'	Section Header: *Post-Refusal Questionnaire*<br><br>Why do you not want to participate in the study?	checkbox, Required<br>1 consent_refusal___1 I am not interested<br>2 consent_refusal___2 I am enrolled in another study<br>3 consent_refusal___3 I do not have time<br>4 consent_refusal___4 I am too stressed out<br>5 consent_refusal___5 My parent(s) or Household Head won t allow me to<br>6 consent_refusal___6 The house is not the best place to do the interview<br>7 consent_refusal___7 I am not feeling well<br>8 consent_refusal___8 Other
62	consent_refusal_other<br><br>Show the field ONLY if:<br>[consent_refusal(8)] = '1'	If other, please specify	text, Required
63	refuse_race<br><br>Show the field ONLY if:<br>[consent] = '0'	How would you describe yourself in terms of race?	radio, Required<br>1 African<br>2 Coloured<br>3 White<br>4 Asian

64	refuse_level_education<br><br>Show the field ONLY if:<br>[consent] = '0'	What level of education did you complete?	radio, Required<br>1 None<br>2 Below Matric<br>3 Matric<br>4 Degree/Diploma
65	refuse_marital_status<br><br>Show the field ONLY if:<br>[consent] = '0'	How would you describe your relationship (marital) status?	radio, Required<br>1 Single<br>2 Married<br>3 Divorced<br>4 Steady Partner living with me<br>5 Steady Partner not living with me
66	refuse_employment<br><br>Show the field ONLY if:<br>[consent] = '0'	Are you currently employed?	radio, Required<br>1 Employed full time<br>2 Employed part time<br>3 Self Employed<br>4 Student<br>5 Not employed
67	refuse_money<br><br>Show the field ONLY if:<br>[consent] = '0'	Where do you get money to meet your basic monthly needs?	radio, Required<br>1 Social grant<br>2 Salary/Wages from employer<br>3 Business profit<br>4 Partner/Spouse<br>5 Family
68	refuse_born_sa<br><br>Show the field ONLY if:<br>[consent] = '0'	Were you born in South Africa?	radio, Required<br>1 Yes<br>0 No
69	refuse_country<br><br>Show the field ONLY if:<br>[refuse_born_sa] = '0'	In which country were you born?	text, Required
70	refuse_year<br><br>Show the field ONLY if:<br>[refuse_born_sa] = '0'	What year did you come to South Africa?	text (number, Min: 1001, Max: 2017), Required
71	participant_pin<br><br>Show the field ONLY if:<br>[consent] = '1'	Section Header: *Enrolment Details*<br>Participant Pin on the Hard Copy Enrolment Log<br>*PUT THE PIN AS IT APPEARS ON THE ENROLMENT LOG!!!*	text, Required<br>Field Annotation: @PLACEHOLDER="An example: M-0001" @CHARLIMIT='6'
72	screen_comments	Any Comments	notes

73	house_visits_and_screening_complete	Section Header: *Form Status*<br>Complete?	dropdown<br>0 Incomplete<br>1 Unverified<br>2 Complete
Instrument: **Practicing Questions** (practicing_questions)			
74	pract_number<br>Show the field ONLY if:<br>[language] = '1' and [consent] = '1'	Section Header: *Section A: Test Questions and Responses This section should be completed by the PARTICIPANT with the help of the RESEARCH ASSISTANT. The main purpose of this section is to familiarise the participant with the use of the tablet and how to respond to various question types.*<br>What is your favourite number?<br><br>Number should be from 0 to 100	text (number, Min: 0, Max: 100), Required
75	x_pract_number<br>Show the field ONLY if:<br>[consent] = '1' and [language] = '2'	Yeyiphi eyona nombolo uyithandayo?<br><br>Inombolo fanele iqale ku 0 ukuya ku 100?	text (number, Min: 0, Max: 100), Required
76	n_pract_number_2<br>Show the field ONLY if:<br>[consent] = '1' and [language] = '3'	Ngiyiphi inomboro oyithandako<br><br>Inomboro kufanele bona ithome ku 0 kuya ku 100	text (number, Min: 0, Max: 100), Required
77	pract_like_music<br>Show the field ONLY if:<br>[language] = '1' and [consent] = '1'	Do you enjoy listening to music?	radio, Required<br>1 Yes<br>0 No
78	x_pract_like_music<br>Show the field ONLY if:<br>[consent] = '1' and [language] = '2'	Ingaba uyakuthanda ukuphulaphula umculo	radio, Required<br>1 Ewe<br>0 Hayi
79	n_pract_like_music<br>Show the field ONLY if:<br>[consent] = '1' and [language] = '3'	Ingabe uyathanda ukulalela umvumo?	radio, Required<br>1 Iye<br>0 Awa
80	pract_today_date<br>Show the field ONLY if:<br>[language] = '1' and [consent] = '1'	What is today's date?	text (date_dmy), Required<br>Field Annotation: @HIDEBUTTON
81	x_pract_today_date<br>Show the field ONLY if:<br>[consent] = '1' and [language] = '2'	Ngumhla wesingaphi namhlanje?	text (date_dmy), Required<br>Field Annotation: @HIDEBUTTON

82	n_pract_today_date<br><br>Show the field ONLY if:<br>[consent] = '1' and [language] = '3'	Kuzingaki namhlanjesi?	text (date_dmy), Required<br>Field Annotation: @HIDEBUTTON
83	pract_food_like<br><br>Show the field ONLY if:<br>[language] = '1' and [consent] = '1'	Which of the following foods do you like?<br><br>You can select more than one	checkbox, Required<br>1 pract_food_like___1 Potatoes<br>2 pract_food_like___2 Pap<br>3 pract_food_like___3 Rice<br>4 pract_food_like___4 Sweet Potatoes<br>5 pract_food_like___5 Other<br>6 pract_food_like___6 None<br><br>Field Annotation: @NONEOFTHEABOVE='6'
84	x_pract_food_like<br><br>Show the field ONLY if:<br>[consent] = '1' and [language] = '2'	Kokuphi ukutya okuthandayo koku?	checkbox, Required<br>1 x_pract_food_like___1 Iitapile<br>2 x_pract_food_like___2 Ipapa<br>3 x_pract_food_like___3 Irice<br>4 x_pract_food_like___4 Ibhatata<br>5 x_pract_food_like___5 Okunye<br>6 x_pract_food_like___6 Akukho
85	n_pract_food_like<br><br>Show the field ONLY if:<br>[consent] = '1' and [language] = '3'	Ngikuphi ukudla okuthandako kilokhu okulandelako?	checkbox, Required<br>1 n_pract_food_like___1 Amazambane<br>2 n_pract_food_like___2 Umrata<br>3 n_pract_food_like___3 I-Rice<br>4 n_pract_food_like___4 Ibhatata<br>5 n_pract_food_like___5 Okhunye<br>6 n_pract_food_like___6 Akukho
86	pract_other<br><br>Show the field ONLY if:<br>[pract_food_like(5)] = '1'	If other, please specify	text, Required
87	x_pract_other<br><br>Show the field ONLY if:<br>[x_pract_food_like(5)] = '1'	Ukuba kukhona okunye, ndicela undazise	text, Required
88	n_pract_other<br><br>Show the field ONLY if:<br>[n_pract_food_like(5)] = '1'	Nangabe okhunye hlathulula	text, Required

89	practicing_questions_complete	Section Header: *Form Status*<br>Complete?	dropdown<br>0 Incomplete<br>1 Unverified<br>2 Complete
Instrument: **Electronic Questionnaire** (electronic_questionnaire)			
90	q1_1<br>Show the field ONLY if:<br>[language] = '1' and [consent] = '1'	Section Header: *SECTION 1: DEMOGRAPHICS*<br>How old were you on your last birthday? (Age of the respondent)	text (number)
91	q1_2<br>Show the field ONLY if:<br>[language] = '1' and [consent] = '1'	What is your date of birth? (DD)/MMM YYYY	text (date_dmy)<br>Field Annotation: @HIDEBUTTON
92	q1_3<br>Show the field ONLY if:<br>[language] = '1' and [consent] = '1'	How long have you lived here?	radio<br>1 Less than 4 weeks<br>2 More than a month, but less than 6 months<br>3 More than 6 months but less than a year<br>4 More than 1 year, but less than 5 years.<br>5 More than 5 years.<br>6 All my life.<br><br>Custom alignment: LV
93	q1_4<br>Show the field ONLY if:<br>[language] = '1' and [consent] = '1'	What is your gender?	radio<br>1 Male<br>2 Female<br><br>Custom alignment: LV<br>Question number: Q21

94	q1_5<br>Show the field ONLY if:<br>[language] = '1' and [consent] = '1'	What is your current marital status? (Marital status referring to legal, traditional or common-law)	radio<br>1 single<br>2 Not married or living together, but in a steady sexual relationship lasting more than 3 months<br>3 Not married, but living with sexual partner/boyfriend/girlfriend<br>4 Married, living with spouse<br>5 Married, but NOT living with spouse<br>6 Divorced/Widowed<br>7 Other(specify)<br>Custom alignment: LV<br>Question number: Q22
95	q1_5_oth<br>Show the field ONLY if:<br>[q1_5] = '7'	Other specify	notes<br>Custom alignment: LV
96	q1_6<br>Show the field ONLY if:<br>[language] = '1' and [consent] = '1'	How would you describe your present employment situation?	radio, Required<br>1 Housewife, homemaker, not looking for work<br>2 Housewife, homemaker, looking for work<br>3 Unemployed, looking for work<br>4 Unemployed, not looking for work<br>5 Work in informal sector, looking for permanent work<br>6 Work in informal sector, not looking for permanent work<br>7 Sick/disabled and unable to work<br>8 Student/pupil/learner<br>9 Self-employed - full time (40 hours or more per week)<br>10 Self-employed - part time (less than 40 hours per week)<br>11 Employed part time (if none of the above) (less than 40 hours per week)<br>12 Employed full time (40 hours or more per week)<br>13 Other<br>Custom alignment: LV<br>Question number: Q24

97	q1_6_oth<br><br>Show the field ONLY if:<br>[q1_6] = '13'	Other specify	notes<br>Custom alignment: LV
98	q1_7<br><br>Show the field ONLY if:<br>[language] = '1' and [c onsent] = '1'	What is your highest level of education that you completed?	radio<br>1 No schooling<br>2 Grade 1/Sub a/Class 1<br>3 Grade 2/Sub b/Class 2<br>4 Grade 3/Standard 1<br>5 Grade 4 /Standard 2<br>6 Grade 5 /Standard 3<br>7 Grade 6 /Standard 4<br>8 Grade 7/Standard 5<br>9 Grade 8 /Standard 6<br>10 Grade 9 /Standard 7<br>11 Grade 10/Standard 8<br>12 Grade 11/Standard 9<br>13 Grade 12/Standard 10/ Matric<br>14 Further studies incomplete<br>15 Certificate course completed after school (Grade 12/Matric)<br>16 Diploma/Degree post school completed<br>17 Further degree completed<br><br>Custom alignment: LV<br>Question number: Q26
99	q1_8<br><br>Show the field ONLY if:<br>[language] = '1' and [c onsent] = '1'	In the last 12 months, did you or other adults in your household have to reduce the amount of food?	radio<br>1 Yes<br>2 No<br><br>Custom alignment: LV<br>Question number: Q27
100	q1_9<br><br>Show the field ONLY if:<br>[q1_8] = '1'	In the last 12 months, were you ever hungry but did not eat food?	radio<br>1 Yes<br>2 No<br><br>Custom alignment: LV<br>Question number: Q28

101	q1_9a<br><br>Show the field ONLY if:<br>[q1_8] = '1' or [q1_9] = '1'	Q1.9a : What was the reason for reducing the amount of food or being hungry but did not eat?	radio<br>1 No food<br>2 No money to buy food<br>3 Dieting<br>4 Other reason<br><br>Custom alignment: LV
102	q1_9a_other_specify<br><br>Show the field ONLY if:<br>[q1_9a] = '4'	Q1.9a : Other specify	notes<br>Custom alignment: LV
103	q1_10<br><br>Show the field ONLY if:<br>[language] = '1' and [consent] = '1'	Which of the following best describes your house?	radio<br>1 Brick house<br>2 Flat<br>3 Wendy house/Backyard(zozo)/Bungalow<br>4 Shack (Mkhukhu)<br><br>Custom alignment: LV<br>Question number: Q30
104	q1_11<br><br>Show the field ONLY if:<br>[language] = '1' and [consent] = '1'	Which of these do you have in your house?<br>*[MULTIPLE RESPONSES ALLOWED]*	checkbox<br>1 q1_11___1 Electricity<br>2 q1_11___2 Radio<br>3 q1_11___3 TV<br>4 q1_11___4 Cellphone<br>5 q1_11___5 Refrigerator<br>6 q1_11___6 Don't remember<br>7 q1_11___7 Bicycle<br>8 q1_11___8 Motorcycle<br>9 q1_11___9 Car / truck / van<br>10 q1_11___10 Food Garden<br>11 q1_11___11 Livestock (like chickens)<br>12 q1_11___12 Don't remember<br>13 q1_11___13 Refused / No response<br><br>Custom alignment: LV<br>Field Annotation: @NONEOFTHEABOVE = '13'

105	q1_12<br>Show the field ONLY if:<br>[language] = '1' and [consent] = '1'	Are you a member of any clubs or groups or societies (e.g. soccer club, church, youth club, savings club)?	radio<br>1 Yes, a member<br>2 Yes, an active member<br>3 No<br>Custom alignment: LV<br>Question number: Q32
106	q1_13<br>Show the field ONLY if:<br>[language] = '1' and [consent] = '1'	Do you receive a social grant (e.g. child support grant, disability grant, etc.)	radio<br>1 Yes<br>2 No<br>Custom alignment: LV
107	q1_14<br>Show the field ONLY if:<br>[language] = '1' and [consent] = '1'	How do you describe yourself in terms of race? What is your race?	radio<br>1 White<br>2 Black African<br>3 Indian/Asian<br>4 Coloured<br>5 Other, Specify<br>Custom alignment: LV<br>Question number: Q34
108	q1_14_oth<br>Show the field ONLY if:<br>[q1_14] = '5'	Other race	notes<br>Custom alignment: LV
109	q1_15<br>Show the field ONLY if:<br>[language] = '1' and [consent] = '1'	Where do you get most of your money to meet your basic monthly needs?	radio<br>1 Social grant<br>2 Salary/Wages from employer<br>3 Business profit<br>4 Partner/spouse<br>5 Family<br>6 "Blesser"/ "Sugar Daddy"<br>7 Other, specify<br>Custom alignment: LV<br>Question number: Q36
110	q1_15oth<br>Show the field ONLY if:<br>[q1_15] = '7'	Q1:15 : Other, specify	notes<br>Custom alignment: LV
111	q2_1aa<br>Show the field ONLY if:<br>[language] = '1' and [consent] = '1'	Section Header: *SECTION 2 : KNOWLEDGE AND PERCEPTIONS OF HIV*<br>To prevent HIV infection, a condom must be used for every round of sex	radio<br>1 Yes<br>2 No<br>Custom alignment: LV

112	q2_1ab<br><br>Show the field ONLY if:<br>[language] = '1' and [consent] = '1'	One can reduce the risk of HIV by having fewer sexual partners	radio<br>1 Yes<br>2 No<br><br>Custom alignment: LV
113	q2_1ac<br><br>Show the field ONLY if:<br>[language] = '1' and [consent] = '1'	Can a healthy-looking person have HIV?	radio<br>1 Yes<br>2 No<br><br>Custom alignment: LV<br>Question number: Q39
114	q2_1ad<br><br>Show the field ONLY if:<br>[language] = '1' and [consent] = '1'	Can AIDS be cured?	radio<br>1 Yes<br>2 No<br><br>Custom alignment: LV<br>Question number: Q40
115	q2_1ae<br><br>Show the field ONLY if:<br>[language] = '1' and [consent] = '1'	Can a person get HIV by sharing food with someone who is infected?	radio<br>1 Yes<br>2 No<br><br>Custom alignment: LV<br>Question number: Q41
116	q2_1af<br><br>Show the field ONLY if:<br>[language] = '1' and [consent] = '1'	Can HIV be treated?	radio<br>1 Yes<br>2 No<br><br>Custom alignment: LV<br>Question number: Q42

<table>
<tr><td>117</td><td>q2_1b

Show the field ONLY if:
[language] = '1' and [consent] = '1'</td><td>Do you know how HIV can be prevented?
[MULTIPLE RESPONSES ALLOWED]</td><td>checkbox
<table><tr><td>1</td><td>q2_1b___1</td><td>It can't be prevented</td></tr><tr><td>2</td><td>q2_1b___2</td><td>Using condoms</td></tr><tr><td>3</td><td>q2_1b___3</td><td>Sticking to one sex partner</td></tr><tr><td>4</td><td>q2_1b___4</td><td>Being faithful to one sex partner who is also faithful to you</td></tr><tr><td>5</td><td>q2_1b___5</td><td>Reducing number of sex partners</td></tr><tr><td>6</td><td>q2_1b___6</td><td>Abstaining from sex</td></tr><tr><td>7</td><td>q2_1b___7</td><td>Avoiding contact with blood</td></tr><tr><td>8</td><td>q2_1b___8</td><td>Using drugs to prevent the mother giving the child HIV</td></tr><tr><td>9</td><td>q2_1b___9</td><td>Male circumcision</td></tr><tr><td>10</td><td>q2_1b___10</td><td>Microbicides (gel/ring inserted into the vagina to prevent HIV infection)</td></tr><tr><td>11</td><td>q2_1b___11</td><td>Pre-exposure prophylaxis (PrEP)</td></tr><tr><td>12</td><td>q2_1b___12</td><td>Post-exposure prophylaxis (PEP)</td></tr><tr><td>13</td><td>q2_1b___13</td><td>Other (specify)</td></tr><tr><td>14</td><td>q2_1b___14</td><td>I don't know</td></tr></table>
Custom alignment: LV
Field Annotation: @NONEOFTHEABOVE = '14'</td></tr>
<tr><td>118</td><td>q2_1b_oth

Show the field ONLY if:
[q2_1b(13)] = '1'</td><td>Other specify</td><td>notes
Custom alignment: LV</td></tr>
<tr><td>119</td><td>q2_2

Show the field ONLY if:
[language] = '1' and [consent] = '1'</td><td>Is there a treatment for HIV?</td><td>radio
<table><tr><td>1</td><td>Yes</td></tr><tr><td>2</td><td>No</td></tr><tr><td>3</td><td>Don't know</td></tr></table>
Custom alignment: LV
Question number: Q44</td></tr>
</table>

120	q2_3<br><br>Show the field ONLY if:<br>[q2_2] = '1'	What is that treatment?<br>*[MULTIPLE RESPONSES ALLOWED]*	checkbox<br>1 | q2_3___1 | Antiretroviral drugs/treatment (ARVs/ART)<br>2 | q2_3___2 | Other drugs, medicine, pills<br>3 | q2_3___3 | Traditional medicine<br>4 | q2_3___4 | Homeopathic treatment<br>5 | q2_3___5 | Immune boosters<br>6 | q2_3___6 | Prayers<br>7 | q2_3___7 | Other (specify)<br>8 | q2_3___8 | Don't know<br><br>Custom alignment: LV<br>Field Annotation: @NONEOFTHEABOVE = '8'
121	q2_3oth<br><br>Show the field ONLY if:<br>[q2_3(7)] = '1'	Q2.3 : Other, specify	notes<br>Custom alignment: LV
122	q2_4<br><br>Show the field ONLY if:<br>[q2_2] = '1'	How soon after testing HIV positive, can a person start treatment for HIV?<br>*[MULTIPLE RESPONSES ALLOWED]*	checkbox<br>1 | q2_4___1 | Immediately<br>2 | q2_4___2 | When the CD4 count is low (below 500)<br>3 | q2_4___3 | Immediately if the person is a pregnant woman<br>4 | q2_4___4 | Immediately if the person has TB<br>5 | q2_4___5 | Whenever the person is ready to start treatment<br>6 | q2_4___6 | Other (specify)<br>7 | q2_4___7 | Don't Know<br><br>Custom alignment: LV<br>Field Annotation: @NONEOFTHEABOVE = '7'
123	q2_4oth<br><br>Show the field ONLY if:<br>[q2_3(7)] = '1'	Other specify	notes<br>Custom alignment: LV

124	q2_6<br><br>Show the field ONLY if:<br>[q2_2] = '1'	How long do people have to stay on that treatment for HIV?	radio<br>1 For the rest of their lives.<br>2 As long as they want.<br>3 Until they feel better.<br>4 Until they are cured,<br>5 Don't know.<br>6 Other (specify)<br><br>Custom alignment: LV<br>Question number: Q47
125	q2_6oth<br><br>Show the field ONLY if:<br>[q2_6] = '6'	Q2.6 : other specify	notes<br>Custom alignment: LV
126	q2_7_1<br><br>Show the field ONLY if:<br>[language] = '1' and [consent] = '1'	Section Header: *KNOWLEDGE AND PERSPECTIVE OF HIV SCRIPT : Now I want to ask you some questions relating to people living with HIV and AIDS INSTRUCTION : [READ OUT the statements] 1 = Strongly disagree 2 = Disagree 3 = Agree 4 = Strongly agree*<br><br>I would stay friends with someone even if I found out he/she has HIV.	radio<br>1 Strongly disagree<br>2 Disagree<br>3 Agree<br>4 Strongly agree<br><br>Custom alignment: LV<br>Question number: Q48
127	q2_7_2<br><br>Show the field ONLY if:<br>[language] = '1' and [consent] = '1'	When you learn that you have HIV, your life is over.	radio<br>1 Strongly disagree<br>2 Disagree<br>3 Agree<br>4 Strongly agree<br><br>Custom alignment: LV<br>Question number: Q49
128	q2_7_3<br><br>Show the field ONLY if:<br>[language] = '1' and [consent] = '1'	I would be embarrassed to be seen with someone who everyone knows has HIV	radio<br>1 Strongly disagree<br>2 Disagree<br>3 Agree<br>4 Strongly agree<br><br>Custom alignment: LV<br>Question number: Q50

129	q2_7_4<br><br>Show the field ONLY if:<br>[language] = '1' and [consent] = '1'	Everyone who starts a new sexual relationship should get an HIV test before having sex.	radio<br>1 Strongly disagree<br>2 Disagree<br>3 Agree<br>4 Strongly agree<br><br>Custom alignment: LV<br>Question number: Q51
130	q2_7_5<br><br>Show the field ONLY if:<br>[language] = '1' and [consent] = '1'	To prevent getting HIV you have to use condoms every time you have sex with someone.	radio<br>1 Strongly disagree<br>2 Disagree<br>3 Agree<br>4 Strongly agree<br><br>Custom alignment: LV<br>Question number: Q52
131	q2_7_6<br><br>Show the field ONLY if:<br>[language] = '1' and [consent] = '1'	HIV is passed on most easily during the first 6 weeks after a person is infected.	radio<br>1 Strongly disagree<br>2 Disagree<br>3 Agree<br>4 Strongly agree<br><br>Custom alignment: LV<br>Question number: Q53
132	q2_7_7<br><br>Show the field ONLY if:<br>[language] = '1' and [consent] = '1'	A man who is circumcised does not need to use condoms to prevent getting HIV	radio<br>1 Strongly disagree<br>2 Disagree<br>3 Agree<br>4 Strongly agree<br><br>Custom alignment: LV<br>Question number: Q54
133	q3_1<br><br>Show the field ONLY if:<br>[language] = '1' and [consent] = '1'	Section Header: *SECTION 3 : KNOWLEDGE, ATTITUDES, AND PERCEPTIONS OF TUBERCULOSIS (TB) This section is about TB*<br><br>Do you know what Tuberculosis (TB) is?	radio<br>1 Yes<br>2 No
134	q3_1_1<br><br>Show the field ONLY if:<br>[language] = '1' and [consent] = '1'	Anybody can get TB	radio<br>1 Agree<br>2 Disagree<br>3 Don't know<br><br>Custom alignment: LV

135	q3_1_2<br><br>Show the field ONLY if:<br>[language] = '1' and [consent] = '1'	People living with HIV are more likely to get TB	radio<br>1 Agree<br>2 Disagree<br>3 Don't know<br><br>Custom alignment: LV
136	q3_1_3<br><br>Show the field ONLY if:<br>[language] = '1' and [consent] = '1'	People that are HIV negative can get TB	radio<br>1 Agree<br>2 Disagree<br>3 Don't know<br><br>Custom alignment: LV
137	q3_2<br><br>Show the field ONLY if:<br>[language] = '1' and [consent] = '1'	How can a person get TB?<br>*[MULTIPLE RESPONSES ALLOWED]*	checkbox<br>1 q3_2___1 Through handshakes<br>2 q3_2___2 Through the air when a person with TB coughs or sneezes<br>3 q3_2___3 Through sharing dishes<br>4 q3_2___4 Through eating from the same plate<br>5 q3_2___5 Through touching items in public places (doorknobs, handles in transportation etc.)<br>6 q3_2___6 Smoking<br>55 q3_2___55 Other (specify)<br>96 q3_2___96 Don't Know<br><br>Custom alignment: LV<br>Field Annotation: @NONEOFTHEABOVE = '96'
138	q3_2oth<br><br>Show the field ONLY if:<br>[q3_2(55)] = '1'	Other ways that a person can get TB	notes<br>Custom alignment: LV

139	q3_3<br><br>Show the field ONLY if:<br>[language] = '1' and [consent] = '1'	What are the signs and symptoms of TB?<br>*[MULTIPLE RESPONSES ALLOWED]*	checkbox<br>1 | q3_3___1 | Cough that lasts longer than 3 weeks<br>2 | q3_3___2 | Coughing up blood<br>3 | q3_3___3 | Loss of appetite<br>4 | q3_3___4 | Fever<br>5 | q3_3___5 | Night sweats<br>6 | q3_3___6 | Unintentional weight loss<br>7 | q3_3___7 | Other (specify)<br>8 | q3_3___8 | Don't Know<br><br>Custom alignment: LV<br>Field Annotation: @NONEOFTHEABOVE = '8'
140	q3_3oth<br><br>Show the field ONLY if:<br>[q3_3(7)] = '1'	Other specify	notes<br>Custom alignment: LV
141	q3_4<br><br>Show the field ONLY if:<br>[language] = '1' and [consent] = '1'	What is the treatment for TB?	radio<br>1 | Drugs, medicine, pills<br>2 | There is no treatment<br>3 | Traditional medicine<br>4 | Don't know<br><br>Custom alignment: LV
142	q3_5<br><br>Show the field ONLY if:<br>[q3_4] = '1'	How long does someone have to take the drugs to cure TB?	radio<br>1 | One month or less<br>2 | Two to five months<br>3 | Six months or longer<br>4 | Other (specify)<br>5 | Don't Know<br><br>Custom alignment: LV
143	q3_5oth<br><br>Show the field ONLY if:<br>[q3_5] = '4'	Q3.5 : Other specify	notes<br>Custom alignment: LV
144	q3_6<br><br>Show the field ONLY if:<br>[language] = '1' and [consent] = '1'	Are people with TB always HIV positive?	radio<br>1 | Yes<br>2 | No<br>96 | Don't know<br><br>Custom alignment: LV

145	q3_7<br>Show the field ONLY if:<br>[language] = '1' and [consent] = '1'	Is it possible to cure TB in people with HIV?	radio<br>1 Yes<br>2 No<br>96 Don't know<br>Custom alignment: LV
146	q3_8<br>Show the field ONLY if:<br>[language] = '1' and [consent] = '1'	Section Header: *Instruction: The following question is about how a person who has TB is usually regarded/treated in your community? INSTRUCTION : CHECK ONE ANSWER 1 = Strongly disagree (SD) 2 = disagree (D) 3 = Agree (A) 4 = Strongly Agree (SA)*<br>Most people reject him or her	radio<br>1 Strongly disagree<br>2 Disagree<br>3 Agree<br>4 Strongly agree<br>Custom alignment: LV
147	q3_9<br>Show the field ONLY if:<br>[language] = '1' and [consent] = '1'	Most people are friendly but they generally try to avoid him or her	radio<br>1 Strongly disagree<br>2 Disagree<br>3 Agree<br>4 Strongly agree<br>Custom alignment: LV
148	q3_10<br>Show the field ONLY if:<br>[language] = '1' and [consent] = '1'	The community mostly supports him or her	radio<br>1 Strongly disagree<br>2 Disagree<br>3 Agree<br>4 Strongly agree<br>Custom alignment: LV
149	q3_11<br>Show the field ONLY if:<br>[language] = '1' and [consent] = '1'	Has anyone in your household ever had TB?	radio<br>1 Yes<br>2 No<br>3 No response<br>Custom alignment: LV
150	q3_11a<br>Show the field ONLY if:<br>[language] = '1' and [consent] = '1'	Have you ever had a TB test?	radio<br>1 Yes<br>2 No<br>3 No response<br>Custom alignment: LV

151	q3_12<br><br>Show the field ONLY if:<br>[q3_11a] = '1'	How long ago did you have a TB Test	radio<br>1 0 to 3 Months<br>2 4 to 6 Months<br>3 7 to 11 Months<br>4 Less than a year ago<br>5 Between 1-2 years ago<br>6 Between 2-3 years ago<br>7 Three or more years ago<br><br>Custom alignment: LV
152	q3_13<br><br>Show the field ONLY if:<br>[q3_11a] = '1'	Where did you get your TB test done?	radio<br>1 At a clinic/hospital<br>2 A community worker<br>3 A traditional healer<br>4 Pharmacy<br>5 Other (specify)<br><br>Custom alignment: LV
153	q3_13oth<br><br>Show the field ONLY if:<br>[q3_13] = 5	Q3.13 : Other specify	notes<br>Custom alignment: LV
154	q3_14<br><br>Show the field ONLY if:<br>[q3_11a] = '1'	What specimen did they collect to test for TB?	radio<br>1 Blood<br>2 Sputum<br>3 Other (specify)<br><br>Custom alignment: LV
155	q_3_14oth<br><br>Show the field ONLY if:<br>[q3_14] = 3	Q3.14 : Other specify	notes
156	q4_1<br><br>Show the field ONLY if:<br>[language] = '1' and [c onsent] = '1'	Section Header: *SECTION 4 : ALCOHOL USE*<br>How often do you use drugs (dagga, benzene, tik, cocaine, nyaope, wunga, mandrax, and heroin) or something else to make you high?	radio<br>0 Never<br>1 Almost every time<br>2 Often but not every time<br>3 It has happened every week<br><br>Custom alignment: LV

157	q4_2<br>Show the field ONLY if:<br>[language] = '1' and [consent] = '1'	How often do you usually drink alcohol?	radio<br>0 I have never drank alcohol<br>1 every week<br>2 3 times a month<br>3 less than once a month<br>4 less than once in 6 months<br>Custom alignment: LV
158	q4_3<br>Show the field ONLY if:<br>[q4_2]>0	How many alcoholic drinks do you have daily?	radio<br>0 1 or 2<br>1 3 or 4<br>2 5 or 6<br>3 7, 8, or 9<br>4 10 or more<br>Custom alignment: LV
159	q4_4<br>Show the field ONLY if:<br>[q4_2]>0	How often do you have six or more drinks on one occasion?	radio<br>0 Never<br>1 Less than monthly<br>2 Monthly<br>3 Weekly<br>4 Daily or almost daily<br>Custom alignment: LV
160	q4_5<br>Show the field ONLY if:<br>[q4_2]>0	How often during the last year have you failed to do what was normally expected from you because of drinking?	radio<br>0 Never<br>1 Less than monthly<br>2 Monthly<br>3 Weekly<br>4 Daily or almost daily<br>Custom alignment: LV
161	q4_6<br>Show the field ONLY if:<br>[q4_2]>0	How often during the last year have you needed a first drink in the morning to get yourself going after a heavy drinking session?	radio<br>0 Never<br>1 Less than monthly<br>2 Monthly<br>3 Weekly<br>4 Daily or almost daily<br>Custom alignment: LV

162	q4_7<br><br>Show the field ONLY if:<br>[q4_2]>0	How often during the last year have you had a feeling of guilt or remorse after drinking?	radio<br>0 Never<br>1 Less than monthly<br>2 Monthly<br>3 Weekly<br>4 Daily or almost daily<br><br>Custom alignment: LV
163	q4_8<br><br>Show the field ONLY if:<br>[q4_2]>0	How often during the last year have you been unable to remember what happened the night before because you had been drinking?	radio<br>0 Never<br>1 Less than monthly<br>2 Monthly<br>3 Weekly<br>4 Daily or almost daily<br><br>Custom alignment: LV
164	q4_9<br><br>Show the field ONLY if:<br>[q4_2]>0	Have you or someone else been injured as a result of your drinking?	radio<br>0 No<br>1 Yes, but not in the last year<br>2 Yes, during the last year<br><br>Custom alignment: LV
165	q4_10<br><br>Show the field ONLY if:<br>[q4_2]>0	Has a relative or friend or a doctor or another health worker been concerned about your drinking or suggested you cut down?	radio<br>0 No<br>1 Yes, but not in the last year<br>2 Yes, during the last year<br><br>Custom alignment: LV
166	q5_1<br><br>Show the field ONLY if:<br>[language] = '1' and [consent] = '1'	Section Header: *SECTION 5 : SEXUAL HISTORY INSTRUCTION: This section, ask you sensitive questions on sex and other sex-related matters. Please remember that your name will not be recorded anywhere in this questionnaire and the information you give will be kept confidential.*<br><br>Have you ever had sexual intercourse? [For the purposes of this survey, "sexual intercourse" is defined as penetrative vaginal/anal sex.]	radio<br>1 Yes<br>2 No<br><br>Custom alignment: LV

167	q5_2<br><br>Show the field ONLY if:<br>[q5_1] = 2	Why have you not had sex yet?	radio<br>1 Not ready<br>2 I am too young<br>3 Not interested<br>4 Avoiding pregnancy<br>5 Avoiding STIs, including HIV<br>6 Religious grounds<br>7 Cultural grounds<br>8 Don't have a partner<br>9 I do not have access to protection (such as condoms)<br>10 No response<br>11 Other (specify)<br><br>Custom alignment: LV
168	q5_2oth<br><br>Show the field ONLY if:<br>[q5_2] = '11'	Q5.2 : Other, specify	notes<br>Custom alignment: LV
169	q5_3<br><br>Show the field ONLY if:<br>[q5_1] = 1	How old were you when you had sex for the first time?<br>[record age in years]<br>*Enter "1" if you don't remember age*	text (number)<br>Custom alignment: LV
170	q5_4<br><br>Show the field ONLY if:<br>[q5_1] = 1	Q5.4 : Who did you have sex with the first time you had it?	radio<br>1 A boyfriend or girlfriend<br>2 A friend<br>3 Husband/wife<br>4 Someone I knew for a day or less<br>5 Family member/relative<br>6 Someone I knew but he was not a friend<br>7 Someone I didn't know<br><br>Custom alignment: LV
171	q5_5<br><br>Show the field ONLY if:<br>[q5_1] = 1	How would you describe your first sexual experience?	radio<br>1 I was willing<br>2 I was persuaded<br>3 I was tricked<br>4 I was forced<br>5 I was raped<br><br>Custom alignment: LV

172	q5_6<br><br>Show the field ONLY if:<br>[q5_1] = 1	Did you use a condom the first time you had sex?	radio<br>1 Yes<br>2 No<br>3 Cannot remember<br><br>Custom alignment: LV
173	q5_7<br><br>Show the field ONLY if:<br>[q5_1] = 1	Q5.7 : In the last 12 months have you had sex?	radio<br>1 Yes<br>2 No<br>3 No response<br><br>Custom alignment: LV
174	q5_8<br><br>Show the field ONLY if:<br>[q5_1] = 1	How many sexual partners did you have in your life?<br>*PROBE : IF '00', CLARIFY THE ANSWER IN Q5.1*	text
175	q5_9<br><br>Show the field ONLY if:<br>[language] = '1' and [consent] = '1'	In the last 12 months how many sexual partners did you have?	text (number, Min: 0, Max: 99)
176	q5_10w<br><br>Show the field ONLY if:<br>[q1_4] = '2'	For women: Have you ever had sex with anybody because you expected or hoped they would give you money or something else?	radio<br>1 I got money or gifts or transport<br>2 I got food or drink or had a good time<br>3 No
177	q5_10m<br><br>Show the field ONLY if:<br>[q1_4] = '1'	For men: Have you ever had sex with anybody because the person expected or hoped you would give her or you promised her money or something else?	radio<br>1 I got money or gifts or transport<br>2 I got food or drink or had a good time<br>3 No
178	q5_11<br><br>Show the field ONLY if:<br>[q5_8] > 1	Q5.11 : Did any of these relationships mentioned above overlap with each other?	radio<br>1 Yes<br>2 No<br>3 No response
179	q5_12<br><br>Show the field ONLY if:<br>[language] = '1' and [consent] = '1'	Do you have two or more sexual partners at the moment?	radio<br>1 Yes<br>2 No<br>3 No response
180	q5_13<br><br>Show the field ONLY if:<br>[language] = '1' and [consent] = '1'	In the last 3 months how many different sexual partners did you have ?	text (number, Min: 1, Max: 99)

181	q5_14<br><br>Show the field ONLY if:<br>[language] = '1' and [consent] = '1'	In the last 3 months have you used a condom with any of your partners?	radio<br>1 Yes<br>2 No
182	q5_15<br><br>Show the field ONLY if:<br>[language] = '1' and [consent] = '1'	What is your relationship with your most recent sexual partner?	radio<br>1 Husband / Wife<br>2 Live-in partner<br>3 Girlfriend / Boyfriend Not living with me<br>4 Casual partner<br>5 Someone whom I paid/received payment from for sex<br>6 Other(specify)<br><br>Custom alignment: LV
183	q5_15oth<br><br>Show the field ONLY if:<br>[language] = '1' and [consent] = '1'	Q5.15 : Other specify	notes<br>Custom alignment: LV
184	q5_16<br><br>Show the field ONLY if:<br>[language] = '1' and [consent] = '1'	Is this most recent sexual partner a male or a female?	radio<br>1 Male<br>2 Female
185	q5_17<br><br>Show the field ONLY if:<br>[language] = '1' and [consent] = '1'	Where does this partner live?	radio<br>1 In same area<br>2 In another area<br>3 In same household<br><br>Custom alignment: LV
186	q5_18<br><br>Show the field ONLY if:<br>[language] = '1' and [consent] = '1'	What is the age of this person?<br>*Don't know = "88"*	text (number, Min: 1, Max: 88)
187	q5_19<br><br>Show the field ONLY if:<br>[language] = '1' and [consent] = '1'	Did you use a condom at last sex?	radio<br>1 Yes<br>2 No

188	q5_20<br><br>Show the field ONLY if:<br>[q5_19] = '1'	Q5.20 : If you used a condom, what were your reasons for doing so?	checkbox<br>1 | q5_20___1 | Concern about HIV infection<br>2 | q5_20___2 | People are urged to use condoms<br>3 | q5_20___3 | Want to prevent STI's<br>4 | q5_20___4 | Want to prevent pregnancy<br>5 | q5_20___5 | I or partner on ARV<br>6 | q5_20___6 | Partner insisted on condom use<br>7 | q5_20___7 | Other: please specify<br>8 | q5_20___8 | Don't know<br><br>Custom alignment: LV<br>Field Annotation: @NONEOFTHEABOVE = '8'
189	q5_20oth<br><br>Show the field ONLY if:<br>[q5_20(7)] = '1'	Q5.20 : Other specify	notes<br>Custom alignment: LV
190	q5_21<br><br>Show the field ONLY if:<br>[q5_19] = '2'	Q5.21 : If you DID NOT use a condom, what were your reasons for doing so?	checkbox<br>1 | q5_21___1 | Did not have a condom<br>2 | q5_21___2 | Partner objected<br>3 | q5_21___3 | Used other contraceptive<br>4 | q5_21___4 | Don't like them<br>5 | q5_21___5 | Didn't think it was necessary<br>6 | q5_21___6 | I am married<br>7 | q5_21___7 | I am faithful/trust them<br>8 | q5_21___8 | I was drunk/high<br>9 | q5_21___9 | I did not know how to use the condom<br>10 | q5_21___10 | Other(specify)<br>11 | q5_21___11 | Don'nt know<br><br>Custom alignment: LV<br>Field Annotation: @NONEOFTHEABOVE = '11'
191	q5_21oth<br><br>Show the field ONLY if:<br>[q5_21(10)] = '1'	Q5.21: Other specify	notes<br>Custom alignment: LV

192	q5_22<br><br>Show the field ONLY if:<br>[language] = '1' and [consent] = '1'	Is it easy to get a male condom if you need one?	radio<br>1 Yes<br>2 No<br>3 No response
193	q5_23<br><br>Show the field ONLY if:<br>[language] = '1' and [consent] = '1'	Is it easy to get a female condom if you need one?	radio<br>1 Yes<br>2 No<br>3 No response
194	q5_24<br><br>Show the field ONLY if:<br>[language] = '1' and [consent] = '1'	Where do you normally obtain your condoms?	checkbox<br>1 q5_24___1 clinic or hospital<br>2 q5_24___2 Pharmacy/chemist<br>3 q5_24___3 Shop/supermarket/cafe shop<br>4 q5_24___4 garage/ petrol station<br>5 q5_24___5 shebeen/tavern/pub<br>6 q5_24___6 from my friends<br>7 q5_24___7 From My partner<br>8 q5_24___8 From my parent/s<br>9 q5_24___9 Not Applicable<br>10 q5_24___10 Other(specify)<br>11 q5_24___11 Community health work<br><br>Custom alignment: LV<br>Field Annotation: @NONEOFTHEABOVE = '9'
195	q5_24oth<br><br>Show the field ONLY if:<br>[q5_24(10)] = '1'	Other specify	notes<br>Custom alignment: LV
196	q6_1<br><br>Show the field ONLY if:<br>[language] = '1' and [consent] = '1'	Section Header: *SECTION 6 : HIV COUNSELLING AND TESTING Instruction: The following questions are about testing for HIV. Please remember that your name will not be recorded anywhere in this questionnaire and the information you give will be kept confidential.*<br>Have you ever had an HIV test?	radio<br>1 Yes<br>2 No
197	q6_2<br><br>Show the field ONLY if:<br>[language] = '1' and [consent] = '1'	Do you know of a place nearby where you can get an HIV test?	radio<br>1 Yes<br>2 No

198	q6_3<br>Show the field ONLY if:<br>[q6_1] = '1'	How long ago did you have an HIV test/retest?	radio<br>1 0 to 3 months<br>2 4 to 6 months<br>3 7 to 11 months<br>4 Less than a year ago<br>5 Between 1-2 years ago<br>6 Between 2-3 years ago<br>7 Three or more years ago<br>Custom alignment: LV
199	q6_4<br>Show the field ONLY if:<br>[q6_1] = '1'	Where did you get your most recent HIV test?	radio<br>1 Public hospital<br>2 Private hospital<br>3 Public clinic or doctor<br>4 Private clinic or doctor<br>5 [Local NGO/ community based organization]<br>6 Workplace<br>7 Community testing event<br>8 Other<br>Custom alignment: LV
200	q6_5<br>Show the field ONLY if:<br>[language] = '1' and [consent] = '1'	Where would you prefer to go for an HIV test?	radio<br>1 Public hospital<br>2 Private hospital<br>3 Public clinic or doctor<br>4 Private clinic or doctor<br>5 [Name of Local NGO/ community based organization]<br>6 Workplace<br>7 Community testing event<br>8 Other<br>Custom alignment: LV
201	q6_5oth<br>Show the field ONLY if:<br>[q6_5] = '8'	Q6.5 : Other, specify	notes<br>Custom alignment: LV
202	q6_6<br>Show the field ONLY if:<br>[q6_1] = '1'	Section Header: *Instruction: Please note that you should not disclose the actual result. I am only interested in knowing whether you have been told/informed of the result of the test.*<br>During your most recent HIV test, did you have counselling after the HIV test?	radio<br>1 Yes<br>2 No

203	q6_7<br><br>Show the field ONLY if:<br>[q6_1] = '1'	During your most recent HIV test, were you given information individually or in a group?	radio<br>1 individually<br>2 in a group
204	q6_8<br><br>Show the field ONLY if:<br>[language] = '1' and [consent] = '1'	Have you been told/informed of the result of your most recent test?	radio<br>1 Yes<br>2 No
205	q6_9<br><br>Show the field ONLY if:<br>[language] = '1' and [consent] = '1'	During your most recent HIV test, did you have counselling after the HIV test?	radio<br>1 Yes<br>2 No
206	q6_10<br><br>Show the field ONLY if:<br>[language] = '1' and [consent] = '1'	What were your reasons for going for your last HIV test?<br>*MULTIPLE RESPONSES POSSIBLE*	checkbox<br>1 q6_10___1 I wanted to know my HIV status<br>2 q6_10___2 My partner asked me to go for testing<br>3 q6_10___3 I wanted to start a new sexual relationship<br>4 q6_10___4 I wanted to get married<br>5 q6_10___5 I applied for an insurance policy<br>6 q6_10___6 I applied for a loan<br>7 q6_10___7 My employer requested it<br>8 q6_10___8 I was feeling sick<br>9 q6_10___9 I was instructed by a health worker (nurse/doctor)<br>10 q6_10___10 I was pregnant<br>11 q6_10___11 Workplace campaign<br>12 q6_10___12 Other<br><br>Custom alignment: LV
207	q6_10oth<br><br>Show the field ONLY if:<br>[q6_10(12)] = '1'	Other specify	notes<br>Custom alignment: LV
208	q6_11<br><br>Show the field ONLY if:<br>[language] = '1' and [consent] = '1'	You indicated that you were previously tested for HIV. Are you willing to tell me the last HIV test result you received?	radio<br>1 Yes<br>2 No<br>3 Never received

209	q6_12a<br><br>Show the field ONLY if:<br>[q6_11] = '1'	What was the result of that HIV test?	radio<br>1 Positive<br>2 Negative<br>3 Indeterminate<br><br>Custom alignment: LV
210	q6_12bm<br><br>Show the field ONLY if:<br>[q6_12a] = '1'	What was the month of your first HIV positive test? (MONTH)	radio<br>1 JAN<br>2 FEB<br>3 MAR<br>4 APR<br>5 MAY<br>6 JUN<br>7 JUL<br>8 AUG<br>9 SEP<br>10 OCT<br>11 NOV<br>12 DEC<br><br>Custom alignment: LV
211	q6_12by<br><br>Show the field ONLY if:<br>[q6_12a] = '1'	What was the year of your first HIV positive test? (YEAR)	text (number, Max: 8888)<br>Field Annotation: @CHARLIMIT = '4'
212	q6_13<br><br>Show the field ONLY if:<br>[q6_12a] = '2'	Are you currently taking ARVs, that is, antiretroviral medications to treat HIV?	radio<br>1 Yes<br>2 No
213	q6_14<br><br>Show the field ONLY if:<br>[q6_11] = '1' and [q6_12a] = '2' and [q6_13] = '1'	How long have you been taking daily ARVs?<br>*Record duration in Months (1yr = 1*12)*	text (number)
214	q6_15<br><br>Show the field ONLY if:<br>[q6_11] = '1' and [q6_12a] = '2' and [q6_13] = '1'	Did you tell your main partner about your HIV status?	radio<br>1 Yes<br>2 No<br>3 no reponse
215	q6_16<br><br>Show the field ONLY if:<br>[language] = '1' and [consent] = '1'	Have you told other sexual partners about your test result during the last 12 months?	radio<br>1 Yes<br>2 No<br>3 No Partner

<table>
<tr><td>216</td><td>q6_17

Show the field ONLY if:
[language] = '1' and [consent] = '1'</td><td>Have you ever taken an HIV test with any of your sex partners where you both received the test results together?</td><td>radio
1 Yes
2 No</td></tr>
<tr><td>217</td><td>q6_18

Show the field ONLY if:
[q6_17]= "2"</td><td>What are your reasons for not going for an HIV test?</td><td>checkbox
1 q6_18___1 Do not know where to get tested
2 q6_18___2 Do not think that I have HIV
3 q6_18___3 Not at risk for HIV
4 q6_18___4 Trust partner
5 q6_18___5 Afraid to find out that he/she might be HIV positive
6 q6_18___6 Not ready to have an HIV test
7 q6_18___7 Concerned about confidentiality
8 q6_18___8 Concerned about stigma, discrimination, or rejection
9 q6_18___9 Concerned about losing my job
10 q6_18___10 Concerned about the standard of service
11 q6_18___11 Haven't got around to do it
12 q6_18___12 I am concerned about the costs
13 q6_18___13 Other

Custom alignment: LV</td></tr>
<tr><td>218</td><td>q6_18oth

Show the field ONLY if:
[q6_18(13)] = '1'</td><td>Q6.18 : Other specify</td><td>notes
Custom alignment: LV</td></tr>
</table>

219	q6_19<br><br>Show the field ONLY if:<br>[language] = '1' and [consent] = '1'	Out of 10, how many of your friends do you think have been tested for HIV?	radio<br>1 | 1 out of 10<br>2 | 2 out of 10<br>3 | 3 out of 10<br>4 | 4 out of 10<br>5 | 5 out of 10<br>6 | 6 out of 10<br>7 | 7 out of 10<br>8 | 8 out of 10<br>9 | 9 out of 10<br>10 | 10 out of 10<br><br>Custom alignment: LV
220	q6_20a<br><br>Show the field ONLY if:<br>[language] = '1' and [consent] = '1'	Section Header: *HIV TESTING IN YOUR COMMUNITY SCRIPT: How much do you agree with the following statements about HIV testing in your community? There are no right or wrong answers, only people's opinions. We would like to know which opinions you agree or disagree with. INSTRUCTION : Interviewer read out loud 1 = Strongly disagree (SD) 2 = Disagree (D) 3 = Agree (A) 4 = Strongly Agree (SA)*<br>People in my community who test positive feel free to tell their friends and family that they have HIV	radio<br>1 | Strongly disagree<br>2 | Disagree<br>3 | Agree<br>4 | Strongly Agree<br><br>Custom alignment: LV<br>Question number: Q128
221	q6_20b<br><br>Show the field ONLY if:<br>[language] = '1' and [consent] = '1'	People in my community do not test for HIV because they are scared it would change their lifestyle (i.e. use condoms, reduce number of partners, stop drinking alcohol and smoking)	radio<br>1 | Strongly disagree<br>2 | Disagree<br>3 | Agree<br>4 | Strongly Agree<br><br>Custom alignment: LV<br>Question number: Q129
222	q6_20c<br><br>Show the field ONLY if:<br>[language] = '1' and [consent] = '1'	People in my community do not test for HIV because they are scared that they are already HIV positive	radio<br>1 | Strongly disagree<br>2 | Disagree<br>3 | Agree<br>4 | Strongly Agree<br><br>Custom alignment: LV<br>Question number: Q130
223	q6_20d<br><br>Show the field ONLY if:<br>[language] = '1' and [consent] = '1'	People in my community are scared to test for HIV because they think that if they test HIV positive, they can never have a relationship again	radio<br>1 | Strongly disagree<br>2 | Disagree<br>3 | Agree<br>4 | Strongly Agree<br><br>Custom alignment: LV<br>Question number: Q131

224	q6_20e<br><br>Show the field ONLY if:<br>[language] = '1' and [consent] = '1'	People in my community who test for HIV are regarded as strong and responsible	radio<br>1 Strongly disagree<br>2 Disagree<br>3 Agree<br>4 Strongly Agree<br><br>Custom alignment: LV<br>Question number: Q132
225	q6_20f<br><br>Show the field ONLY if:<br>[language] = '1' and [consent] = '1'	People in my community are scared to test for HIV because if they test positive their family and friends will reject them	radio<br>1 Strongly disagree<br>2 Disagree<br>3 Agree<br>4 Strongly Agree<br><br>Custom alignment: LV<br>Question number: Q133
226	q7_1<br><br>Show the field ONLY if:<br>[language] = '1' and [consent] = '1'	Section Header: *SECTION 7 : HIV RISK PERCEPTION, ATTITUDES AND SOCIAL NORMS FOR MULTIPLE PARTNERS AND CONDOMS*<br><br>Choose the statement that best describes yourself in terms of HIV infection	radio<br>1 I am definitely going to get infected with HIV<br>2 I am probably going to get infected with HIV<br>3 I probably won't get infected with HIV<br>4 I definitely will not get infected with HIV<br><br>Custom alignment: LV

227	q7_2<br>Show the field ONLY if:<br>[q7_1] = '4'	What are your reasons for believing that you probably or definitely will NOT get infected with HIV?<br>*MULTIPLE RESPONSES POSSIBLE*	checkbox<br>1 q7_2___1 Never had sex before<br>2 q7_2___2 Abstain/withholding from sex<br>3 q7_2___3 Faithful to his/her partner<br>4 q7_2___4 Trust his/her partner<br>5 q7_2___5 Use condoms<br>6 q7_2___6 Know his/her HIV status<br>7 q7_2___7 Know the HIV status of his/her partner<br>8 q7_2___8 Do not have sex with sex workers/prostitutes<br>9 q7_2___9 Protected by ancestors<br>10 q7_2___10 Protected by God<br>11 q7_2___11 I am not at risk for HIV<br>12 q7_2___12 Other<br>Custom alignment: LV
228	q7_2oth<br>Show the field ONLY if:<br>[q7_2(12)] = '1'	Q7.2 : Other specify	notes<br>Custom alignment: LV
229	q7_3<br>Show the field ONLY if:<br>[q7_1] = '1' or [q7_1] = '2'	What are your reasons for believing that you probably or definitely will get infected with HIV?<br>*MULTIPLE RESPONSES POSSIBLE*	checkbox<br>1 q7_3___1 Sexually active<br>2 q7_3___2 Had many sexual partners<br>3 q7_3___3 Don't use condoms<br>4 q7_3___4 Don't always use condoms<br>5 q7_3___5 Don't trust his/her partner<br>6 q7_3___6 I am sick<br>7 q7_3___7 My partner is sick<br>8 q7_3___8 My partner died of AIDS<br>9 q7_3___9 Had an accident/cuts<br>10 q7_3___10 my partner is HIV positive<br>11 q7_3___11 Other(specify)<br>Custom alignment: LV

230	q7_3oth<br><br>Show the field ONLY if:<br>[q7_3(11)] = '1'	Q7.3 : Other specify	notes<br>Custom alignment: LV
231	q7_4<br><br>Show the field ONLY if:<br>[language] = '1' and [consent] = '1'	Would you be willing to test yourself for HIV if you were to be given an HIV self-test kit?	radio<br>1 Yes<br>2 No<br>3 Don't know<br><br>Custom alignment: LV
232	q7_5a<br><br>Show the field ONLY if:<br>[q1_4] = '1'	Section Header: *CONDOMS AND RELATIONSHIPS*<br>(Men) Men are afraid that his wife/girlfriend will turn him down if he suggests using a condom.	radio<br>1 Strongly disagree<br>2 Disagree<br>3 Agree<br>4 Strongly Agree<br><br>Custom alignment: LV<br>Question number: Q138
233	q7_5b<br><br>Show the field ONLY if:<br>[q1_4] = '2'	(Women) Women are afraid that her husband/boyfriend will turn her down if she suggests using a condom.	radio<br>1 Strongly disagree<br>2 Disagree<br>3 Agree<br>4 Strongly Agree<br><br>Custom alignment: LV<br>Question number: Q139
234	q7_5c<br><br>Show the field ONLY if:<br>[language] = '1' and [consent] = '1'	If someone ever has trouble putting on a condom, they will be embarrassed to try to use a condom again.	radio<br>1 Strongly disagree<br>2 Disagree<br>3 Agree<br>4 Strongly Agree<br><br>Custom alignment: LV<br>Question number: Q140
235	q7_5d<br><br>Show the field ONLY if:<br>[language] = '1' and [consent] = '1'	Women who carry condoms are looking out for their own health.	radio<br>1 Strongly disagree<br>2 Disagree<br>3 Agree<br>4 Strongly Agree<br><br>Custom alignment: LV<br>Question number: Q141

236	q7_5e<br><br>Show the field ONLY if:<br>[language] = '1' and [consent] = '1'	Using a condom will make your partner think you dont trust him/her.	radio<br>1 Strongly disagree<br>2 Disagree<br>3 Agree<br>4 Strongly Agree<br><br>Custom alignment: LV<br>Question number: Q142
237	q7_5f<br><br>Show the field ONLY if:<br>[language] = '1' and [consent] = '1'	When you use a condom, you can't get enough pleasure.	radio<br>1 Strongly disagree<br>2 Disagree<br>3 Agree<br>4 Strongly Agree<br><br>Custom alignment: LV<br>Question number: Q143
238	q7_5g<br><br>Show the field ONLY if:<br>[language] = '1' and [consent] = '1'	If you have good communication with your partner, you can be sexually satisfied with one person.	radio<br>1 Strongly disagree<br>2 Disagree<br>3 Agree<br>4 Strongly Agree<br><br>Custom alignment: LV<br>Question number: Q144
239	q7_5h<br><br>Show the field ONLY if:<br>[language] = '1' and [consent] = '1'	I need someone else to fill the gap in case I ever break up with my main partner.	radio<br>1 Strongly disagree<br>2 Disagree<br>3 Agree<br>4 Strongly Agree<br><br>Custom alignment: LV<br>Question number: Q145
240	q7_5i<br><br>Show the field ONLY if:<br>[language] = '1' and [consent] = '1'	Now and then, I go to someone else besides my main partner because the sex is so good.	radio<br>1 Strongly disagree<br>2 Disagree<br>3 Agree<br>4 Strongly Agree<br><br>Custom alignment: LV<br>Question number: Q146

241	q7_5j<br><br>Show the field ONLY if:<br>[language] = '1' and [consent] = '1'	It's ok to have sex with others as long as your main partner does not find out.	radio<br>1 Strongly disagree<br>2 Disagree<br>3 Agree<br>4 Strongly Agree<br><br>Custom alignment: LV<br>Question number: Q147
242	q7_5k<br><br>Show the field ONLY if:<br>[language] = '1' and [consent] = '1'	Most of my friends brag about how many people they are having sex with.	radio<br>1 Strongly disagree<br>2 Disagree<br>3 Agree<br>4 Strongly Agree<br><br>Custom alignment: LV<br>Question number: Q148
243	q7_5l<br><br>Show the field ONLY if:<br>[language] = '1' and [consent] = '1'	Condoms are a lot of fun	radio<br>1 Strongly disagree<br>2 Disagree<br>3 Agree<br>4 Strongly Agree<br><br>Custom alignment: LV<br>Question number: Q149
244	q7_6<br><br>Show the field ONLY if:<br>[language] = '1' and [consent] = '1'	How many men in your community do you think have sex with more than one woman?	text (number, Min: 0, Max: 99)
245	q7_7<br><br>Show the field ONLY if:<br>[language] = '1' and [consent] = '1'	Do you think that this practice of men having sex with more than one woman is acceptable or not?	radio<br>1 Acceptable<br>2 Not acceptable<br>3 Don't know
246	q7_8<br><br>Show the field ONLY if:<br>[language] = '1' and [consent] = '1'	About how many women in your community do you think have sex with more than one man?	text (number, Min: 0, Max: 99)
247	q7_9<br><br>Show the field ONLY if:<br>[language] = '1' and [consent] = '1'	Personally, do you think that this practice of women having sex with more than one man is acceptable or not?	radio<br>1 Acceptable<br>2 Not acceptable<br>3 Don't know

248	q7_10a<br>Show the field ONLY if:<br>[language] = '1' and [consent] = '1'	Section Header: *STATEMENTS ABOUT YOURSELF Instruction: How much do you agree with the following statements about yourself? 1 = Strongly disagree (SD) 2 = Disagree (D) 3 = Agree (A) 4 = Strongly agree (SA)*<br>I can use a condom even when I have too much to drink.	radio<br>1 Strongly disagree<br>2 Disagree<br>3 Agree<br>4 Strongly Agree<br>Custom alignment: LV<br>Question number: Q154
249	q7_10b<br>Show the field ONLY if:<br>[language] = '1' and [consent] = '1'	I can refuse to have sex if someone I like refuses to use a condom.	radio<br>1 Strongly disagree<br>2 Disagree<br>3 Agree<br>4 Strongly Agree<br>Custom alignment: LV<br>Question number: Q155
250	q7_10c<br>Show the field ONLY if:<br>[language] = '1' and [consent] = '1'	I can buy condoms without feeling embarrassed.	radio<br>1 Strongly disagree<br>2 Disagree<br>3 Agree<br>4 Strongly Agree<br>Custom alignment: LV<br>Question number: Q156
251	q7_10dm<br>Show the field ONLY if:<br>[q1_4] = '1'	[MEN) I am confident that I can put a condom on correctly.	radio<br>1 Strongly disagree<br>2 Disagree<br>3 Agree<br>4 Strongly Agree<br>Custom alignment: LV<br>Question number: Q157
252	q7_10dw<br>Show the field ONLY if:<br>[q1_4] = '2'	[WOMEN] I am confident that I can correctly put a condom on a man when having sex with him	radio<br>1 Strongly disagree<br>2 Disagree<br>3 Agree<br>4 Strongly Agree<br>Custom alignment: LV<br>Question number: Q158
253	q8_1m<br>Show the field ONLY if:<br>[q1_4] = '1'	Section Header: *SECTION 8 : MALE CIRCUMCISION*<br>(MEN): Are you circumcised?	radio<br>1 Yes<br>2 No<br>3 No response

254	q8_1w<br><br>Show the field ONLY if:<br>[q1_4] = '2'	(WOMEN): Is your partner circumcised?	radio<br>1 Yes<br>2 No<br>3 No response
255	q8_2m<br><br>Show the field ONLY if:<br>[q1_4] = '1' and [q8_1m] = '1'	Why were you circumcised?	checkbox<br>1 q8_2m___1 Heard in the media<br>2 q8_2m___2 My parents decided<br>3 q8_2m___3 Cultural tradition/religion<br>4 q8_2m___4 To prevent HIV<br>5 q8_2m___5 To prevent STIs<br>6 q8_2m___6 Other (specify)<br>7 q8_2m___7 Refused to answer<br><br>Custom alignment: LV<br>Field Annotation: @NONEOFTHEABOVE = '7'
256	q8_2w<br><br>Show the field ONLY if:<br>[q1_4] = '2' and [q8_1w] = '1'	Why was your partner circumcised?	checkbox<br>1 q8_2w___1 Heard in the media<br>2 q8_2w___2 His parents decided<br>3 q8_2w___3 Cultural tradition/religion<br>4 q8_2w___4 To prevent HIV<br>5 q8_2w___5 To prevent STIs<br>6 q8_2w___6 Other (specify)<br>7 q8_2w___7 Refused to answer<br><br>Custom alignment: LV<br>Field Annotation: @NONEOFTHEABOVE = 7

<table>
<tr>
<td>257</td>
<td>q8_3m

Show the field ONLY if:
[q1_4] = '1' and [q8_1m] <> '1'</td>
<td>Why have you not been circumcised?</td>
<td>checkbox
<table>
<tr><td>1</td><td>q8_3m___1</td><td>One can become infertile</td></tr>
<tr><td>2</td><td>q8_3m___2</td><td>Reduces sexual pleasure</td></tr>
<tr><td>3</td><td>q8_3m___3</td><td>Unsure about safety of procedure</td></tr>
<tr><td>4</td><td>q8_3m___4</td><td>None of my friends are circumcised</td></tr>
<tr><td>5</td><td>q8_3m___5</td><td>My friends will make fun of me</td></tr>
<tr><td>6</td><td>q8_3m___6</td><td>My partner opposes</td></tr>
<tr><td>7</td><td>q8_3m___7</td><td>Not part of my culture/tradition</td></tr>
<tr><td>8</td><td>q8_3m___8</td><td>I am afraid of the pain</td></tr>
<tr><td>9</td><td>q8_3m___9</td><td>I do not want to abstain from sex for long (six weeks)</td></tr>
<tr><td>10</td><td>q8_3m___10</td><td>Other (Specify)</td></tr>
<tr><td>11</td><td>q8_3m___11</td><td>Refused to answer</td></tr>
</table>

Custom alignment: LV
Field Annotation: @NONEOFTHEABOVE = 11</td>
</tr>
</table>

<table>
<tr>
<td>258</td>
<td>q8_3w

Show the field ONLY if:
[q1_4] = '2' and [q8_1w] <> '1'</td>
<td>Why is your partner not circumcised?</td>
<td>checkbox
<table>
<tr><td>1</td><td>q8_3w___1</td><td>One can become infertile</td></tr>
<tr><td>2</td><td>q8_3w___2</td><td>Reduces sexual pleasure</td></tr>
<tr><td>3</td><td>q8_3w___3</td><td>Unsure about safety of procedure</td></tr>
<tr><td>4</td><td>q8_3w___4</td><td>None of my friends are circumcised</td></tr>
<tr><td>5</td><td>q8_3w___5</td><td>His friends will make fun of him</td></tr>
<tr><td>6</td><td>q8_3w___6</td><td>I opposed</td></tr>
<tr><td>7</td><td>q8_3w___7</td><td>Not part of his culture/tradition</td></tr>
<tr><td>8</td><td>q8_3w___8</td><td>Afraid of the pain</td></tr>
<tr><td>9</td><td>q8_3w___9</td><td>Does not want to abstain from sex for long (six weeks)</td></tr>
<tr><td>10</td><td>q8_3w___10</td><td>Other (Specify)</td></tr>
<tr><td>11</td><td>q8_3w___11</td><td>Refused to answer</td></tr>
</table>
Custom alignment: LV
Field Annotation: @NONEOFTHEABOVE = 11</td>
</tr>
<tr>
<td>259</td>
<td>q8_4m

Show the field ONLY if:
[q1_4] = '1' and [q8_1m] <> '1'</td>
<td>In the next 12 months do you plan to get circumcised?</td>
<td>radio
<table>
<tr><td>1</td><td>Definitely will</td></tr>
<tr><td>2</td><td>Probably will</td></tr>
<tr><td>3</td><td>Probably will not</td></tr>
<tr><td>4</td><td>Definitely will not</td></tr>
<tr><td>5</td><td>Unsure</td></tr>
</table>
Custom alignment: LV</td>
</tr>
<tr>
<td>260</td>
<td>q8_4w

Show the field ONLY if:
[q1_4] = '2' and [q8_1w] <> '1'</td>
<td>In the next 12 months does your partner planning to get circumcised?</td>
<td>radio
<table>
<tr><td>1</td><td>Definitely will</td></tr>
<tr><td>2</td><td>Probably will</td></tr>
<tr><td>3</td><td>Probably will not</td></tr>
<tr><td>4</td><td>Definitely will not</td></tr>
<tr><td>5</td><td>Unsure</td></tr>
</table>
Custom alignment: LV</td>
</tr>
<tr>
<td>261</td>
<td>q9_1

Show the field ONLY if:
[language] = '1' and [consent] = '1'</td>
<td>Section Header: SECTION 9 : PRE-EXPOSURE PROPHYLAXIS
Instruction: The following questions are about the use of medication to help prevent HIV infections. Remember there are no right or wrong answers and anything you say will be kept confidential. If you do not want to answer a question, you may skip to the next question.
Have you ever heard about Pre-exposure prophylaxis, which is also called PrEP?</td>
<td>radio
<table>
<tr><td>1</td><td>Yes</td></tr>
<tr><td>2</td><td>No</td></tr>
</table></td>
</tr>
</table>

262	q9_2<br><br>Show the field ONLY if:<br>[language] = '1' and [consent] = '1'	Have you ever heard about HIV medication that is taken to help prevent becoming infected with HIV?	radio<br>1 Yes<br>2 No
263	q9_3<br><br>Show the field ONLY if:<br>[language] = '1' and [consent] = '1'	Section Header: *INFORMATION: PrEP stands for Pre-Exposure Prophylaxis. It is a pill that is taken every day by people who are HIV negative to help prevent them from becoming HIV positive.*<br><br>Would you be interested in using PrEP?	radio<br>1 Yes<br>2 No<br>3 Not sure
264	q9_4<br><br>Show the field ONLY if:<br>[q9_3] = '1' or [q9_3] = '3'	How would you prefer to get access to PrEP? [Choose the two most important]	checkbox<br>1 q9_4___1 Private doctor<br>2 q9_4___2 Hospital<br>3 q9_4___3 Clinic<br>4 q9_4___4 Pharmacy/Chemist<br>5 q9_4___5 School<br>6 q9_4___6 Community health workers<br>7 q9_4___7 Non-governmental organization (NGO)<br>8 q9_4___8 Traditional healer<br>9 q9_4___9 Mobile clinic<br>10 q9_4___10 Family member<br>11 q9_4___11 Other(specify)<br><br>Custom alignment: LV
265	q9_5<br><br>Show the field ONLY if:<br>[q9_3] = '2'	I would not use PrEP because: [Choose the two most important]	checkbox<br>1 q9_5___1 Possible side effects<br>2 q9_5___2 I don't know enough about it<br>3 q9_5___3 I would not want my family or friends to know that I'm using it<br>4 q9_5___4 If I use it I would be more sexually active<br>5 q9_5___5 I don't want to drink a pill every day<br>6 q9_5___6 If I use it I will have riskier sex, like not using a condom<br>7 q9_5___7 Other(specify)<br><br>Custom alignment: LV

266	q9_6<br><br>Show the field ONLY if:<br>[q9_3] = '1' or [q9_3] = '3'	What are the three most important things you would like to know about PrEP to help you decide if you would want to use it?<br>[Choose the three most important]	checkbox<br>1 | q9_6___1 | Side effects<br>2 | q9_6___2 | Place where I can get the pill<br>3 | q9_6___3 | Person who gives the medicine<br>4 | q9_6___4 | Duration of taking the pill<br>5 | q9_6___5 | How well this pill works<br>6 | q9_6___6 | How often I have to take it (once a day vs. before sex acts)<br>7 | q9_6___7 | Cost of the pill<br>8 | q9_6___8 | How it is taken<br>9 | q9_6___9 | Other (specify)<br><br>Custom alignment: LV
267	q9_6oth<br><br>Show the field ONLY if:<br>[q9_6(9)] = '1'	Q9.6 : Other, specify	notes<br>Custom alignment: LV
268	q9_7<br><br>Show the field ONLY if:<br>[language] = '1' and [consent] = '1'	[We would like you to advise us on how best to give information about PrEP to you and your friends.]<br>What would be the best way of telling young people in your community about PrEP?<br>[Choose the three most important]	checkbox<br>1 | q9_7___1 | Newspaper<br>2 | q9_7___2 | Billboards advertisements<br>3 | q9_7___3 | School visits<br>4 | q9_7___4 | TV advertisements<br>5 | q9_7___5 | Brochures handed out at health facilities<br>6 | q9_7___6 | Social media like Facebook and WhatsApp<br>7 | q9_7___7 | Other(specify)<br><br>Custom alignment: LV
269	q9_7oth<br><br>Show the field ONLY if:<br>[q9_7(7)] = '1'	Q9.7 : Other, specify	notes<br>Custom alignment: LV

270	q10_1a<br><br>Show the field ONLY if:<br>[language] = '1' and [consent] = '1'	Section Header: *SECTION 10 : MEDIA, COMMUNICATION AND NORMS Instruction: The following questions are about sources of information and what you think of them*<br><br>How often do you listen to the radio ?	radio<br>1 Never<br>2 Once in a while/ rarely<br>3 Once a week<br>4 2-6 days a week<br>5 Every day of the week<br><br>Custom alignment: LV
271	q10_2b<br><br>Show the field ONLY if:<br>[language] = '1' and [consent] = '1'	How often do you watch television?	radio<br>1 Never<br>2 Once in a while/ rarely<br>3 Once a week<br>4 2-6 days a week<br>5 Every day of the week<br><br>Custom alignment: LV
272	q10_3c<br><br>Show the field ONLY if:<br>[language] = '1' and [consent] = '1'	How often do you read a print magazine ?	radio<br>1 Never<br>2 Once in a while/ rarely<br>3 Once a week<br>4 2-6 days a week<br>5 Every day of the week<br><br>Custom alignment: LV
273	q10_4d<br><br>Show the field ONLY if:<br>[language] = '1' and [consent] = '1'	How often do you read a print newspaper ?	radio<br>1 Never<br>2 Once in a while/ rarely<br>3 Once a week<br>4 2-6 days a week<br>5 Every day of the week<br><br>Custom alignment: LV
274	q10_5e<br><br>Show the field ONLY if:<br>[language] = '1' and [consent] = '1'	How often do you use the internet to go onto news sites ?	radio<br>1 Never<br>2 Once in a while/ rarely<br>3 Once a week<br>4 2-6 days a week<br>5 Every day of the week<br><br>Custom alignment: LV

275	q10_6f<br>Show the field ONLY if:<br>[language] = '1' and [consent] = '1'	How often do you use cell phone or computer or Tablet to go onto Facebook ?	radio<br>1 Never<br>2 Once in a while/ rarely<br>3 Once a week<br>4 2-6 days a week<br>5 Every day of the week<br>Custom alignment: LV
276	q10_7g<br>Show the field ONLY if:<br>[language] = '1' and [consent] = '1'	How often do you use cell phone or computer or Tablet to go onto Twitter?	radio<br>1 Never<br>2 Once in a while/ rarely<br>3 Once a week<br>4 2-6 days a week<br>5 Every day of the week<br>Custom alignment: LV
277	q10_8h<br>Show the field ONLY if:<br>[language] = '1' and [consent] = '1'	How often do you use the cell phone or computer or Tablet to go onto HIV or other health related websites ?	radio<br>1 Never<br>2 Once in a while/ rarely<br>3 Once a week<br>4 2-6 days a week<br>5 Every day of the week<br>Custom alignment: LV
278	q10_9i<br>Show the field ONLY if:<br>[language] = '1' and [consent] = '1'	How often do you use cell phone or the internet to go onto Instagram ?	radio<br>1 Never<br>2 Once in a while/ rarely<br>3 Once a week<br>4 2-6 days a week<br>5 Every day of the week<br>Custom alignment: LV
279	q10_10j<br>Show the field ONLY if:<br>[language] = '1' and [consent] = '1'	How often do you use a cellphone to go onto WhatsApp ?	radio<br>1 Never<br>2 Once in a while/ rarely<br>3 Once a week<br>4 2-6 days a week<br>5 Every day of the week<br>Custom alignment: LV

280	q10_2_1<br><br>Show the field ONLY if:<br>[language] = '1' and [consent] = '1'	Section Header: *Which radio stations do you listen to the most? [Allow list up to 3 stations]*<br><br>Which radio stations do you listen to the most? (Radio Station 1)	text
281	q10_2_2<br><br>Show the field ONLY if:<br>[language] = '1' and [consent] = '1'	Which radio stations do you listen to the most? (Radio Station 2)	text
282	q10_2_3<br><br>Show the field ONLY if:<br>[language] = '1' and [consent] = '1'	Which radio stations do you listen to the most? (Radio Station 3)	text
283	q10_3_1<br><br>Show the field ONLY if:<br>[language] = '1' and [consent] = '1'	Section Header: *Which Which TV stations do you watch the most? [Allow list up to 3 stations]*<br><br>(TV Station 1)	text
284	q10_3_2<br><br>Show the field ONLY if:<br>[language] = '1' and [consent] = '1'	(TV Station 2)	text
285	q10_3_3<br><br>Show the field ONLY if:<br>[language] = '1' and [consent] = '1'	(TV Station 3)	text
286	q10_4_1<br><br>Show the field ONLY if:<br>[language] = '1' and [consent] = '1'	Section Header: *Which websites do you visit most often? ([Allow list up to 3 websites)*<br><br>(Website 1)	text
287	q10_4_2<br><br>Show the field ONLY if:<br>[language] = '1' and [consent] = '1'	(Website 2)	text
288	q10_4_3<br><br>Show the field ONLY if:<br>[language] = '1' and [consent] = '1'	(Website 3)	text
289	q10_5_1<br><br>Show the field ONLY if:<br>[language] = '1' and [consent] = '1'	Section Header: *Which facebook pages or groups do you follow most often? ([Allow list up to 3 websites)*<br><br>(Facebook Page/Group 1)	text

290	q10_5_2<br><br>Show the field ONLY if:<br>[language] = '1' and [consent] = '1'	(Facebook Page/Group 2)	text
291	q10_5_3<br><br>Show the field ONLY if:<br>[language] = '1' and [consent] = '1'	(Facebook Page/Group 3)	text
292	q10_6<br><br>Show the field ONLY if:<br>[language] = '1' and [consent] = '1'	How do you prefer to get messages about HIV or TB prevention, testing and treatment? Tick all that apply	checkbox<br>1 q10_6___1 Radio<br>2 q10_6___2 Newspaper<br>3 q10_6___3 Short message service (sms)<br>4 q10_6___4 WhatsApp<br>5 q10_6___5 Social networking sites e.g. Facebook, twitter,<br>6 q10_6___6 Television<br>7 q10_6___7 Print magazines<br><br>Custom alignment: LV
293	endofint<br><br>Show the field ONLY if:<br>[q10_6(1)] <> ""	You have come to the end of our questionnaire. Thank you very much for participating in this survey. We really appreciate you spending your time on this survey. FINISH	descriptive
294	q1_1_nd<br><br>Show the field ONLY if:<br>[consent] = '1' and [language] = '3'	Section Header: *ISIGABA 1 : IMININGWANA YALAPHA OPHENDULAKO AHLALA KHONA*<br>Q1.1 : Bewuneminyaka emingaki ngelanga lakho lamabeletho lokugcina? (Iminyaka yophendulako)	text (number)
295	q1_2_nd<br><br>Show the field ONLY if:<br>[consent] = '1' and [language] = '3'	Q1.2 : Wabelethwa nini? (YYYY/MMM/DD)	text (date_dmy)<br>Field Annotation: @HIDEBUTTON
